# Integrating social behaviour, demography and disease dynamics in network models: applications to disease management in declining wildlife populations

**DOI:** 10.1098/rstb.2018.0211

**Published:** 2019-07-29

**Authors:** Matthew J. Silk, David J. Hodgson, Carly Rozins, Darren P. Croft, Richard J. Delahay, Mike Boots, Robbie A. McDonald

**Affiliations:** 1 Centre for Ecology and Conservation, University of Exeter, Penryn Campus, Penryn, UK; 2 Environment and Sustainability Institute, University of Exeter, Penryn Campus, Penryn, UK; 3 Department of Integrative Biology, University of California Berkeley, Berkeley, CA, USA; 4 Centre for Research in Animal Behaviour, University of Exeter, Exeter, UK; 5 National Wildlife Management Centre, Animal and Plant Health Agency, Nympsfield, UK

**Keywords:** social network, multilayer network, density-dependence, frequency-dependent, disease-induced extinction, conservation

## Abstract

The emergence and spread of infections can contribute to the decline and extinction of populations, particularly in conjunction with anthropogenic environmental change. The importance of heterogeneity in processes of transmission, resistance and tolerance is increasingly well understood in theory, but empirical studies that consider both the demographic and behavioural implications of infection are scarce. Non-random mixing of host individuals can impact the demographic thresholds that determine the amplification or attenuation of disease prevalence. Risk assessment and management of disease in threatened wildlife populations must therefore consider not just host density, but also the social structure of host populations. Here we integrate the most recent developments in epidemiological research from a demographic and social network perspective, and synthesize the latest developments in social network modelling for wildlife disease, to explore their applications to disease management in populations in decline and at risk of extinction. We use simulated examples to support our key points and reveal how disease-management strategies can and should exploit both behavioural and demographic information to prevent or control the spread of disease. Our synthesis highlights the importance of considering the combined impacts of demographic and behavioural processes in epidemics to successful disease management in a conservation context.

This article is part of the theme issue ‘Linking behaviour to dynamics of populations and communities: application of novel approaches in behavioural ecology to conservation’.

## 1. Introduction

Infectious disease can play an important role in the decline and extinction of wildlife populations [1,2]. For example, the emergence of Chytridiomycosis has been implicated in the rapid decline and extinction of many amphibian species [3,4]. Similarly, the emergence of devil facial tumour disease (DFTD), which was first described in only 1996, has led to a rapid decline in Tasmanian devil *Sarcophilus harrisii* populations, resulting in the species being categorized as Endangered on the IUCN Red List in 2008 [5,6].

The impact of infectious diseases on small or declining populations can arise from spillover and emergence of novel pathogens in a species (or population), or a sudden change in the epidemiology of an existing pathogen, caused by environmental change or demographic shifts [1,7]. Regardless of the pathogen involved, the links between host behaviour, host demography, and the transmission of infection, will determine the impact of disease at different stages of population decline [8]. For example, pathogen transmission is often considered to be either density-dependent or frequency-dependent—i.e. pathogen transmission rate is either a function of host density or not [9]—and this is fundamental to whether they can drive host populations to extinction [8,10,11]. Frequency-dependent transmission heightens the risk of disease-induced extinction because contact frequency between infectious and susceptible hosts does not change, and therefore transmission opportunities are not reduced, as the host population declines.

Network approaches allow the inclusion of host population structure into transmission models [12–15]. Recently, data from bio-logging devices have enabled the collection of increasingly comprehensive contact data in wildlife populations [16], which can be used effectively to parametrize these models [17–19]. Network models can have practical applications in understanding how a novel pathogen may spread through a population, or how changes in population structure brought about by population decline or environmental change may alter the transmission of existing pathogens [20]. However, exploiting these approaches to determine the most effective ways to manage disease from a conservation perspective remains challenging.

One potential problem with using a simple network modelling approach is that it may underestimate the influence of demographic changes on the spread of infection, and therefore the consequences of diseases at different stages of population decline. For example, if host contact networks were to become more clustered and modular as population density decreased then disease might be contained within a subset of these modules and therefore be more likely to die out at lower population densities (see [21]). However, it is possible for increased mortality caused by disease to result in increased birth rates through compensatory density-dependent recruitment (e.g. [22]), giving rise to an influx of new susceptible individuals into a population that can increase disease incidence and/or prevalence. Similarly, it might be possible for host social and spatial behaviour to be disrupted by disease or management interventions, resulting in changes to contact network structure that could increase disease prevalence directly by increasing transmission rates or indirectly by reducing the health of individuals [23,24]. Therefore, when developing longer-term network models of infection, combining knowledge on demography and social behaviour will be important in forecasting and managing wildlife disease in the face of population decline.

Here we synthesize some of the most recent developments in the application of social network approaches to studying wildlife disease dynamics, focusing on directly transmitted infections. We highlight the value of combining such approaches with demographic modelling to describe the temporal dynamics of infection in small or declining populations, and to inform the design of effective disease management interventions in threatened wildlife populations.

## 2. Network modelling of infection

The most effective approach to forecasting how pathogens might spread through and impact wildlife populations is to use simulation models of infection across empirically-derived networks [12,14]. When real-time data are available on the infection status of individuals it will also be possible to make inferences about the relationships between social network dynamics and the spread of infection using statistical network models [25,26]. These statistical models can be used to further refine the simulation-based epidemiological network models introduced above (e.g. [17]). In general, network models exploit the conventional susceptible–infected (SI) susceptible–infected–recovered (SIR), susceptible–infected–susceptible (SIS) and susceptible–exposed–infectious–recovered (SEIR) compartmental models. Instead of being used to form equations that can then be solved analytically or numerically, network models tend to use estimates of the probabilities of transitioning between compartments (states) to generate a series of stochastic simulations that provide information on the expected size of epidemics, variation in this outcome, and the sensitivity of it to changes in host or pathogen traits [12,14]. The models introduced above differ in the disease states they include and are broadly representative of the epidemiology of different pathogens. In an SI model individuals remain infected once contracting an infection. The SIR model adds a recovered (or removed) state in which individuals cannot be re-infected, representative of lifelong immunity once infected (or death). The SIS model is an alternative in which individuals can be re-infected on multiple occasions. The SEIR model is the most common example of a model with multiple stages of infection, in this case one stage (exposed) in which an individual is infected but not yet capable of transmitting infection to other individuals and another where it is infectious.

**Figure 1.**
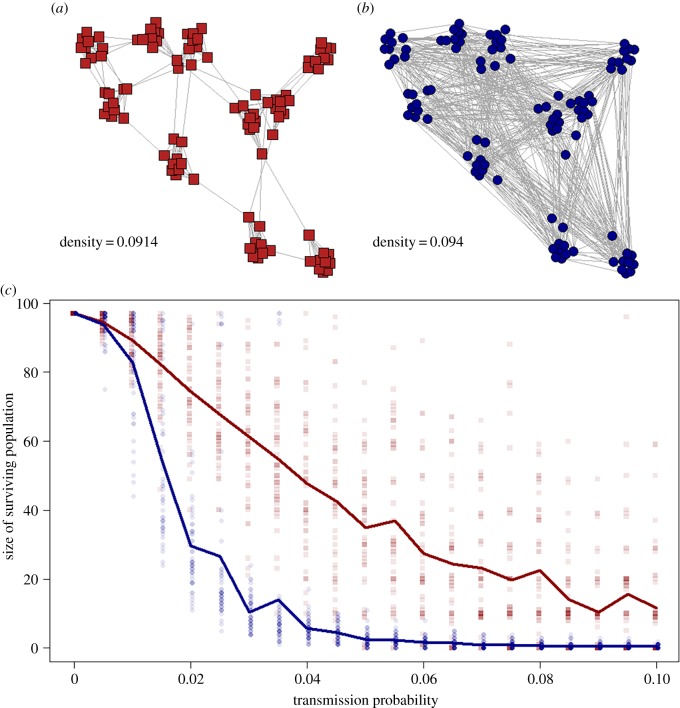
A comparison of the impact of infectious disease in two simulated small populations with different contact network structures (electronic supplementary material, 1.1 and 2). The networks of (*a*) population A and (*b*) population B have a similar edge density (proportion of dyads that are connected) but those in population A are considerably more modular. This results in considerable differences in the consequences of an epidemic in these populations, with (*c*) the surviving population after 300 model time-steps differing substantially between the two populations for pathogens with intermediate transmissibility. Points show results from 50 repeat simulations at each transmission probability and the lines connect the mean size of the surviving population for each population.

We provide a simple example of the use of an SIR model to understand the risks posed by disease transmission in a small and fragmented population (figure 1; electronic supplementary material, 1.1). The power of network analysis is apparent even in this basic example. Fragmented or modular networks can limit the spread of some infections [21]. As a result, the fragmented nature of the social network of population A (figure 1) results in the infection becoming trapped within certain regions of the network, with only the most infectious pathogens being able to spread more widely and cause complete or near extinction of the population. By contrast, in population B (figure 1) the more connected network structure means that all but the least transmissible pathogens cause substantial population declines. Therefore, in this particular case the network model predicts that social fragmentation may prevent disease-induced extinction of our hypothetical endangered species.

## 3. The importance of network dynamics in disease spread and control

Many network models of infection have considered pathogen transmission on a static contact network. Such an approach can provide an accurate model of real-world disease spread in some contexts, such as with fast spreading epidemics (e.g. see [27]), and has the advantages of being easier to parametrize from empirical data and less computationally intensive than models that account for network dynamics. However, animal social networks are dynamic. They will certainly change seasonally [17,18] and with population decline, and are likely to change in response to the acquisition of infection [28–30] and information [31]. In addition, individuals may adjust their existing social relationships in response to the death or removal of others from the population, resulting in further changes to local network structure [32,33]. Therefore, in the context of modelling disease spread, it often pays to model network dynamics explicitly alongside the spread of infection [34,35].

Theoretical models that incorporate network dynamics alongside transmission have provided insights into how changes in behaviour (especially in response to infection) can alter disease dynamics [36,37]. Infection avoidance behaviour (the response of other individuals towards an infected individual) and sickness behaviour (change in behaviour of an infected individual) can both have contrasting effects on disease dynamics. Infection avoidance behaviour, for example, is generally reported to increase epidemic thresholds, delay outbreaks and reduce disease prevalence [38–40]. One mechanism by which this has been found to occur is that infection avoidance behaviour can increase the modularity (or strength of subdivision) in networks with community structure, which can help ‘trap’ infection within a particular region of a network [41]. This might be particularly applicable to wild animals that live in social groups. However, under certain conditions, infection avoidance behaviour can aggravate epidemics. For example, if reductions in the strength of associations with infected individuals are mitigated by increasing the strength of associations with susceptible individuals, then epidemics can persist and spread further through a population [42]. Sickness behaviour has been less well studied but it is of evolutionary interest because it could be influenced by both the host (to reduce potentially infectious contacts; e.g. [29]) and the pathogen (to increase transmission opportunities; e.g. [43]). For example, changes in activity levels to reduce contact rates is a sickness behaviour that has often been considered to be adaptive for the host [44]. However, in some landscapes reduced activity can increase transmission under some environmental conditions (e.g. in water-limited landscapes; [45]). For some pathogens and parasites, understanding the combined impact of these behavioural dynamics alongside disease dynamics generated by demographic processes will be important to quantifying infectious disease threat for populations of conservation concern.

Understanding how changes in behaviour and social networks over time influence the spread of infection will also be critical to informing disease management interventions [13,34]. For example, in the case of vaccination, theoretical models suggest that control using vaccines can be more effective when host networks rewire adaptively in response to infection so that edges with infected individuals tend to be replaced with edges with uninfected individuals at a given rate [46]. By contrast, vaccination is likely to be less effective in networks that rewire at random as the network position of individuals will not be consistent. Similarly, incorporating an understanding of how networks might respond dynamically to interventions involving the selective removal of hosts can alter expectations of how likely these approaches are to succeed. This would typically require an expectation of, or relevant empirical data recording, behaviour change during and after an intervention. For example, culling-induced changes to social structure can exacerbate the spread of disease rather than limiting it as intended [23].

## 4. Integrating networks across multiple scales

Many threatened populations will occupy fragmented landscapes and occur in discontinuous sub-populations. Therefore, understanding the potential impact of infectious disease in these contexts may rely on integrating ideas from metapopulation dynamics, habitat connectivity and movement ecology into social network approaches. For example, low population connectivity can result in reduced epidemic coupling that promotes the global persistence of infection [47,48]. If this is the case, then altering vaccination strategies to consist of periodically pulsed mass vaccination can synchronize epidemics between subpopulations and facilitate disease eradication [47,48]. Subdivided populations and the movement of individuals within and between them can also be considered as a network [49]. Expanding network analyses to additionally consider the spatial arrangement and movements of infected and susceptible hosts is likely to be informative. Similar work on livestock movement networks has been effective in explaining disease dynamics in populations of domesticated animals (e.g. [50,51]).

**Figure 2.**
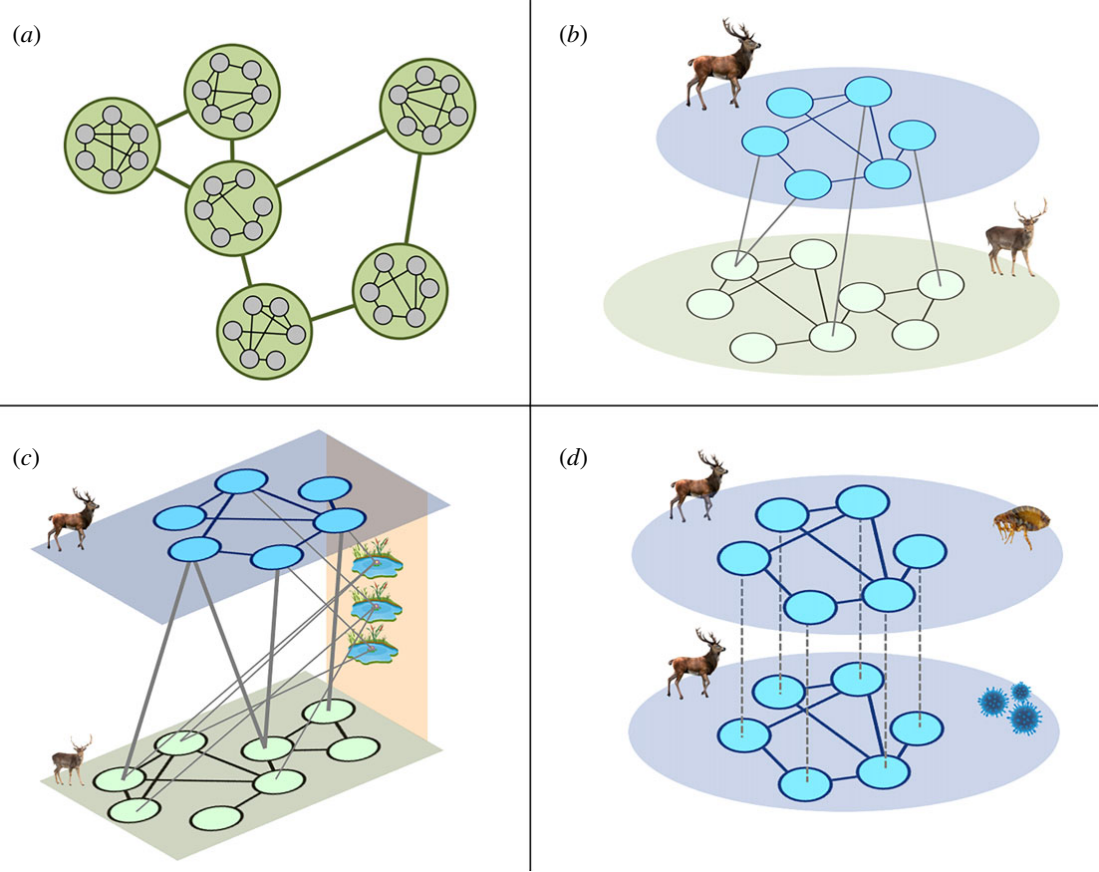
Multilayer representations of animal socio-spatial networks that may be applied to study disease transmission: (*a*) a network-of-networks that combines within patch social networks with between patch movement networks, (*b*) an interconnected network that combines intraspecific (within layer) and interspecific (between layer) interactions to describe potential transmission routes in a multi-host system, (*c*) an interconnected network that can integrate direct and indirect transmission in a multi-host system, and (*d*) a multiplex network that can combine transmission dynamics of different pathogens within the same model. These networks simply represent social interactions that may represent transmission opportunities, but this approach could be extended to transmission networks if the data were available.

Perhaps an even more powerful approach will be to integrate networks of different types of interactions across spatial scales. The advent of multilayer network analysis has resulted in a toolkit that can analyse complex systems containing multiple network types [52], with potential applications in ecology and animal behaviour [53–55]. Multilayer networks can take on a range of forms and we summarize three that are likely to be applied most usefully in epidemiological modelling here (figure 2): (i) a network of networks would make it possible to combine (sub-)population social networks with networks of dispersal movements or habitat connectivity that link these populations (figure 2*a*); (ii) interconnected networks would make it possible to consider how the transmission of pathogens between multiple hosts could exacerbate the disease threat posed to endangered populations (figure 2*b*), or an interconnected network framework can also be used to incorporate indirect transmission via the environment (figure 2*c*; [56]); and (iii) multiplex networks in which intra-layer connections represent transmission networks of different parasites could be used to consider the transmission of multiple pathogens simultaneously and determine whether co-infection may amplify the spread of a pathogen of interest (figure 2*d*). It is clear, therefore, that the multilayer network approach has considerable value for the study of wildlife disease and could be directly applicable in a conservation context through better integrating the role of alternative hosts and impact of interactions between multiple pathogens and/or parasites.

## 5. Integrating demographic and network modelling to study long-term disease dynamics

Network models have rarely been applied over the sort of timescales that require demographic components and are likely to be applicable in a conservation context. One notable exception is a model of DFTD in Tasmanian devils that used simulated networks parametrized using empirical data and combined this with empirically-derived demographic parameters [19]. Incorporating realistic network structure predicted slightly elevated risks of disease-induced extinction, and accelerated rates of host population decline.

More generally, incorporating demographic processes within network models is likely to provide important insights for wildlife disease management. Given that network models are typically simulation-based, incorporating a demographic component is relatively straightforward. In the Tasmanian devil example above [19] the central network model is a single static contact network, and when individuals are recruited (at age 2) they are assigned a fixed network position until they die. Mortality rates are coded separately for different disease states enabling the model to incorporate disease-induced mortality (clearly this last step is not necessary for all infections). The behavioural and demographic processes governing population structure are likely to be complex. A combined network-demographic-model such as this makes it possible to quantify how the social structure and life-history traits of threatened species might interact to determine the risk of disease-induced extinction from pathogens that differ in transmissibility and/or lethality.

Flexibility in life-history strategies has the potential to be able to buffer populations against disease. If recruitment is density-dependent then increased disease-induced mortality could result in increased recruitment, and as a result promote coexistence of host and pathogen populations [57]. For example, in European badgers *Meles meles* recruitment is density-dependent at a social group level. In populations naturally infected with *Mycobacterium bovis*, the causative agent of bovine tuberculosis, elevated mortality in infected social groups is compensated for by increased recruitment [22]. In cases where this buffering effect may play a role, incorporation of temporal variability in demographic processes into network models of infection can add vital nuance to the predicted effects of infection on population dynamics.

Density-dependence in disease transmission is another important demographic consideration. Depending on the life-history strategy of the pathogen involved, density-dependent (rather than frequency-dependent) transmission can prevent host extinctions being caused by disease alone. As a population declines, transmission rates also decline and may reach a point where the pathogen is unlikely to be maintained within the population unless it has alternative host species, is able to persist in the environment, persists in a covert/latent state or is able to re-infect hosts (i.e. individuals can recover from infection without becoming immune) [8]. Combined demographic and network models provide an opportunity to simulate how demographic processes influence social interactions and can therefore be used to vary between frequency-dependent and density-dependent transmission. For example, a contact network simulated with fixed rules for interaction probabilities that depended on home range overlap or distances between spatial centroids of home ranges (with home ranges not changing as the population declines), and therefore were lower in less dense populations, would result in a density-dependent transmission network. However, if a contact network were simulated in which individuals always maintained a fixed (or approximately fixed) number of contacts, then the behaviour of individuals would change as the population declined, transmission would be frequency-dependent and disease-induced extinction would be more likely [58]. The flexibility of the network approach means it can provide a predictive tool that extends across the spectrum from pure density-dependent to pure frequency-dependent transmission. Therefore, combining network and demographic modelling can accommodate uncertainty in how social structure and associated transmission opportunities changes at different stages of population decline.

**Figure 3.**
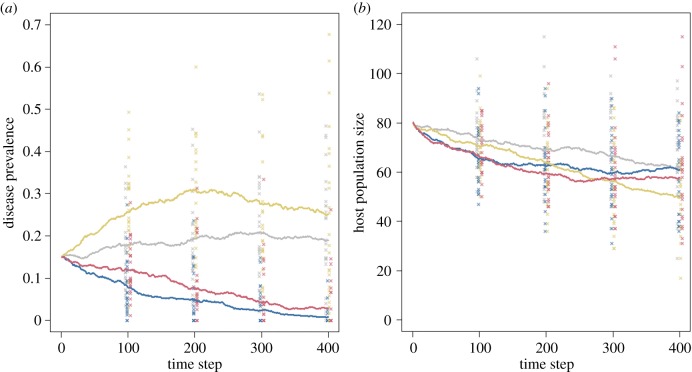
The effect of changes to pathogen-induced host mortality and changes to host social structure on (*a*) disease prevalence and (*b*) host population size in the SIR network model of an endemic infection presented in the electronic supplementary material, 1.2 (see box 1). The four scenarios presented are: no change (grey), increased pathogen-induced mortality (blue), increased social connectivity (fawn) and combined changes to mortality and host social structure (red). Lines represent the mean value at each time-step for each combination of the parameters and points show values from each of the first 25 simulation runs at time-steps 100, 200, 300 and 400. Points for the four scenarios are jittered on the *x*-axis for clarity. Increasing social connectivity has the greatest impact on host population size in this example because it maintains high pathogen prevalence.

We provide an example of a combined network-demographic model of infection (box 1; electronic supplementary material, 1.2). It incorporates a network-based SIR model alongside density-dependent recruitment to the population, in which individuals are more likely to recruit into smaller social groups. Having allowed the disease to become endemic in the population, we demonstrate the implications of sudden, potentially anthropogenic, changes in host social structure (increased probability of between-group contacts) and pathogen virulence or host tolerance (increased disease-induced mortality) separately and in combination (figure 3). These scenarios illustrate how social structure and the demographic response of the host population can be critical in determining the outcome of changes in pathogen dynamics. The model predicts that in this context a shift in both host behaviour and disease-induced mortality causes the most substantial population decline in the short-term, but that simply increased host social connectivity has more impact in the longer term by maintaining high pathogen prevalence. Sudden changes in disease dynamics are now more likely as a consequence of anthropogenic environmental change [59], and therefore modelling the potential consequences for endangered populations will become increasingly relevant.

Box 1. **A susceptible–infected–recovered (SIR) model to forecast impacts of environmental change (electronic supplementary material, 1.2 and 3).**

**Model outline.** An initial population consisting of 10 groups of 10 individuals with a modular social network structure was seeded with three initially infected individuals. In a given time-step: (i) infected individuals transmit stochastically according to their contact structure and a fixed transmission probability, (ii) mortality is simulated stochastically with separate probabilities controlling the baseline (susceptible) mortality and additional (disease-induced mortality), and (iii) recruitment is simulated stochastically using a fixed birth rate and the probability of being recruited into a group is inversely proportional to group size (encoding density-dependent recruitment). Once recruited, individuals' social connections were fixed, meaning that network dynamics were caused only by demographic processes, and meant that disease transmission was more density-dependent than frequency-dependent.

**Endemic phase.** The model was allowed to run for 800 time-steps with the original conditions to ensure that disease was endemic within the population.

**Change phase.** After 800 time-steps we set up four sets of prospective future conditions: (i) a control condition (no change to any parameters), (ii) increased pathogen virulence (a threefold increase in additional (disease-induced) mortality), (iii) increased connectivity (threefold increase in the probability of between-group connections in the social network that reduced network modularity), and (iv) increased pathogen virulence and increased connectivity. The two ‘treatments’ were designed to reflect potentially realistic future changes to the system; increased disease-induced mortality caused by increased pathogen virulence or reduced host tolerance, and increased connectivity caused by social perturbation in response to disturbance. After the change in parameters the simulation was run for 400 additional time-steps (50 repeat runs) and the results are shown in figure 3 and table 1. Changing both the social network and disease-induced mortality generates the greatest short-term population decline in this scenario. However, in the longer term increasing social connectivity without changing disease-induced mortality leads to more prolonged population decline as high prevalence is maintained.

**Table 1.** Outcomes of different changes to host–pathogen dynamics in our example model.

treatment	mean host population size after 200 time-steps (±s.e.)	mean prevalence after 200 time-steps (±s.e.)	mean host population size after 400 time-steps (±s.e.)	mean prevalence after 400 time-steps (±s.e.)
control	69 ± 2.3	0.19 ± 0.02	62 ± 2.7	0.19 ± 0.02
virulence change	63 ± 1.9	0.05 ± 0.01	61 ± 2.6	0.01 ± 0.003
behaviour change	64 ± 2.1	0.31 ± 0.02	50 ± 2.1	0.25 ± 0.03
virulence and behaviour change	59 ± 1.8	0.08 ± 0.01	58 ± 2.7	0.03 ± 0.01

## 6. Using a combined network and demographic approach to design management interventions

In many cases, intervention may be required to prevent the spread of infection in an endangered wildlife population. Management interventions can be categorized as approaches targeted at the host, the pathogen or the environment [60]. Using a combined network and demographic approach may aid the selection of appropriate management, as well as improving its effectiveness, especially when investigating selective interventions targeted at particular individuals.

In many small populations, culling of hosts is often not appropriate for control of disease, especially if it is non-selective, as it may be necessary to remove too many individuals and therefore contribute to the population becoming unviable [61]. For example, it has been suggested that culling is likely to be ineffective in controlling DFTD in Tasmanian devils owing to frequency-dependent transmission, fast life-histories (that limit density-dependent recruitment) and a pathogen with a long infectious period. These features mean that the level of culling required for disease control would probably be too damaging to the viability of the remaining population [61–63]. However, in some circumstances, using a network approach to guide selective culling towards those individuals most likely to contribute to the spread or persistence of infection, or in an attempt to subdivide the network at critical ‘cut-points’ [64], may allow the impact of the disease to be reduced while avoiding detrimental impacts on host population viability. This will probably be most feasible if strong density-dependence in recruitment buffers the population against the removal of individuals and if pathogen transmission is density-dependent, meaning that accounting for the demography and social networks of the host population is key.

**Figure 4.**
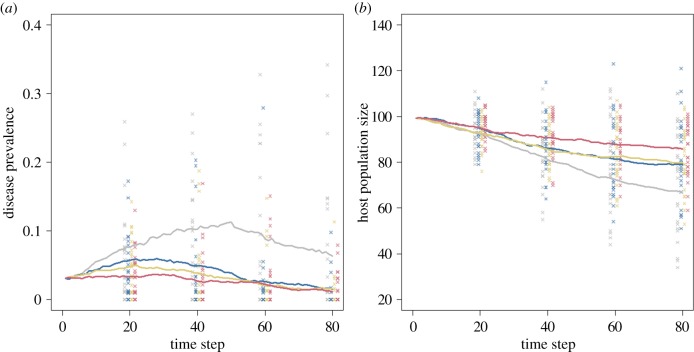
The effect of different vaccination programmes on (*a*) disease prevalence and (*b*) host population size in the SIR network model presented in the electronic supplementary material, 1.3 (see box 2). The four scenarios presented are: no vaccination (grey), random vaccination (blue), vaccination targeted at individuals with high degree (fawn) and vaccination targeted at individuals with high betweenness (red). In all programmes 20% of individuals are vaccinated and vaccine efficacy is assumed to be 100%. Lines represent the mean value at each time-step for each scenario and points show values from each of the first 25 simulation runs at time-steps 20, 40, 60 and 80. Points for the four scenarios are jittered on the *x*-axis for clarity. In this scenario, vaccinating 20% of the population is effective in reducing prevalence and maintaining a larger host population size and targeting vaccination at individuals with high betweenness is the most effective intervention.

Networks may provide critical insights into how best to target vaccination programmes in wildlife populations if individuals behave consistently over time. For example, in highly modular static networks, targeting vaccination at ‘bridge’ individuals (those that connect two or more social clusters) can greatly reduce the level of vaccination required to eradicate a disease or prevent it from spreading [65]. Similarly, in less clearly divided networks, targeting control measures at well-connected individuals is likely to have a disproportionate effect [66]. We demonstrate the potential effectiveness of network-targeted vaccination using a version of our dynamic network-demographic simulation model (figure 4 and box 2; electronic supplementary material, 1.3). We demonstrate an example scenario in which vaccination is used to protect a small population with a modular contact network from a novel highly infectious and virulent pathogen. Targeting vaccination at individuals important in connecting between groups in this scenario causes the greatest reductions in peak prevalence and does a better job at limiting population decline than random vaccination or vaccination targeted simply at the most connected individuals (figure 4 and table 2).

Box 2. **A susceptible–infected–recovered (SIR) model to forecast impacts of network-targeted vaccination (electronic supplementary material, 1.3 and 4).**

**Model outline.** The initial population and model structure were the same as that described in box 1. The only differences from that model were an increased transmission probability (7.5-fold increase) and additional (disease-induced) mortality (14-fold increase). This resulted in a much more rapid pathogen spread and considerable population decline if left unchecked.

**Epidemic phase.** Three individuals were initially infected, and infection was allowed to spread for five time-steps prior to detection (designed to replicate the likely lag between disease emergence and detection).

**Vaccination phase.** At the sixth time-step individuals were vaccinated according to four different programmes: (i) no vaccination (control), (ii) 20% of individuals vaccinated at random, (iii) 20% of individuals with the highest degree vaccinated, and (iv) 20% of individuals with the highest betweenness centrality vaccinated. Vaccine efficacy was assumed to be 100%, and vaccination was assumed to prevent infection if an individual selected was susceptible.

**Post-vaccination phase.** Subsequent to vaccination the model was run for an additional 79 time-steps (85 time-steps in total). The vaccination and post-vaccination phases were repeated 50 times. The results, which reveal that targeted vaccinations are slightly more effective than random vaccination with a less variable outcome, are presented in figure 4 and table 2.

**Table 2.** Outcomes of different vaccination programmes in our example model.

treatment	mean host population size after 40 time-steps (±s.e.)	mean prevalence after 40 time-steps (±s.e.)	mean host population size after 80 time-steps (±s.e.)	mean prevalence after 80 time-steps (±s.e.)
control	81 ± 1.8	0.103 ± 0.016	67 ± 3.2	0.063 ± 0.012
random	86 ± 1.7	0.050 ± 0.008	79 ± 2.5	0.015 ± 0.004
degree-targeted	86 ± 1.4	0.039 ± 0.007	80 ± 2.0	0.015 ± 0.005
betweenness-targeted	91 ± 1.2	0.027 ± 0.006	86 ± 2.1	0.011 ± 0.003

It is unlikely to always be feasible to target vaccinations based on known network positions. Quantifying empirical social networks is time-consuming and expensive [34], and may not be feasible for some highly endangered populations if trapping and/or tracking individuals is unduly risky to their health [67]. Therefore, identifying phenotypic traits that correlate with the social network positions of individuals represents an important alternative approach. One candidate is sex-biased variation in the epidemiology of pathogens. In European badgers, for instance, contact networks are structured at a broader spatial scale for males than females [68] and males are more likely to acquire infection and progress to advanced disease [69]. In host–pathogen systems with this sex-biased epidemiology, targeting vaccination at the sex that contributes most towards infection spread is likely to be more effective. Similarly, the feasibility of targeting selective management interventions based on network position will depend on how dynamic social network structure is, and the impact of this on the relative importance of well-connected or ‘bridge’ individuals. If network structure changes substantially with population size, or if individuals are not consistent in their network position, then targeted management interventions become more difficult to implement successfully.

Network approaches can also be used to guide management interventions aimed at modifying the environment. Supplemental feeding of wildlife, for instance, can alter transmission dynamics by altering social network structure and/or providing well-connected hubs of indirect environmental transmission [70]. Therefore, changing patterns of supplementary feeding can influence social network structure which may in turn increase (or decrease) transmission. For example, higher feeder density has been shown to result in elevated transmission of *Mycoplasma gallisepticum* in house finches *Haemorhous mexicanus* [71]. This potential impact of supplementary feeding might be an important consideration when applied to populations of conservation concern. Similarly, the isolation of infected sub-populations via targeting of the environment might be achieved more effectively using information on movement and habitat networks within a population.

## 7. Conclusion and further work

Disease can cause or contribute to the decline and extinction of threatened species, and the threat posed may be exacerbated by anthropogenically-driven environmental change resulting in the emergence of novel pathogens or changes in the epidemiology of existing diseases. Developing an understanding of how population dynamics, population connectivity and social behaviour interact to determine the vulnerability of small and declining populations to new and existing pathogens will be crucial to recognizing and developing effective means of managing such threats. New developments in epidemiological modelling that combine social networks and demographic parameters offer a predictive framework that may be instrumental in achieving this goal.

## Supplementary Material

Supplementary Methods

## Supplementary Material

Epidemiological Network Models for Conservation Supplementary Material 2

## Supplementary Material

Epidemiological Network Models for Conservation Supplementary Material 3

## Supplementary Material

Epidemiological Network Models for Conservation Supplementary Material 3

## Data accessibility

Code for all simulated models is provided in the electronic supplementary material.
