# Supplementary material for: Integrating social behaviour, demography and disease dynamics in network models: applications to disease management in declining wildlife populations
Source: Philos Trans R Soc Lond B Biol Sci. 2019 Jul 29;374(1781):20180211. doi: 10.1098/rstb.2018.0211 (PMC6710568; doi:10.1098/rstb.2018.0211)
Supplement: Supplementary Methods [file rstb20180211supp1.docx]

**Integrating behaviour, demography and disease dynamics through network models: applications to disease management in declining wildlife populations**

**Supplementary Material 1**

**SM 1.1. Basic epidemiological network model in R**

**Methods**

Full R code is provided in the Supplementary Material 2 and is designed to be interactive. Here we describe the principal methodologies and results used in the main text.

**Population generation**

Populations are generated by an algorithm that defines a number of groups, number of individuals per group and a number of initially infected individuals. Groups are assigned locations in 2D space (locations are used to generate social networks subsequently). All individuals are identical (i.e. no additional individual characteristics are defined) but are assigned vectors recording whether they are susceptible, infected or removed (dead). The identity of the initially infected individuals is determined randomly.

**Network generation**

An unweighted social network is generated using information on group membership and the distance between groups. The p.ig parameter defines the probability of within-group edges, the p.og parameter defines the baseline probability of between-group edges and the dist.eff parameter defines how the probability of between-group edges changes with the distance between groups. The probability of between-group edges is therefore $P\left( Edge \right)=p.og(e^{dist.eff*Distance}$).

**Simulated infection**

We applied a stochastic SIR model of infection. The transmission algorithm used a probability of becoming infected (S_I) if connected in a network and probability of recovering (I_R) that apply per time-step. First, a transmission matrix (t.mat) was created using the S_I parameter that recorded whether transmission would occur ***if*** two individuals (one susceptible, one infected) were connected in the social network. Second, it was determined using the transmission matrix and social network which new individuals had become infected and the status of all individuals was updated. Third, all infected individuals had the opportunity to become removed (dead) using the I_R parameter. The status of all individuals was then updated.

**Our analysis**

We generated a single baseline population of 100 individuals in 10 groups of 10 individuals. We then generated two social network structures (Fig. 1) using the parameters provided in Table S1.1a. Finally, we simulated the spread of infection in these populations over 300 time steps using the parameters provided in Table S1.1a. Three individuals are infected initially. We repeated the disease transmission step 50 times in order to produce Figure 1 in the main text.

| **Parameter** | **Population A** | **Population B** |
| --- | --- | --- |
| p.ig | 0.95 | 0.12 |
| p.og | 0.035 | 0.1 |
| dist.eff | -0.35 | -0.0005 |
| S_I | 0-0.1 in increments of 0.005 | 0-0.1 in increments of 0.005 |
| I_R | 0.08 | 0.08 |

Table S1.1a. Parameters used to simulate the spread of infection in two model host populations.

**SM 1.2. Combined network-demographic model and an application to forecasting the impacts of environmental change**

**Methods**

Full R code is provided in the Supplementary Material 3. Here we describe the principal methodologies and results used in the main text.

**Population generation**

The generation of the initial population and social network used the same algorithms as those described in SM1.1. The initial population consisted of 10 groups of 10 individuals. The parameters used in each stage of the analysis are provided in Table S1.2a.

**Demographic parameters**

To introduce a demographic component to the model we included two additional parameters. We added a probability (S_R) that hosts could transition directly from susceptible to removed (dead), and altered the I_R parameter so that it instead determined additional mortality caused by the pathogen (i.e. the probability of a transition from infected to removed states was S_R+I_R). We additionally added a birth rate (BR). In our example recruitment was density dependent at a population and social group level. The overall number of recruits was determined by the total population size. The probability of these recruits occurring within a particular group was inversely related to the size of that group whereby new recruits were more likely to be assigned to smaller groups (as long as the group size was not zero).

**Simulation details**

At each timestep the algorithm proceeded stochastically as follows:

1. Transmission occurs (as detailed in SM 1.1) – opportunity for transition from susceptible to infected if connected to an infected individual with a probability S_I for each connection.
2. Transition to removed state occurs. Probability of transition to removed state is S_R for susceptible individuals and S_R+I_R for infected individuals
3. New individuals are recruited into the population. Recruitment is stochastic with the number of recruits being the sum of a Bernoulli draw with size equal to the population size and probability equal to the birth rate.
4. New recruits are assigned groups. During this assignment groups are sampled with replacement and the probability of being sampled is proportional to the inverse of group size (meaning that individuals are more likely to be recruited into smaller groups).
5. New recruits are wired into the population social network using the same edge probabilities as those used to generate the initial population social network.

**Endemic phase**

Having initiated the population with parameters set as per Table S1.2a and three initially infected individuals, we simulated through 800 timesteps to ensure that the disease was endemic in the population with fairly low prevalence.

**Change phase**

After 800 timesteps we set up four prospective sets of future conditions with different parameter sets and continued the simulation for a further 400 timesteps. In condition 1 (control condition) no model parameters were altered. In condition 2 (increased virulence) the impact of being infected on mortality was increased substantially to replicate a context when a change in environmental conditions results in either increased pathogen virulence or reduced host tolerance to infection. In condition 3 (increased connectivity) the host social network structure was changed so that there was a greater probability of edges being formed between individuals in different social groups. This would be expected to result in a less modular social network that represented less of a barrier to the spread of infection. In condition 4 we increased both pathogen virulence and host social connectivity. Each was increased by the same amount as when they were changed independently. Details of the changed parameters are provided in Table S1.2b. We conducted 50 repeat runs of each condition.

**Results**

We recorded data on disease prevalence and host population size in all 400 timesteps subsequent to the change in conditions. In the main text (Fig. 3) we show the mean of all 50 repeats of each condition as lines, and at 4 separate time points show the raw values of each run of the simulation.

Table S1.2a. Full parameter set used to model the initial endemic phase of infection for example 2.

| **Parameter** | **Value** |
| --- | --- |
| p.ig | 0.65 |
| p.og | 0.01 |
| dist.eff | -0.3 |
| S_I | 0.008 |
| S_R | 0.01 |
| I_R | 0.005 |
| BR | $S_{R}\times1.01+ \frac{100-Pop.Size}{1000}$ |

Table S1.2b Details on the parameters changed in each prospective future condition

| **Scenario** | **I_R** | **p.og** |
| --- | --- | --- |
| No change | 0.005 | 0.01 |
| Increased virulence | 0.015 | 0.01 |
| Increased connectivity | 0.005 | 0.03 |
| Increased virulence and connectivity | 0.015 | 0.03 |

**SM 1.3. Combined network-demographic model and an application to forecasting the effect of targeted vaccination against a new epidemic.**

**Methods**

Full R code is provided in the Supplementary Material 4. Here we describe the principal methodologies and results used in the main text.

**Population generation**

The generation of the initial population and social network used the same algorithms as those described in SM1.1. The demographic parameters used are the same as those detailed in SM 1.2. They key difference in this example is that we simulated a pathogen that was much more infectious and resulted in greatly elevated mortality. The initial population consisted of 10 groups of 10 individuals. The parameters used in each stage of the analysis are provided in Table S1.3a.

**Initial epidemic phase**

Three individuals were infected initially. Following initiation of infection in the population we ran the initial simulation for five timesteps to allow the infection to establish itself within a region of the network. This was intended to replicate the emergence and pre-detection spread of a harmful novel pathogen in a population.

**Vaccination phase**

At the sixth timestep we altered the transmission model so that it also contained a vaccinated state (V). Individuals could only transition into the vaccinated state from the susceptible state. Once vaccinated they could not become infected with the pathogen and were removed the population with the same probability as susceptible individuals (S_R).

In timestep 6 transmission and recruitment stages of the algorithm were conducted as normal. Then the population was vaccinated with four different programmes: 1) no vaccination (control), 2) 20% of the population vaccinated at random, 3) the 20% of individuals with the highest degree being vaccinated (i.e. the most well-connected or “hub” individuals), and 4) the 20% of individuals with the highest betweenness centrality (i.e. the individuals with the greatest number of shortest paths between other nodes that passed through them, a proxy for “bridge” individuals connecting between different groups). For the purposes of this example, vaccine efficacy was assumed to be 100%, but this can be altered in the R functions provided if desired.

**Post-vaccination phase**

The model with the new vaccinated state was run for a further 79 timesteps (85 timesteps in total) without any further vaccination being carried out. The vaccination and post-vaccination phases were repeated 50 times for each vaccination programme.

**Results**

We recorded data on disease prevalence and host population size in all 80 timesteps subsequent to and including timestep 6 (the vaccination phase and post-vaccination phase). In the main text (Fig. 4) we show the mean of all 50 repeats of each vaccination programme as lines, and at 4 separate time points show the raw values of each run of the simulation.

Table S1.3a. Full parameter set used to model the emerging epidemic for example 3.

| **Parameter** | **Value** |
| --- | --- |
| p.ig | 0.5 |
| p.og | 0.03 |
| dist.eff | -0.4 |
| S_I | 0.06 |
| S_R | 0.01 |
| I_R | 0.07 |
| BR | $S_{R}\times1.01+ \frac{100-Pop.Size}{1000}$ |
