# Supplementary material for: Integrating social behaviour, demography and disease dynamics in network models: applications to disease management in declining wildlife populations
Source: Philos Trans R Soc Lond B Biol Sci. 2019 Jul 29;374(1781):20180211. doi: 10.1098/rstb.2018.0211 (PMC6710568; doi:10.1098/rstb.2018.0211)

Basic SIR network model of infection

Matthew Silk

11 June 2018

In this document we provide the code for a basic network model of infection. By playing with the parameters provided it will be possible to completely alter the contact network structure and the characteristics of the disease and explore the consequence of this for population persistence.

The code provided at the bottom generates (and saves) an animation of the model. It requires Image Magick to be installed on the computer that the code is being run on. Not having this functioning will not affect the ability to run the rest of the code.

First load the required packages and set the path to Image Magick

It is necessary to install the "animation" package directly from github to avoid a problematic bug

```
Sys.setenv(PATH = paste("C:\\Program Files\\ImageMagick\\bin",Sys.getenv("PATH"), sep = ";"))

library(devtools)
install_github("yihui/animation")

library(animation)
library(igraph)
```

We now define a function (pop.gen) that generates a population consisting of ***n.groups*** subpopulations of ***s.groups*** size. ***n.I*** individuals are initially infected

```
#Function to set up a population

pop.gen<-function(n.groups=10,s.groups=10,n.I=1){

 # defines location of groups
 poss.x<-rep(seq(1,n.groups,1),each=n.groups)
 poss.y<-rep(seq(1,n.groups,1),n.groups)
 poss.locs<-cbind(poss.x,poss.y)
 locs<-sample(seq(1,n.groups^2,1),n.groups,replace=F)
 group.locs<-data.frame(seq(1,n.groups,1),poss.locs[locs,])

 #calculates population size
 pop<-n.groups*s.groups

 #creates individuals
 indiv.ID<-seq(1,pop,1)

 #assigns individuals to groups
 indiv.GR<-rep(1:n.groups,each=s.groups)

 #assigns individuals their correct group locations
 indiv.X<-rep(NA,pop)
 indiv.Y<-rep(NA,pop)
 for(i in 1:pop){
  indiv.X[i]<-group.locs[group.locs[,1]==indiv.GR[i],2]
  indiv.Y[i]<-group.locs[group.locs[,1]==indiv.GR[i],3]
 }

 #creates dataframe containing population info
 indiv.info<-data.frame(indiv.ID,indiv.GR,indiv.X,indiv.Y)
 names(indiv.info)<-c("ID","Group","X","Y")

 #create initially infected individuals and generate SIR + D info
 I.I<-sample(1:pop,n.I)
 I<-matrix(0,nr=pop,nc=1)
 I[I.I]<-1
 S<-1-I
 R<-matrix(0,nr=pop,nc=1)

 #combine into dataframe and return dataframe and group locations as a list
 indiv.info<-data.frame(indiv.info,S,I,R)

 p<-list(indiv.info,group.locs)

 return(p)

}
```

We then define a function that uses the population information (*pop* and *indiv.info*) and a set of edge probabilities (*p.ig*,*p.og*,*dist.eff*) to generate a contact network for the population. Setting plot=T will provide an image of the network generated

- **p.ig** is the probability of within subpopulation edges
- **p.og** is the probability of between subpopulation edges
- **dist.eff** is the effect of distance between subpopulations on the probability of between subpopulation edges

```
net.gen<-function(pop,indiv.info,p.ig,p.og,dist.eff,plot=T){

 network<-matrix(0,nr=pop,nc=pop)

 rownames(network)<-colnames(network)<-indiv.info$ID

 for(i in 1:(nrow(network)-1)){
  for(j in (i+1):nrow(network)){
   if(indiv.info$Group[indiv.info$ID==rownames(network)[i]]==indiv.info$Group[indiv.info$ID==colnames(networ
k)[j]]){
    tmp<-p.ig
    network[i,j]<-rbern(1,tmp)
   }
   if(indiv.info$Group[indiv.info$ID==rownames(network)[i]]!=indiv.info$Group[indiv.info$ID==colnames(networ
k)[j]]){
    tmp.d<-dist(group.locs[c(indiv.info$Group[indiv.info$ID==rownames(network)[i]],
                             indiv.info$Group[indiv.info$ID==colnames(network)[j]]),2:3])
    tmp<-p.og*exp(dist.eff*tmp.d)
    network[i,j]<-rbern(1,tmp)
   }

   network[j,i]<-network[i,j]

  }
 }

 diag(network)<-0

 if(plot==T){
 dev.new()
  plot(graph.adjacency(network,mode="undirected"),vertex.color=pop.info[[1]]$I,vertex.label=NA,vertex.size=4
)
 }

 return(network)

}
```

We now write a function that governs the disease transmission process occurring at each time step. This function requires information on the population, the contact network, and the groups and their locations.

It also uses two dsease parameters

- ***S_I*** governs the probability per time-step of a susceptible individual becoming infected if connected to an infected node in the contact network
- ***I_R*** governs the probability per time-step of a susceptible individual becoming infected if connected to an infected node in the contact network

```
ts<-function(network,indiv.info,n.groups,group.locs,S_I,I_R,plot=T){

 S<-indiv.info$S
 I<-indiv.info$I
 R<-indiv.info$R

 t.mat<-array(0,dim=dim(network))
 for(i in 1:nrow(network)){
  for(j in 1:nrow(network)){
   t.mat[i,j]<-rbern(1,S_I)*network[i,j]
  }
 }

 diag(t.mat)<-0

 danger<-t.mat[which(I>0),]
 ifelse(is.vector(danger)==TRUE,infected<-which(danger>0),infected<-which(colSums(danger)>0))

 if(length(infected)>0){
  for(i in 1:length(infected)){
   if(R[infected[i]]==0){
    I[infected[i]]<-1
    S[infected[i]]<-0
   }
  }
 }

 for(i in 1:nrow(indiv.info)){
  if(I[i]==1){
   R[i]<-rbern(1,I_R)
   if(R[i]==1){
    I[i]<-0
   }
  }
 }

 indiv.info2<-indiv.info
 indiv.info2$S<-S
 indiv.info2$I<-I
 indiv.info2$R<-R

 res<-list(indiv.info2)
 return(res)

} #end function
```

Define a Bernoulli draw function for convenience

```
rbern<-function(n,prob){
  return(rbinom(n,1,prob))
}
```

In this section of the code we define the key parameters - these are the ones to change to adjust network and disease characteristics

```
#PROBABILITY OF WITHIN SUBPOPULATION CONTACTS
p.ig<-0.6

#PROBABILITY OF BETWEEN SUBPOPULATION CONTACTS
p.og<-0.025

#DISTANCE EFFECT ON BETWEEN SUBPOPULATION CONTACTS
dist.eff<- -0.3 #plugged into exponential decay

#PROBABILITY OF SUCEPTIBLE INDIVIDUAL BEING INFECTED OVER AN EDGE PER TIME-STEP
S_I<-0.04

#PROBABILITY OF RECOVERY/DEATH PER TIME-STEP
I_R<-0.03
```

We now set up the population and its contact network. The population is 100 individuals, consisting of 10 subpopulations of 10 individuals.

```
#create empty list to store outputs every time step
pop.info<-list()

n.groups<-10
s.groups<-10

#create initial population
tmp.p<-pop.gen(n.groups,s.groups,n.I=3)

#and store the output
pop.info[[1]]<-tmp.p[[1]]
group.locs<-tmp.p[[2]]

#create intitial network
network<-net.gen(pop=nrow(pop.info[[1]]),indiv.info=pop.info[[1]],p.ig,p.og,dist.eff,plot=T)
```

We now run transmission (and tranisition to recovered state) for 300 time-steps (we conduct the second time-step separately the rest to facilitate further checking if desired)

```
tmp.up<-ts(network=network,indiv.info=pop.info[[1]],n.groups=n.groups,group.locs=group.locs,S_I=S_I,I_R=I_R,
plot=F)

pop.info[[2]]<-tmp.up[[1]]
network<-network

for(t in 3:300){
 tmp.up<-ts(network=network,indiv.info=tmp.up[[1]],n.groups=n.groups,group.locs=group.locs,S_I=S_I,I_R=I_R,p
lot=F)
 pop.info[[t]]<-tmp.up[[1]]
}
```

We now create a series of summary plots to illustrate the spread of the disease

1. A plot of the percentage of individuals in the susceptible, infected and recovered states over time
2. A plot of prevalence per subpopulation to demonstrate the spatial variability in outbreak timing and severity
3. A multipanel network plot demonstrating the spread of infection at different stages of the simulation. Grey nodes are susceptible individuals, orange nodes are infected individuals and red nodes are recovered/dead individuals.

```
summaries<-lapply(pop.info,colSums)

sus<-numeric()
inf<-numeric()
rec<-numeric()

for(i in 1:300){
 sus[i]<-summaries[[i]][5]
 inf[i]<-summaries[[i]][6]
 rec[i]<-summaries[[i]][7]
}

plot(sus,xlim=c(0,300),ylim=c(0,100),type="l",col="blue",lwd=3,ylab="Percentage of population",xlab="Time st
eps",cex.lab=1.6,cex.axis=1.3,las=1)
lines(inf,col="orange",lwd=3)
lines(rec,col="red",lwd=3)
```

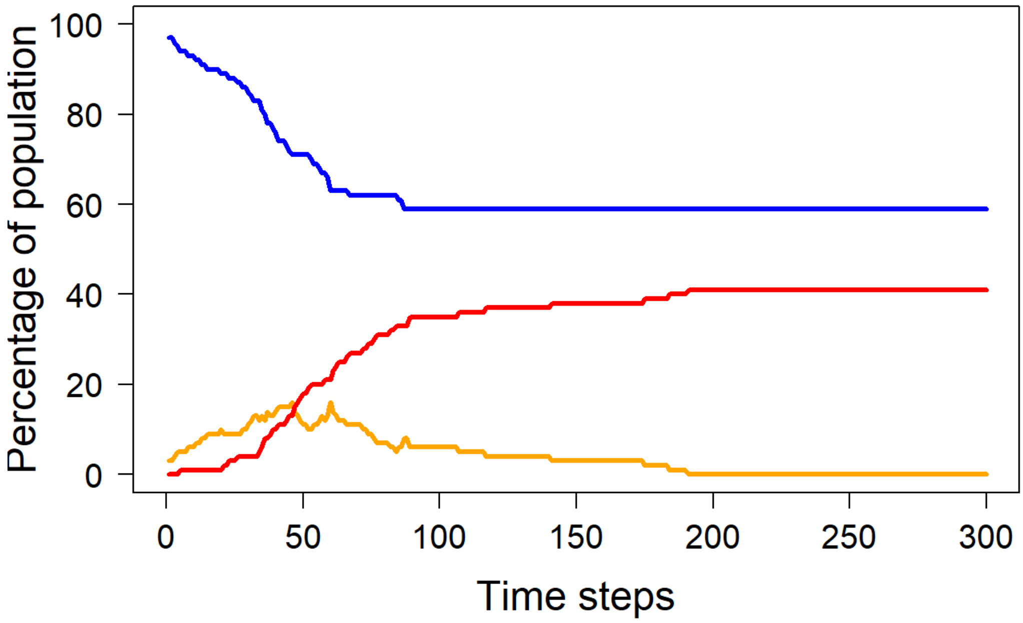


```
group.prev<-matrix(NA,nr=300,nc=10)

for(i in seq(1,300,1)){
 tmp<-aggregate(pop.info[[i]][,6],list(pop.info[[i]]$Group),mean)
 for(j in 1:nrow(tmp)){
  group.prev[i,as.numeric(as.vector(tmp)[j,1])]<-as.vector(tmp)[j,2]
 }
}

par(mfrow=c(2,5))
for(i in 1:10){
 plot(group.prev[,i],type="l",col="navy blue",lwd=2,lty=1,xlim=c(0,300),ylim=c(0,1),xlab="Timesteps",
      ylab="Prevalence",main=paste("Group",i),cex.main=1.5,cex.axis=1.3,cex.lab=1.5,las=1)
}
```

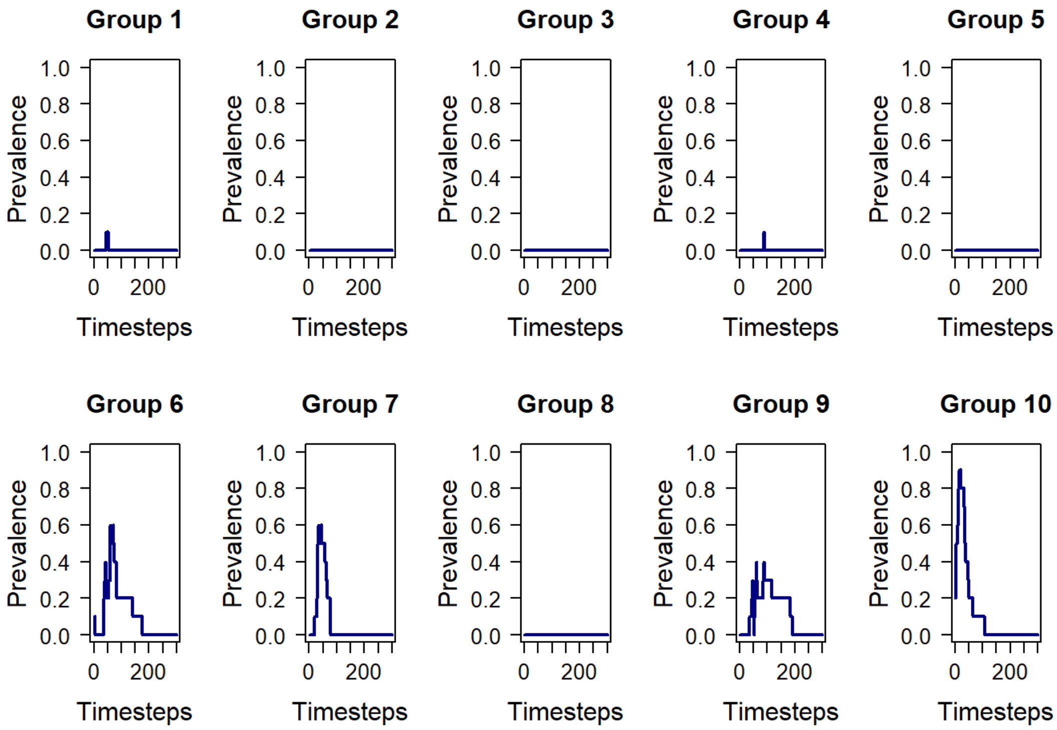

```
par(mfrow=c(1,1))

layout.n<-layout.auto(graph.adjacency(network,mode="undirected"))

par(mfrow=c(3,3),mar=c(0.5,0.5,1,0.5))
plot(graph.adjacency(network,mode="undirected"),
     vertex.color=ifelse(pop.info[[1]]$I==1,"orange",ifelse(pop.info[[1]]$R==1,"red","grey")),
     layout=layout.n,vertex.label=NA,vertex.size=8,main="1",cex.main=2)
plot(graph.adjacency(network,mode="undirected"),
     vertex.color=ifelse(pop.info[[38]]$I==1,"orange",ifelse(pop.info[[38]]$R==1,"red","grey")),
     layout=layout.n,vertex.label=NA,vertex.size=8,main="38",cex.main=2)
plot(graph.adjacency(network,mode="undirected"),
     vertex.color=ifelse(pop.info[[75]]$I==1,"orange",ifelse(pop.info[[75]]$R==1,"red","grey")),
     layout=layout.n,vertex.label=NA,vertex.size=8,main="75",cex.main=2)
plot(graph.adjacency(network,mode="undirected"),
     vertex.color=ifelse(pop.info[[113]]$I==1,"orange",ifelse(pop.info[[113]]$R==1,"red","grey")),
     layout=layout.n,vertex.label=NA,vertex.size=8,main="113",cex.main=2)
plot(graph.adjacency(network,mode="undirected"),
     vertex.color=ifelse(pop.info[[150]]$I==1,"orange",ifelse(pop.info[[150]]$R==1,"red","grey")),
     layout=layout.n,vertex.label=NA,vertex.size=8,main="150",cex.main=2)
plot(graph.adjacency(network,mode="undirected"),
     vertex.color=ifelse(pop.info[[188]]$I==1,"orange",ifelse(pop.info[[188]]$R==1,"red","grey")),
     layout=layout.n,vertex.label=NA,vertex.size=8,main="188",cex.main=2)
plot(graph.adjacency(network,mode="undirected"),
     vertex.color=ifelse(pop.info[[225]]$I==1,"orange",ifelse(pop.info[[225]]$R==1,"red","grey")),
     layout=layout.n,vertex.label=NA,vertex.size=8,main="225",cex.main=2)
plot(graph.adjacency(network,mode="undirected"),
     vertex.color=ifelse(pop.info[[263]]$I==1,"orange",ifelse(pop.info[[263]]$R==1,"red","grey")),
     layout=layout.n,vertex.label=NA,vertex.size=8,main="263",cex.main=2)
plot(graph.adjacency(network,mode="undirected"),
     vertex.color=ifelse(pop.info[[300]]$I==1,"orange",ifelse(pop.info[[300]]$R==1,"red","grey")),
     layout=layout.n,vertex.label=NA,vertex.size=8,main="300",cex.main=2)
```

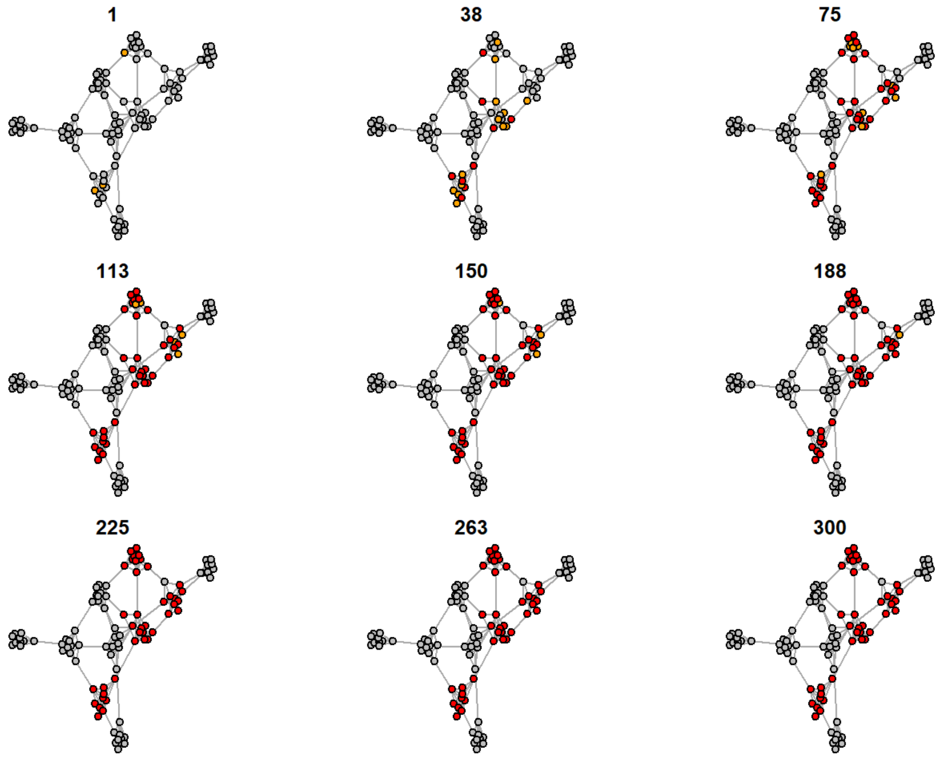

Finally we are going to attempt to generate a GIF of the entire simulation (although this can be tempramental) *It will be necessary to change the path to the file in the saveGIF function*

```
convert_path<-paste0(shortPathName("C:\\Program Files\\ImageMagick-7.0.7-Q16\\"),"convert.exe")
convert_path2<-"C:/Program Files/ImageMagick-7.0.7-Q16/convert.exe"
ani.options(convert=convert_path2)
saveGIF(
#loop through plots
for(i in 1:300){
  par(mfrow=c(1,1),mar=c(0.5,0.5,1,0.5))
  plot(graph.adjacency(network,mode="undirected"),vertex.color=ifelse(pop.info[[i]]$I==1,"orange",ifelse(pop
.info[[i]]$R==1,"red","grey")),layout=layout.n,vertex.label=NA,vertex.size=8,main=paste(i),cex.main=2)
},
interval=0.2,
ani.width=400,
ani.height=400,
movie.name = "C:/Users/matth/Dropbox/PhilTransSim1.gif")
```

```
## [1] FALSE
```

And here is one we made earlier….

file:///C:/Users/matth/Dropbox/PhilTransSim1.gif

Here we provide the analysis used for Fig. X in the paper. Vary transmission probabilities for two different types of social network. A) Modular (highly subdivided) and B) a network of a similar density but with much weaker subdivisions

We keep the recovery probability the same here but provide the code so that can people can explore the full parameter space for themselves

First set a seed for this section to ensure repeatability

```
set.seed(2)
```

Choose set of parameters for network A)

```
#PROBABILITY OF WITHIN SUBPOPULATION CONTACTS
p.ig<-0.95

#PROBABILITY OF BETWEEN SUBPOPULATION CONTACTS
p.og<-0.035

#DISTANCE EFFECT ON BETWEEN SUBPOPULATION CONTACTS
dist.eff<- -0.35 #plugged into exponential decay

#PROBABILITY OF SUCEPTIBLE INDIVIDUAL BEING INFECTED OVER AN EDGE PER TIME-STEP
S_I<-seq(0,0.1,0.005)

#PROBABILITY OF RECOVERY/DEATH PER TIME-STEP
I_R<-0.08
```

We now set up the population and its contact network. The population is 100 individuals, consisting of 10 subpopulations of 10 individuals.

```
#create empty list to store outputs every time step
pop.info<-list()

n.groups<-10
s.groups<-10

#create initial population
tmp.p<-pop.gen(n.groups,s.groups,n.I=3)

#and store the output
pop.info[[1]]<-tmp.p[[1]]
group.locs<-tmp.p[[2]]

#create intitial network
network<-net.gen(pop=nrow(pop.info[[1]]),indiv.info=pop.info[[1]],p.ig,p.og,dist.eff,plot=T)
```

```
ovr.dat<-list()
group.prevs<-list()

for(ip in 1:length(S_I)){

pop.info[[1]]<-tmp.p[[1]]
group.locs<-tmp.p[[2]]

S_I2<-S_I[ip]

tmp.up<-ts(network=network,indiv.info=pop.info[[1]],n.groups=n.groups,group.locs=group.locs,S_I=S_I2,I_R=I_R
,plot=F)

pop.info[[2]]<-tmp.up[[1]]
network<-network

for(t in 3:300){
 tmp.up<-ts(network=network,indiv.info=tmp.up[[1]],n.groups=n.groups,group.locs=group.locs,S_I=S_I2,I_R=I_R,
plot=F)
 pop.info[[t]]<-tmp.up[[1]]
}

summaries<-lapply(pop.info,colSums)

sus<-numeric()
inf<-numeric()
rec<-numeric()

for(i in 1:300){
 sus[i]<-summaries[[i]][5]
 inf[i]<-summaries[[i]][6]
 rec[i]<-summaries[[i]][7]
}

plot(sus,xlim=c(0,300),ylim=c(0,100),type="l",col="blue",lwd=3,ylab="Percentage of population",xlab="Time st
eps",cex.lab=1.6,cex.axis=1.3,las=1)
lines(inf,col="orange",lwd=3)
lines(rec,col="red",lwd=3)

ovr.dat[[ip]]<-data.frame(sus,inf,rec)

group.prev<-matrix(NA,nr=300,nc=10)

for(i in seq(1,300,1)){
 tmp<-aggregate(pop.info[[i]][,6],list(pop.info[[i]]$Group),mean)
 for(j in 1:nrow(tmp)){
  group.prev[i,as.numeric(as.vector(tmp)[j,1])]<-as.vector(tmp)[j,2]
 }
}

group.prevs[[ip]]<-group.prev

}
```

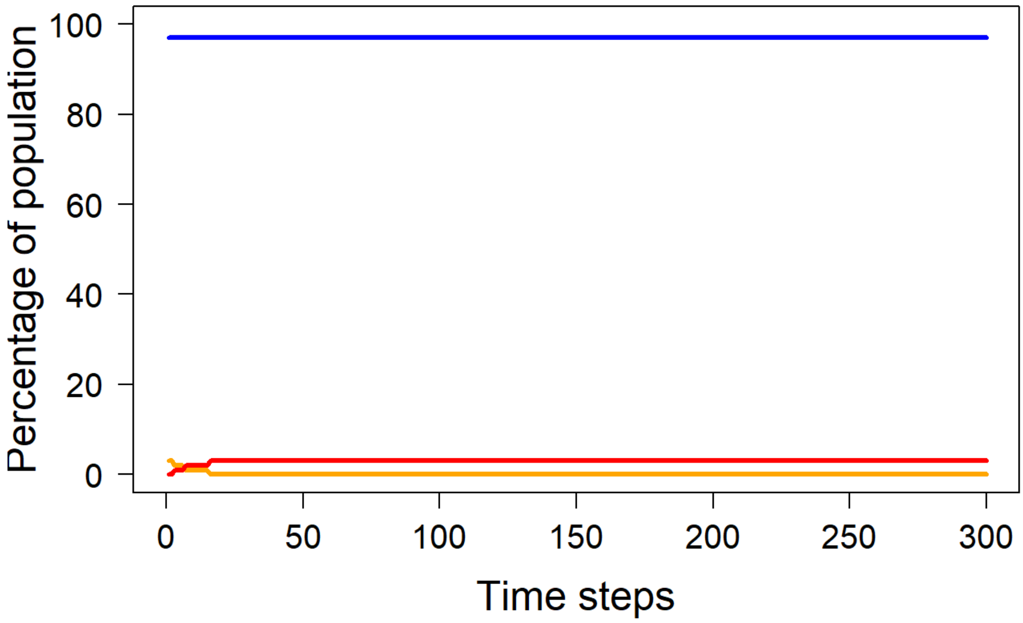

Percentage of population
Time steps

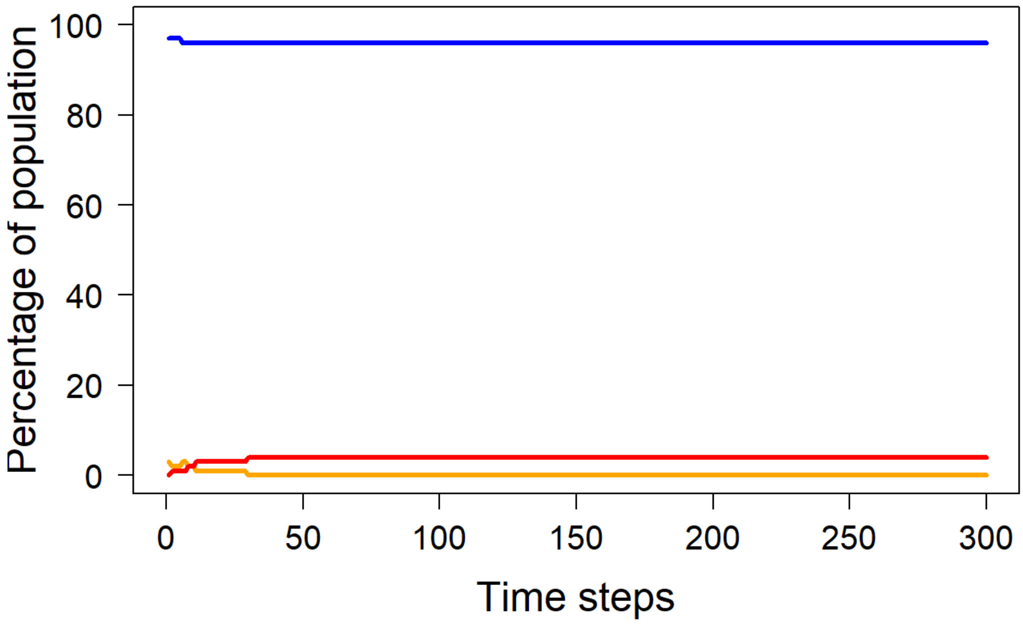

Percentage of population
Time steps

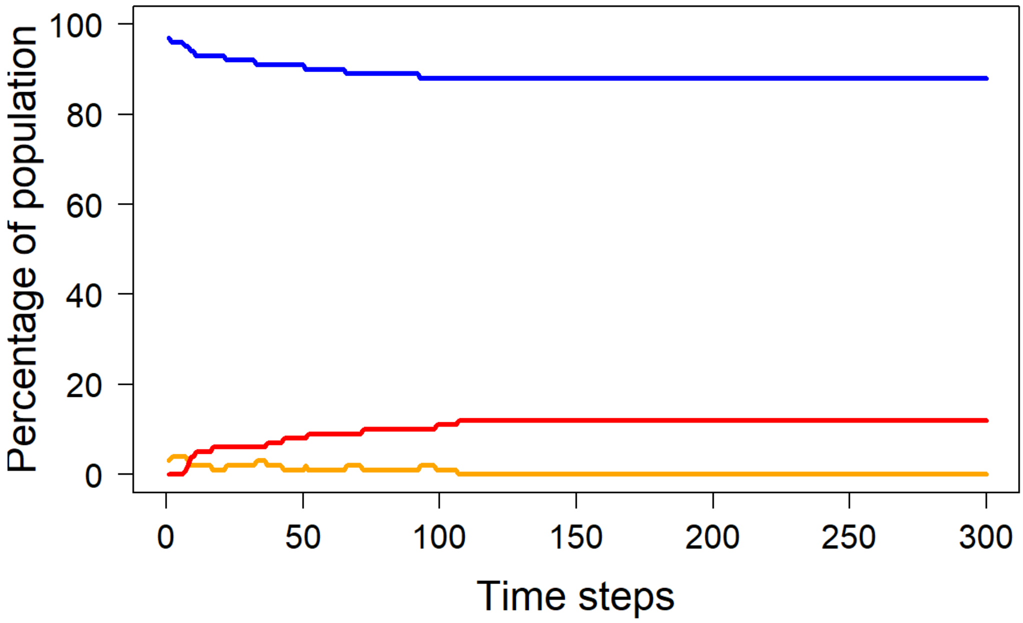

Percentage of population
100
80
60
40
20
0
0
50
100
150
200
250
300
Time steps

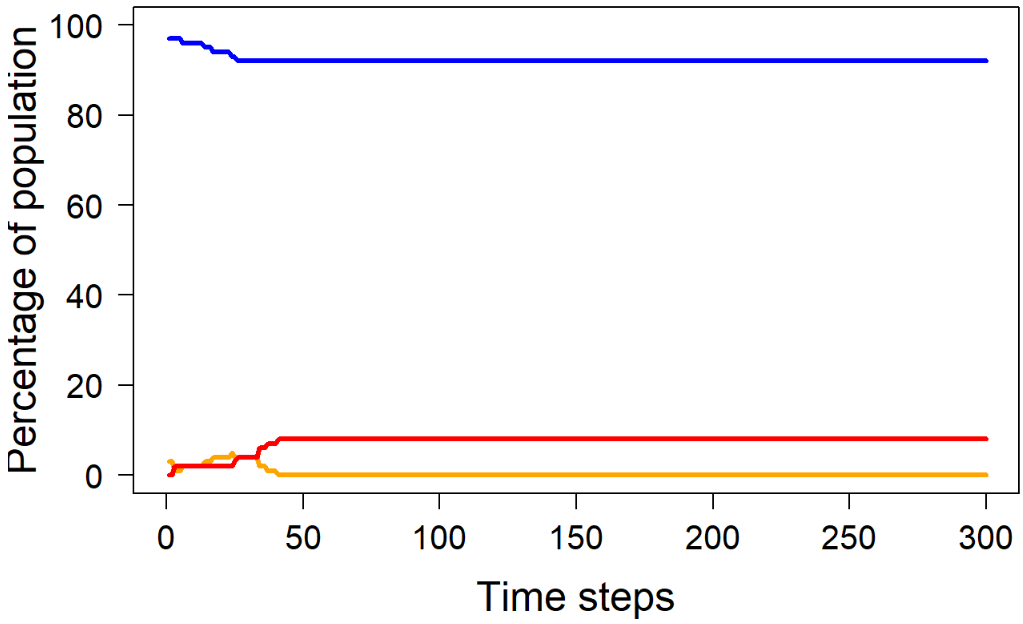

Percentage of population
100
80
60
40
20
0
0
50
100
150
200
250
300
Time steps

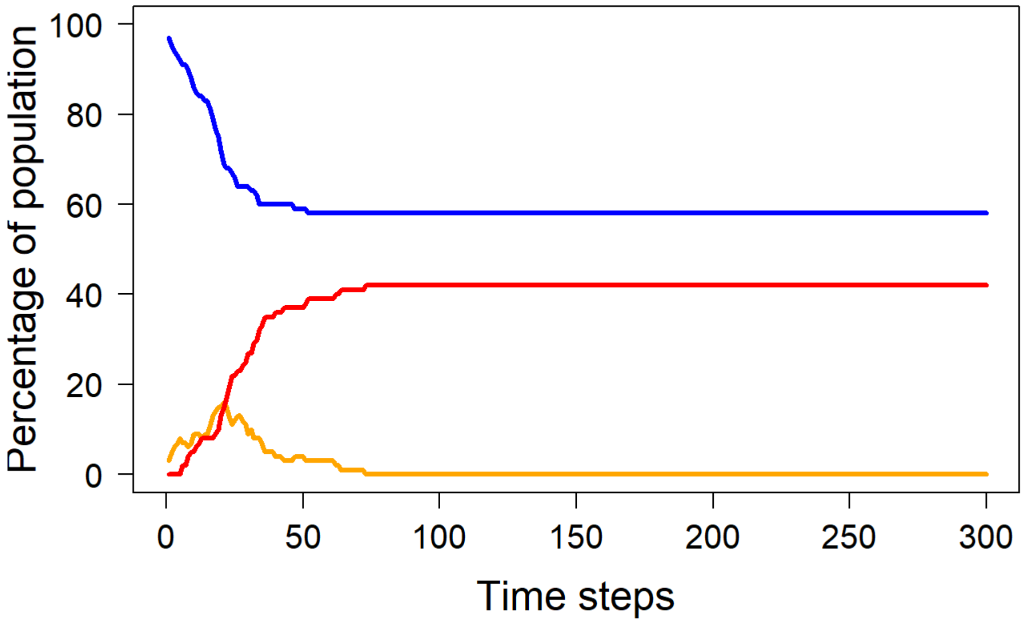

Percentage of population
100
80
60
40
20
0
0
50
100
150
200
250
300
Time steps

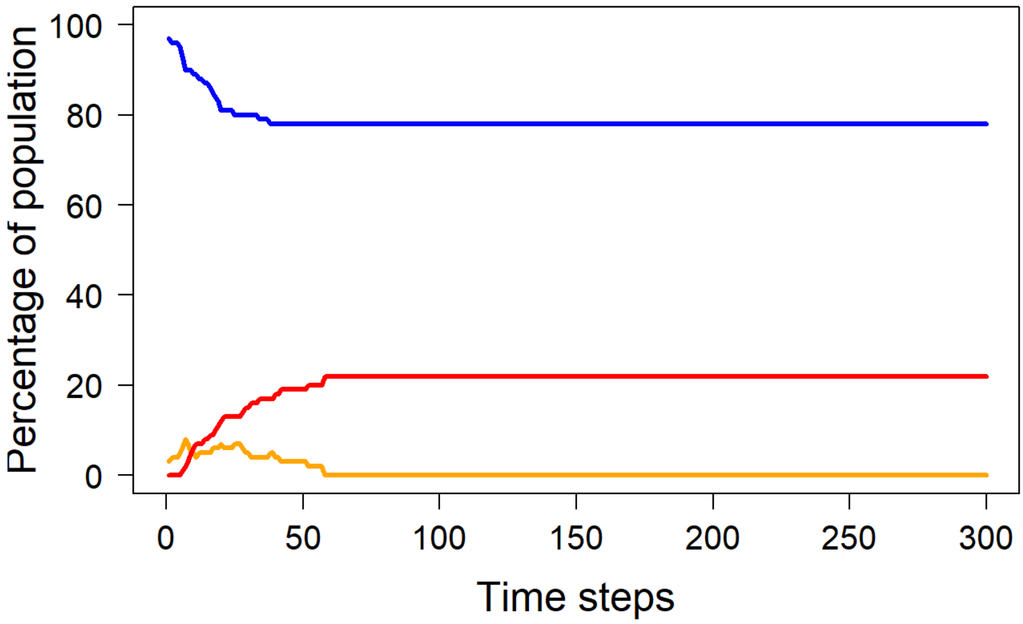

Percentage of population
100
80
60
40
20
0
0
50
100
150
200
250
300
Time steps

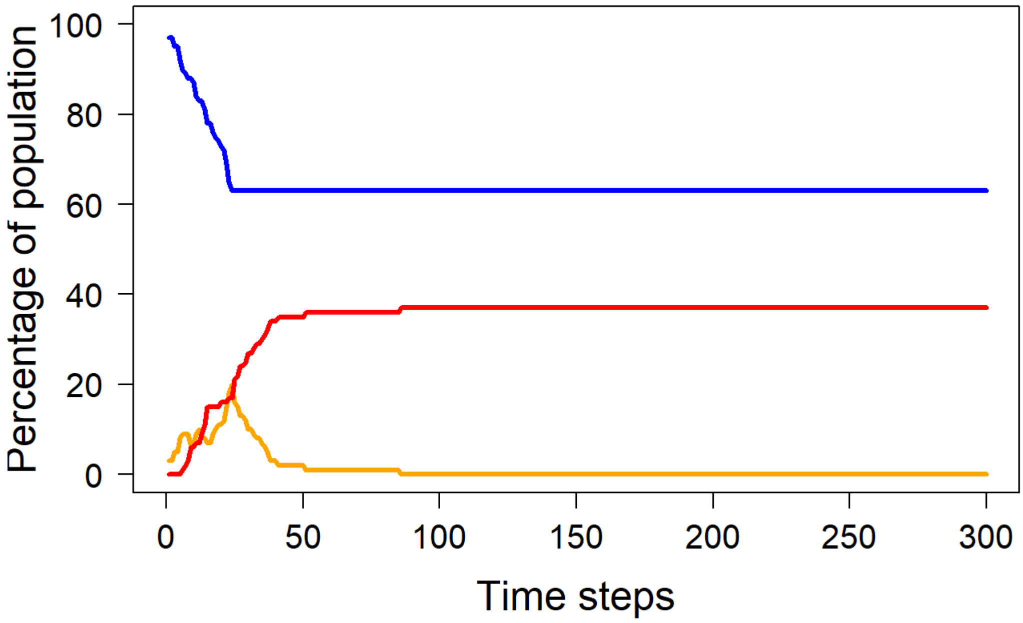
Percentage of population
0
20
40
60
80
100
0
50
100
150
200
250
300
Time steps

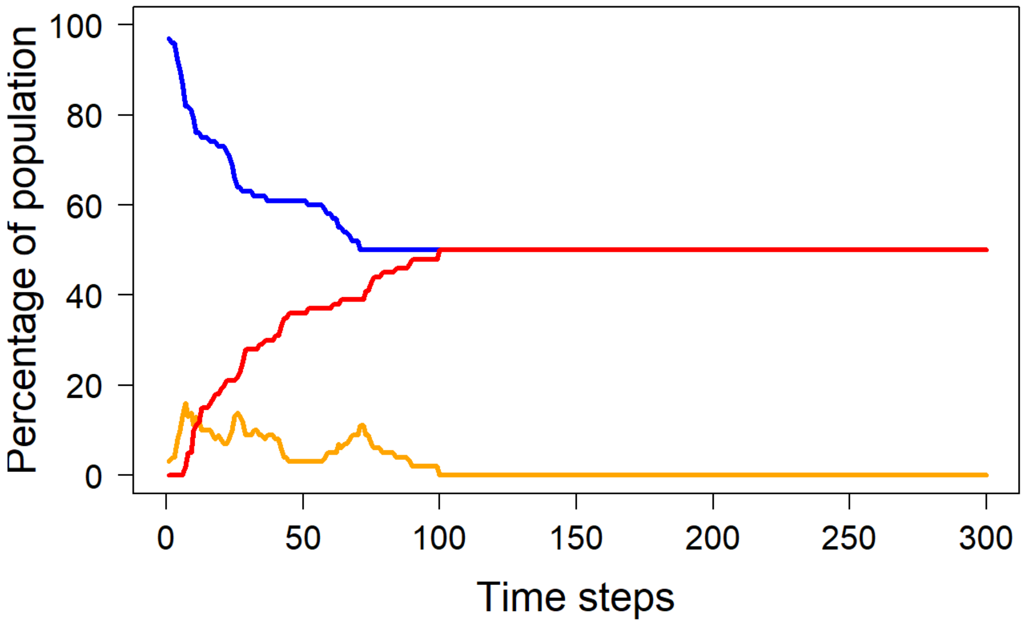
Percentage of population
0
20
40
60
80
100
0
50
100
150
200
250
300
Time steps

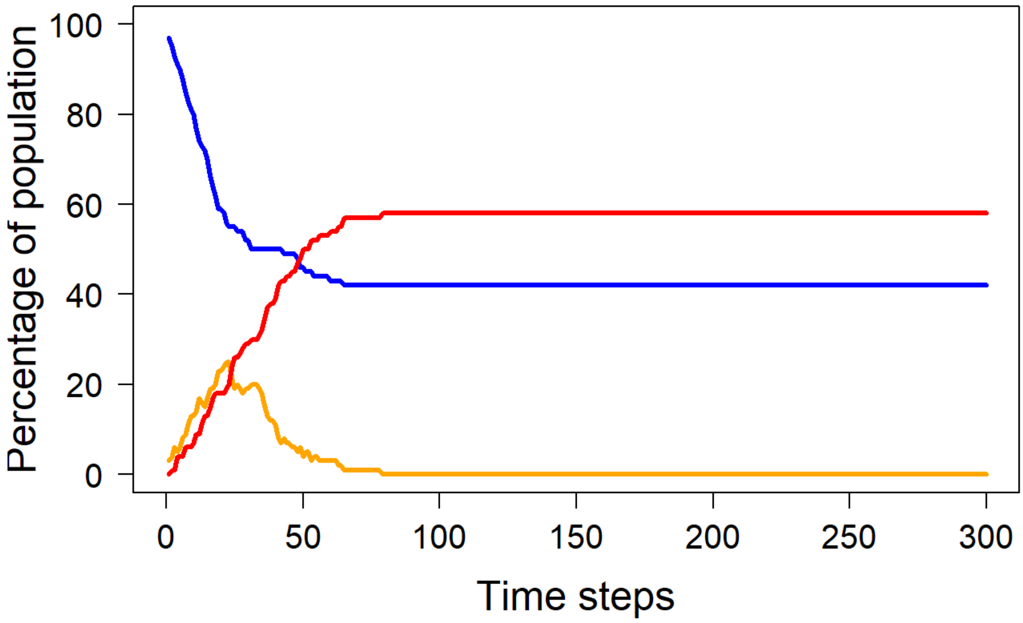
Percentage of population
0
20
40
60
80
100
0
50
100
150
200
250
300
Time steps

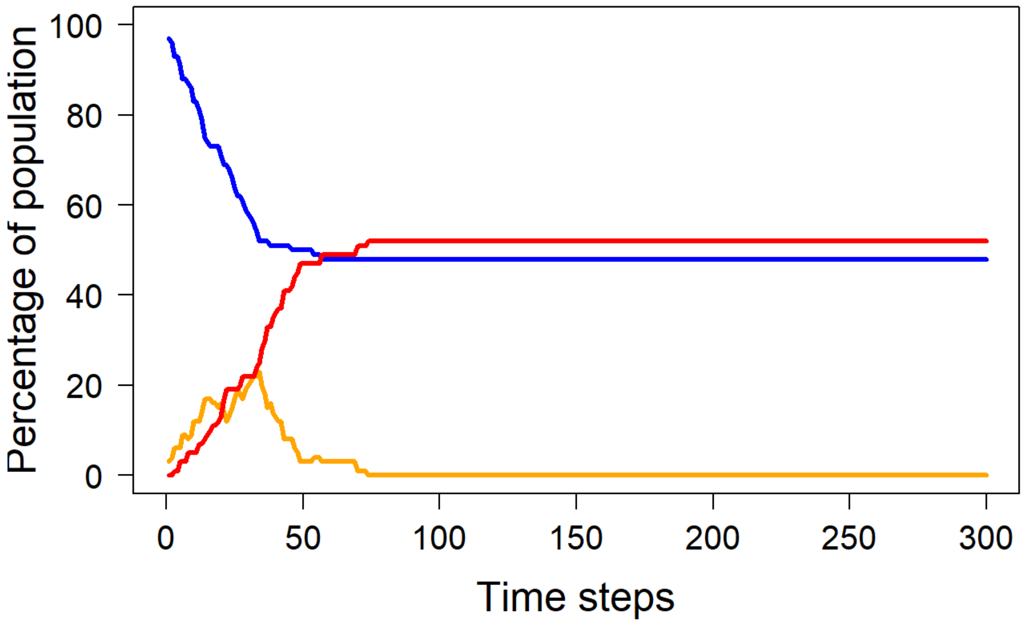
Percentage of population
0
20
40
60
80
100
0
50
100
150
200
250
300
Time steps

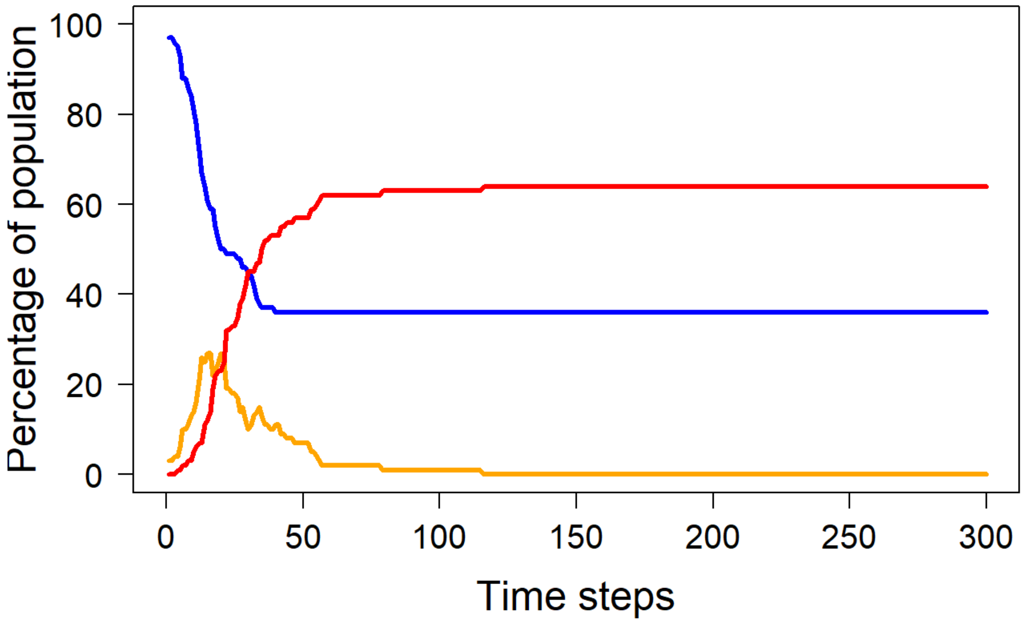
Percentage of population
0
20
40
60
80
100
0
50
100
150
200
250
300
Time steps

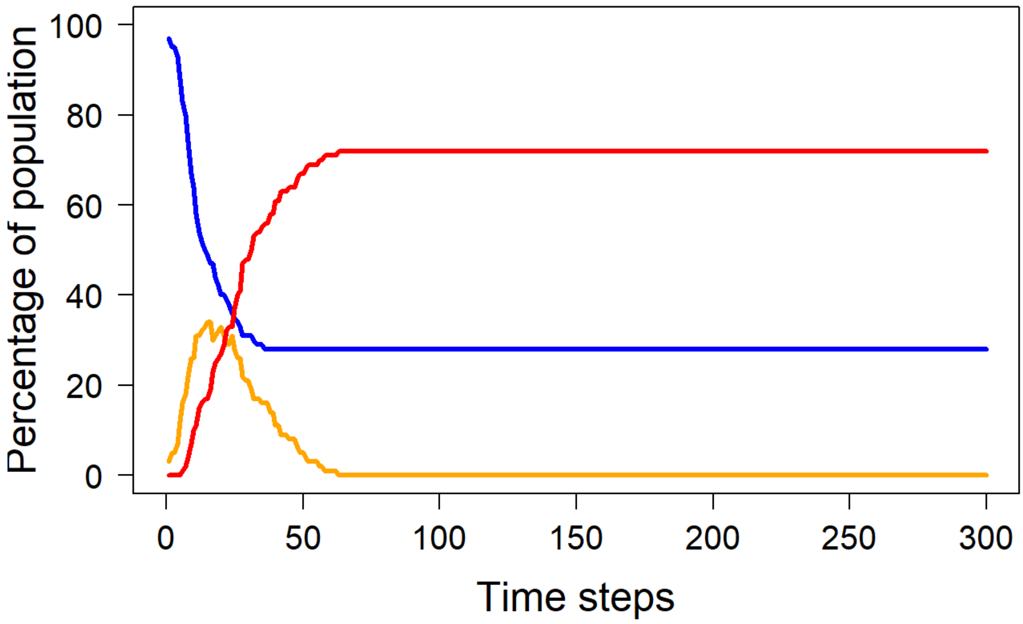
Percentage of population
0
20
40
60
80
100
0
50
100
150
200
250
300
Time steps

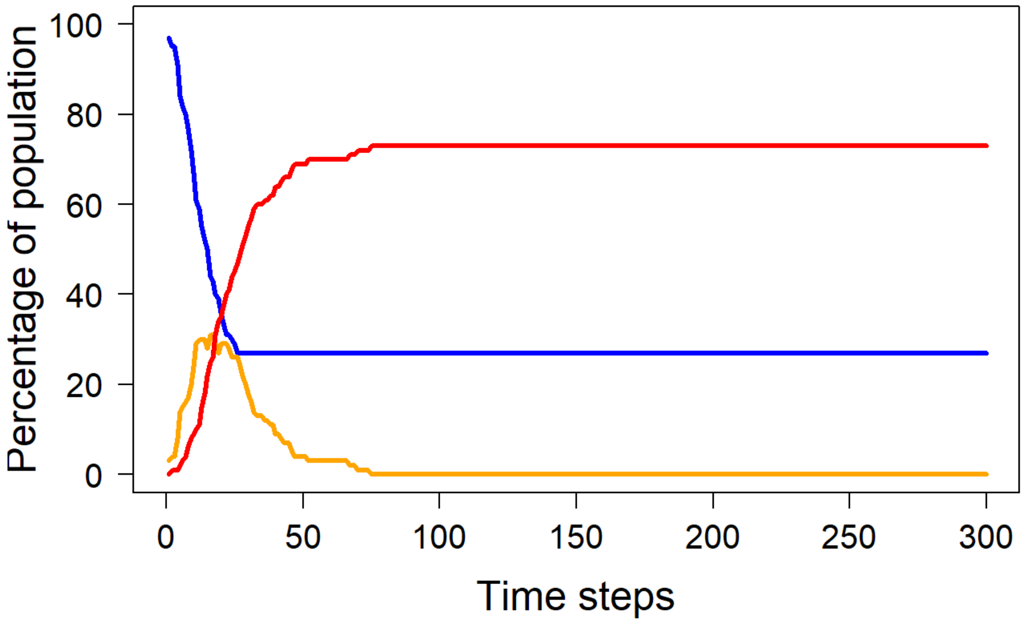

Percentage of population
Time steps

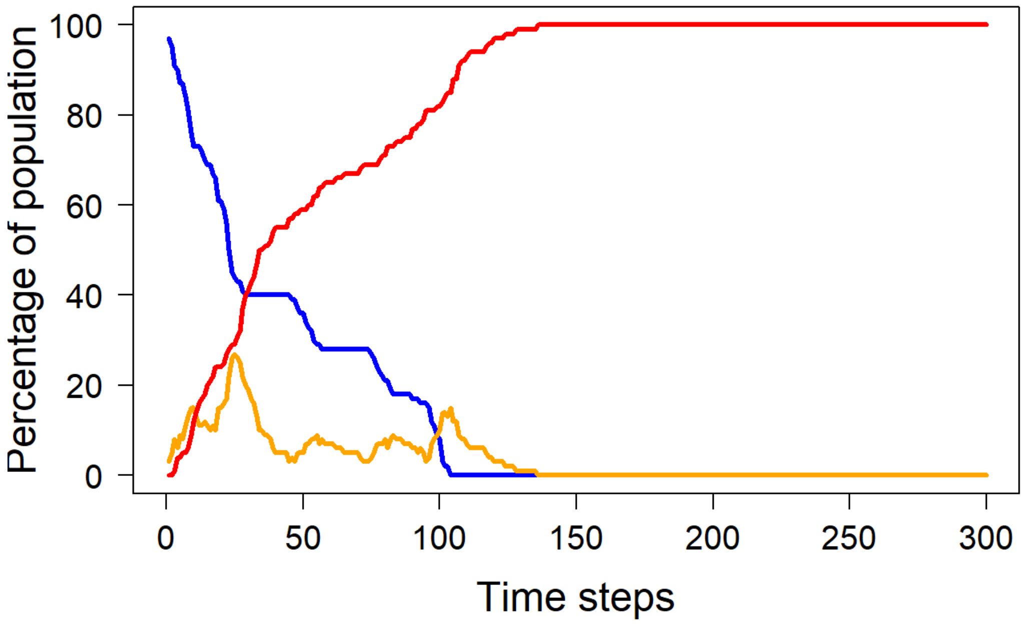

Percentage of population
Time steps

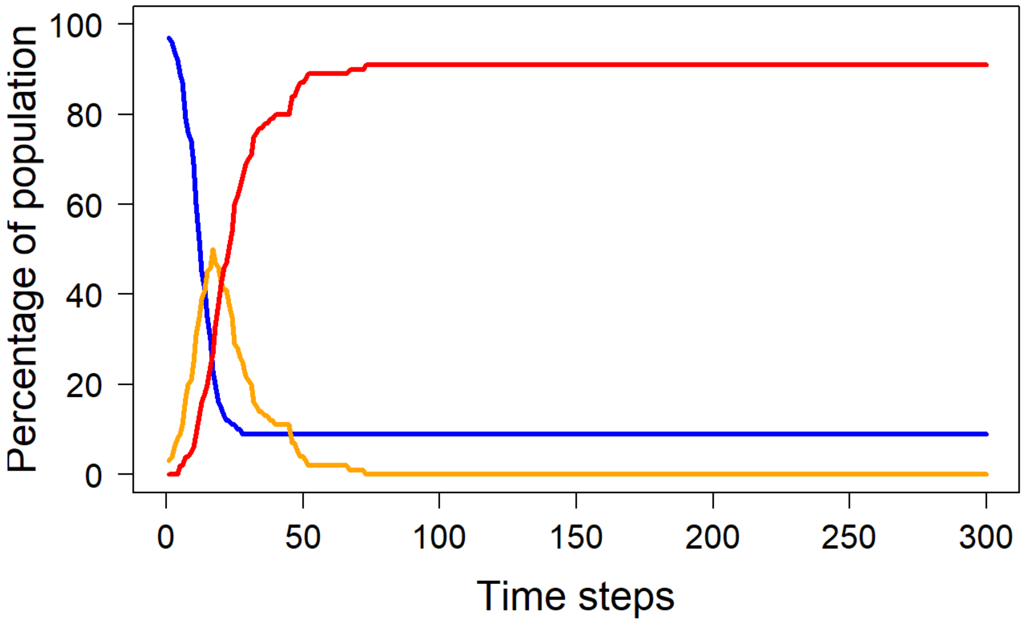

Percentage of population
100
80
60
40
20
0
0
50
100
150
200
250
300
Time steps

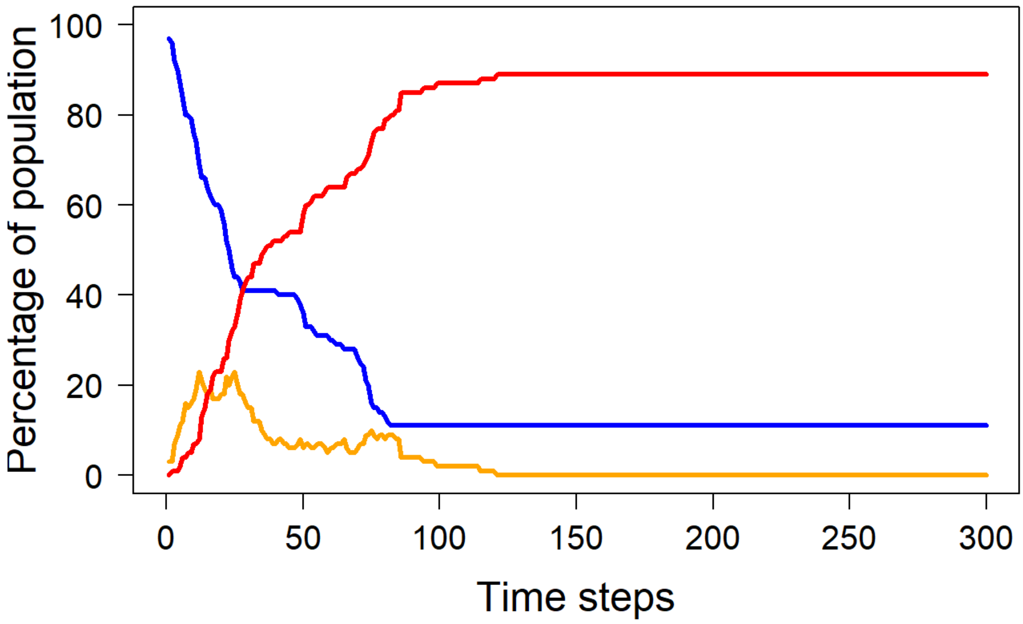

Percentage of population
100
80
60
40
20
0
0
50
100
150
200
250
300
Time steps

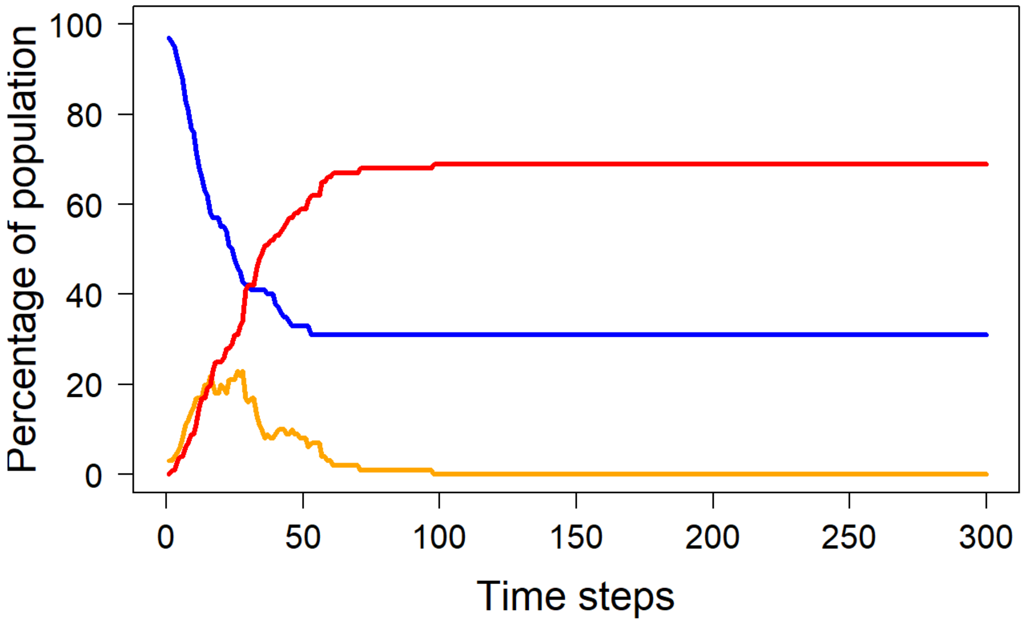
Percentage of population
0
20
40
60
80
100
0
50
100
150
200
250
300
Time steps

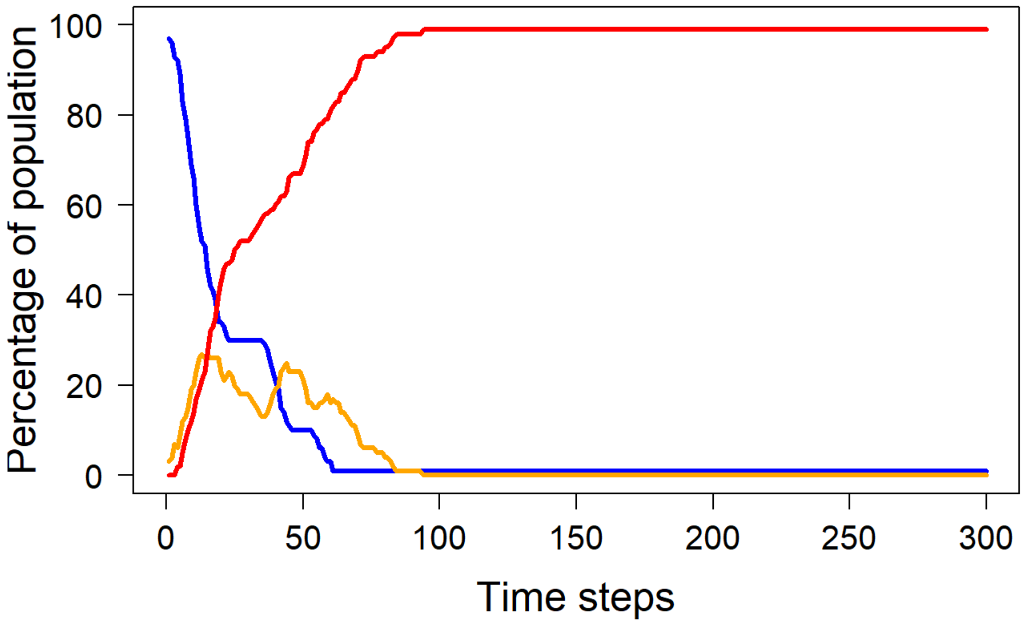
Percentage of population
0
20
40
60
80
100
0
50
100
150
200
250
300
Time steps

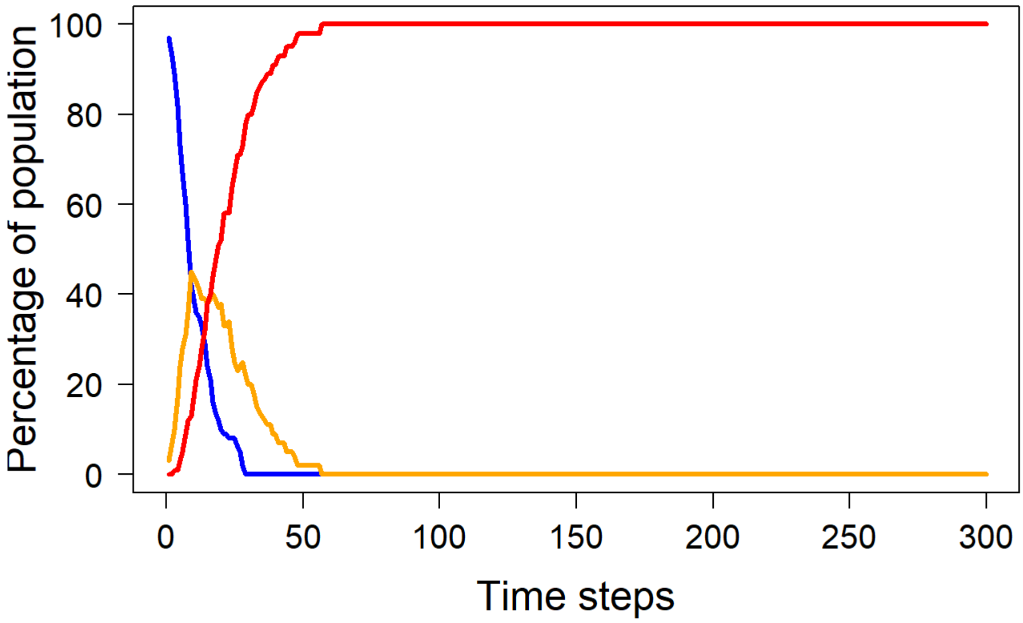
Percentage of population
0
20
40
60
80
100
0
50
100
150
200
250
300
Time steps

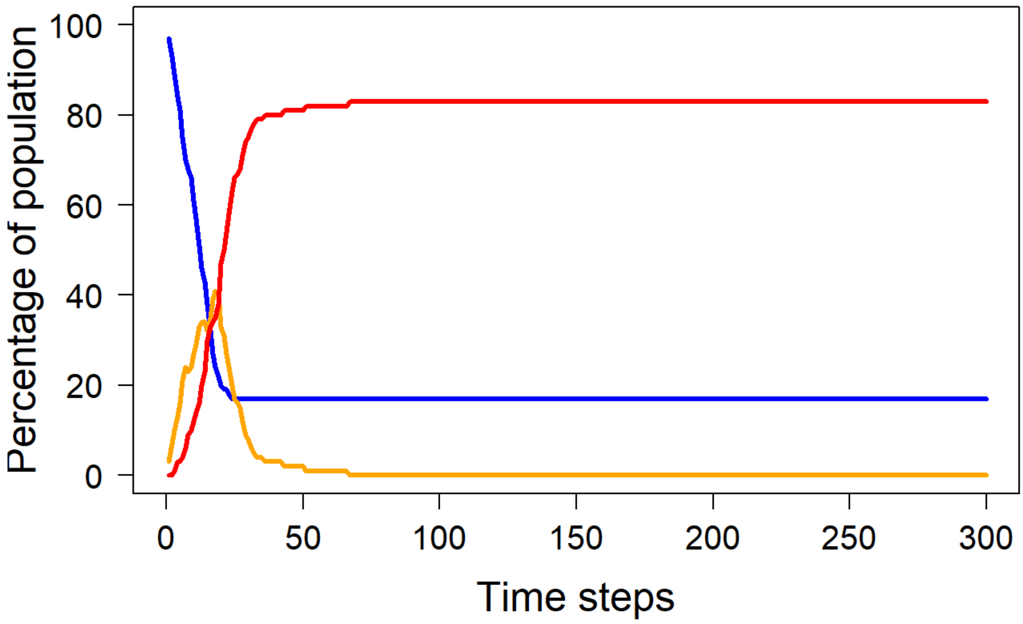
Percentage of population
0
20
40
60
80
100
0
50
100
150
200
250
300
Time steps

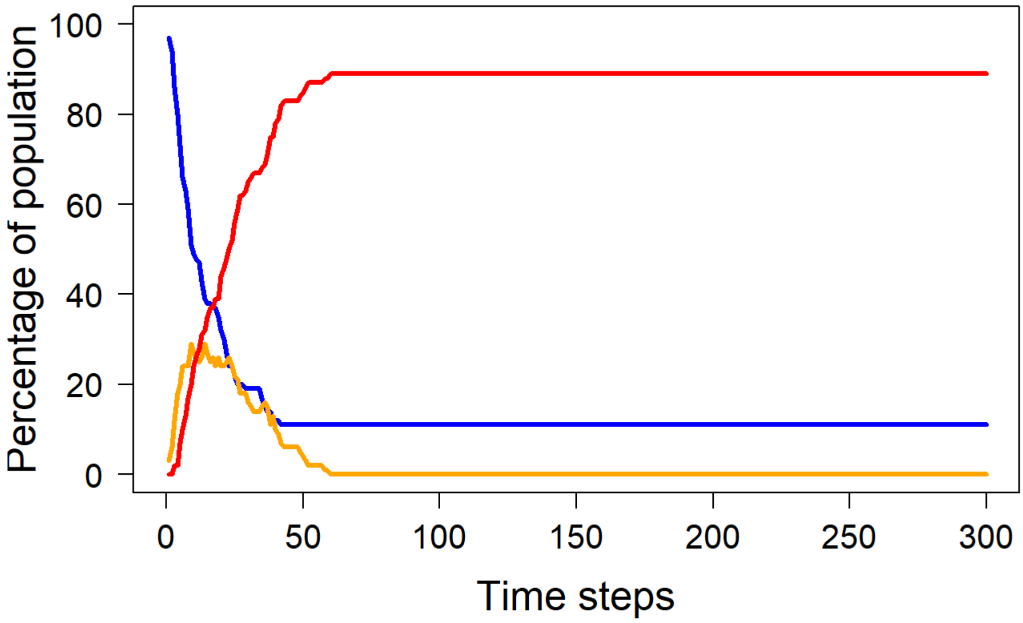


```
network1<-network
```

And now do the same for network B)

```
#Calculate density of network 1
#We will use this to guide the simulation of network 2
sum(network1)/length(network1)

#PROBABILITY OF WITHIN SUBPOPULATION CONTACTS
p.ig<-0.12

#PROBABILITY OF BETWEEN SUBPOPULATION CONTACTS
p.og<-0.1

#DISTANCE EFFECT ON BETWEEN SUBPOPULATION CONTACTS
dist.eff<- -0.0005 #plugged into exponential decay

#PROBABILITY OF SUCEPTIBLE INDIVIDUAL BEING INFECTED OVER AN EDGE PER TIME-STEP
S_I<-seq(0,0.1,0.005)

#PROBABILITY OF RECOVERY/DEATH PER TIME-STEP
I_R<-0.08
```

We now set up the population and its contact network. The population is 100 individuals, consisting of 10 subpopulations of 10 individuals.

```r
#create empty list to store outputs every time step
pop.info<-list()

n.groups<-10
s.groups<-10

#create initial population
tmp.p<-pop.gen(n.groups,s.groups,n.I=3)

#and store the output
pop.info[[1]]<-tmp.p[[1]]
group.locs<-tmp.p[[2]]

#create intitial network
network<-net.gen(pop=nrow(pop.info[[1]]),indiv.info=pop.info[[1]],p.ig,p.og,dist.eff,plot=T)

sum(network)/length(network)
```

```
## [1] 0.094
```

```
ovr.dat2<-list()
group.prevs2<-list()

for(ip in 1:length(S_I)){

pop.info[[1]]<-tmp.p[[1]]
group.locs<-tmp.p[[2]]

S_I2<-S_I[ip]

tmp.up<-ts(network=network,indiv.info=pop.info[[1]],n.groups=n.groups,group.locs=group.locs,S_I=S_I2,I_R=I_R
,plot=F)

pop.info[[2]]<-tmp.up[[1]]
network<-network

for(t in 3:300){
 tmp.up<-ts(network=network,indiv.info=tmp.up[[1]],n.groups=n.groups,group.locs=group.locs,S_I=S_I2,I_R=I_R,
plot=F)
 pop.info[[t]]<-tmp.up[[1]]
}

summaries<-lapply(pop.info,colSums)

sus<-numeric()
inf<-numeric()
rec<-numeric()

for(i in 1:300){
 sus[i]<-summaries[[i]][5]
 inf[i]<-summaries[[i]][6]
 rec[i]<-summaries[[i]][7]
}

plot(sus,xlim=c(0,300),ylim=c(0,100),type="l",col="blue",lwd=3,ylab="Percentage of population",xlab="Time st
eps",cex.lab=1.6,cex.axis=1.3,las=1)
lines(inf,col="orange",lwd=3)
lines(rec,col="red",lwd=3)

ovr.dat2[[ip]]<-data.frame(sus,inf,rec)

group.prev<-matrix(NA,nr=300,nc=10)

for(i in seq(1,300,1)){
 tmp<-aggregate(pop.info[[i]][,6],list(pop.info[[i]]$Group),mean)
 for(j in 1:nrow(tmp)){
  group.prev[i,as.numeric(as.vector(tmp)[j,1])]<-as.vector(tmp)[j,2]
 }
}

group.prevs2[[ip]]<-group.prev

}
```

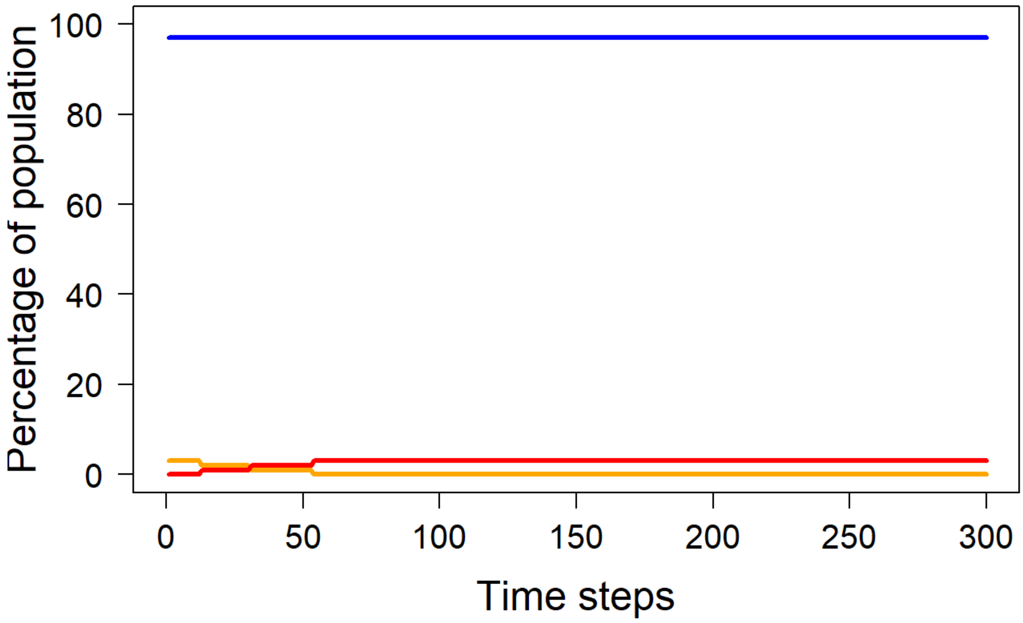

Percentage of population
100
80
60
40
20
0
0
50
100
150
200
250
300
Time steps

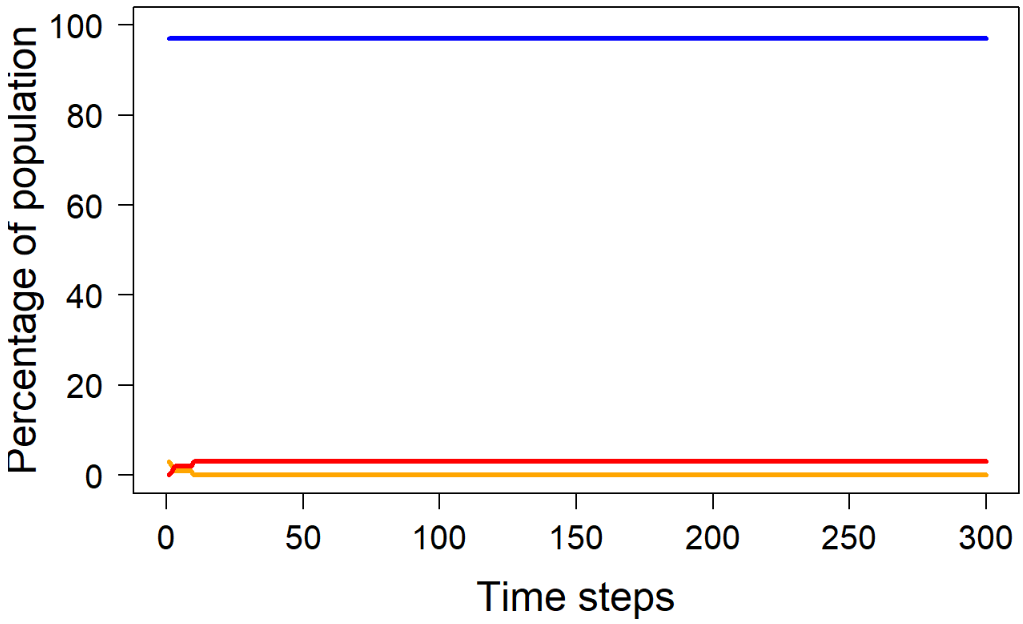

Percentage of population
100
80
60
40
20
0
0
50
100
150
200
250
300
Time steps

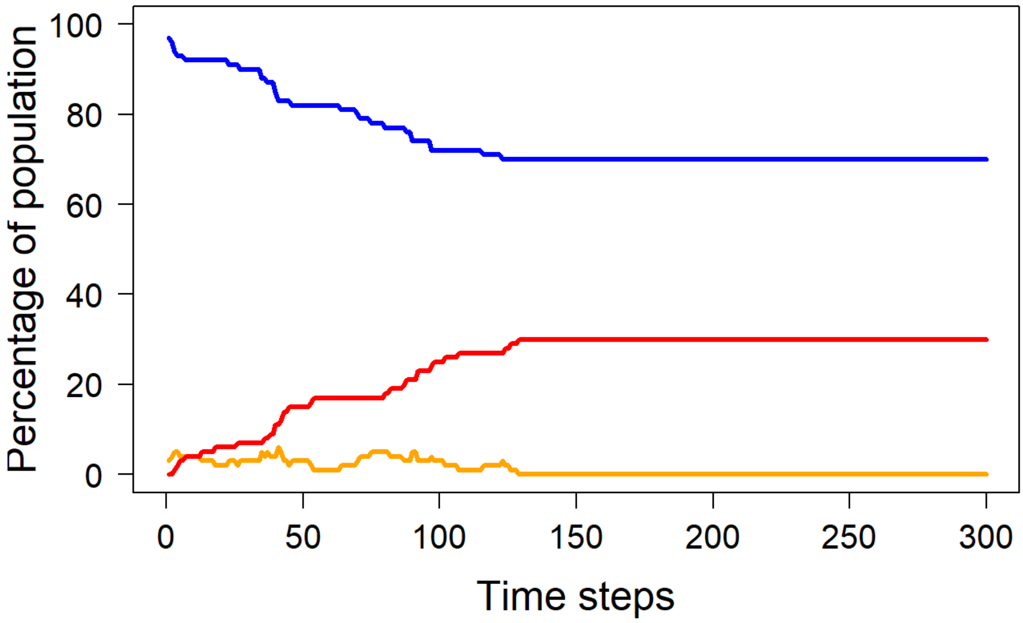
Percentage of population
100
80
60
40
20
0
0
50
100
150
200
250
300
Time steps

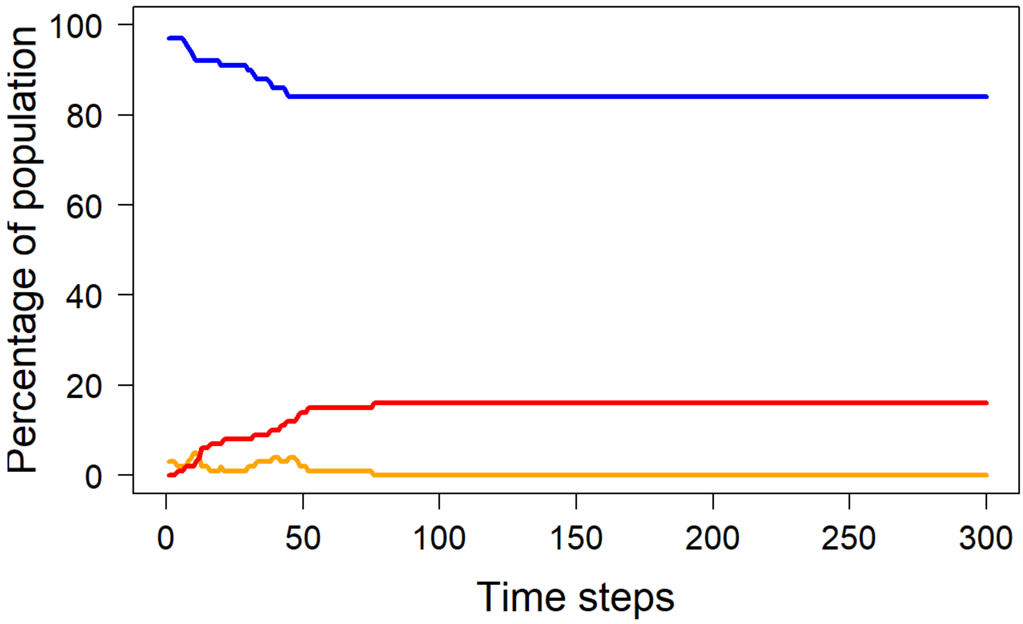
Percentage of population
100
80
60
40
20
0
0
50
100
150
200
250
300
Time steps

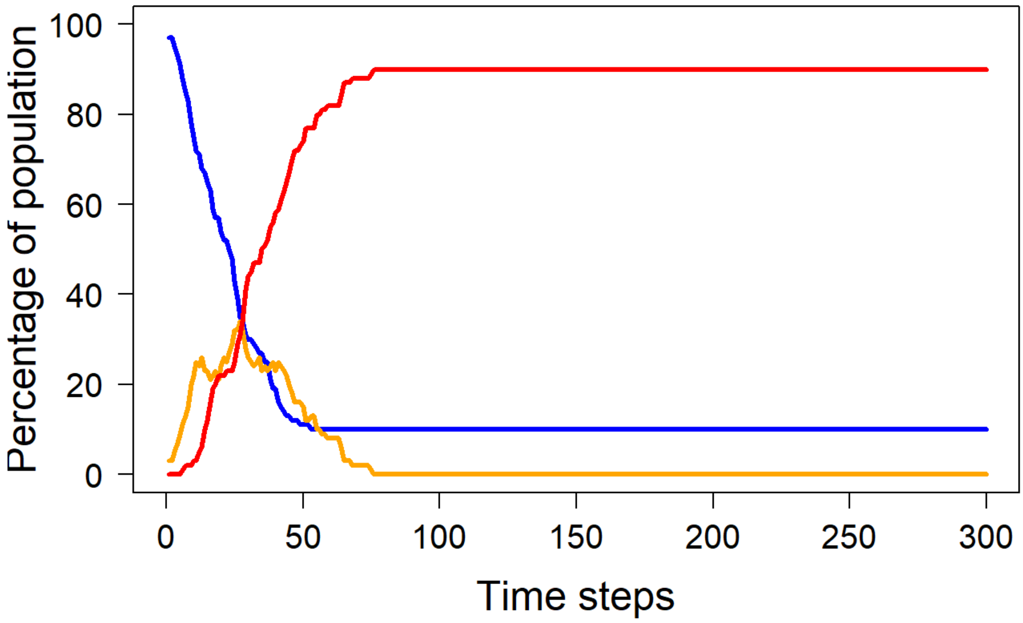
Percentage of population
100
80
60
40
20
0
0
50
100
150
200
250
300
Time steps

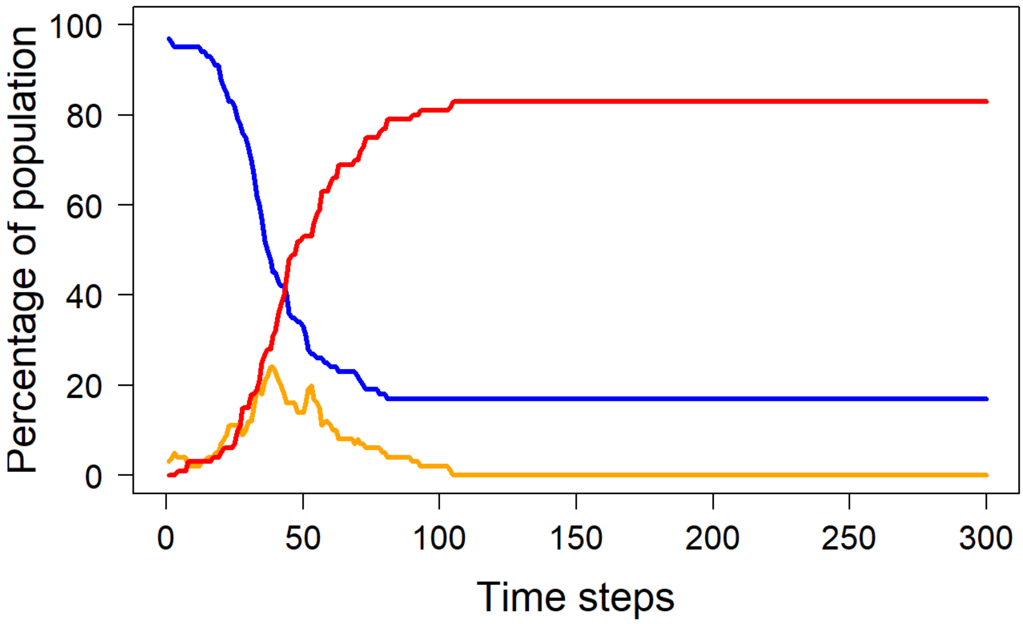
Percentage of population
100
80
60
40
20
0
0
50
100
150
200
250
300
Time steps

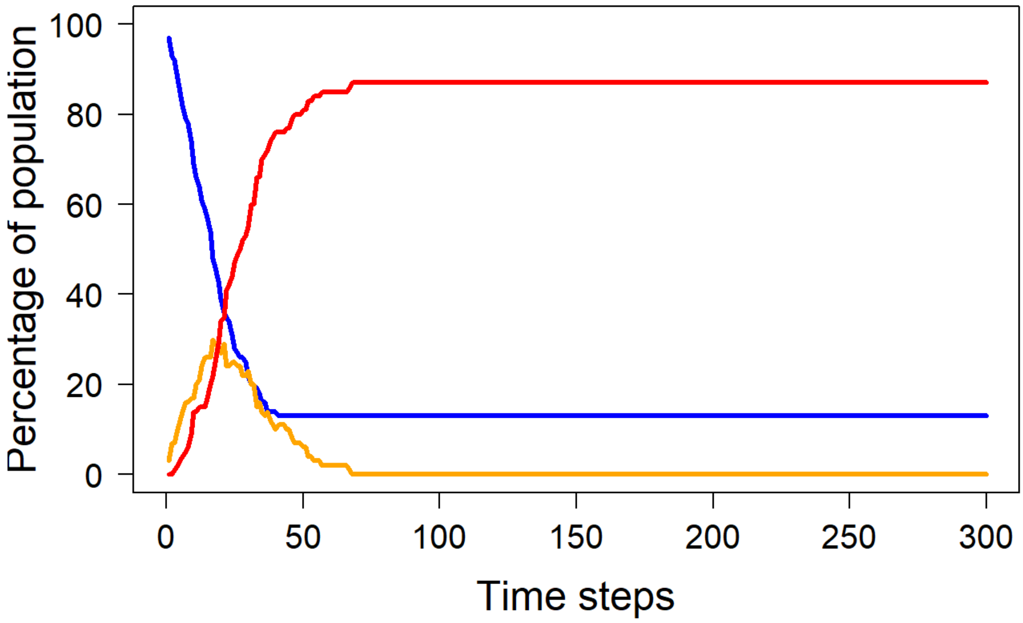
Percentage of population
100
80
60
40
20
0
0
50
100
150
200
250
300
Time steps

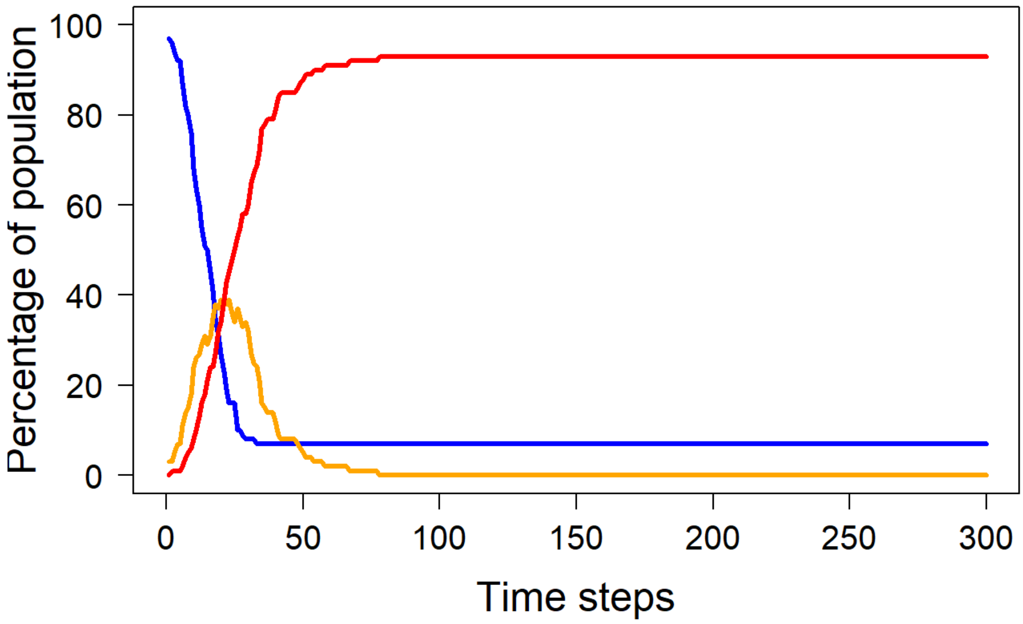
Percentage of population
100
80
60
40
20
0
0
50
100
150
200
250
300
Time steps

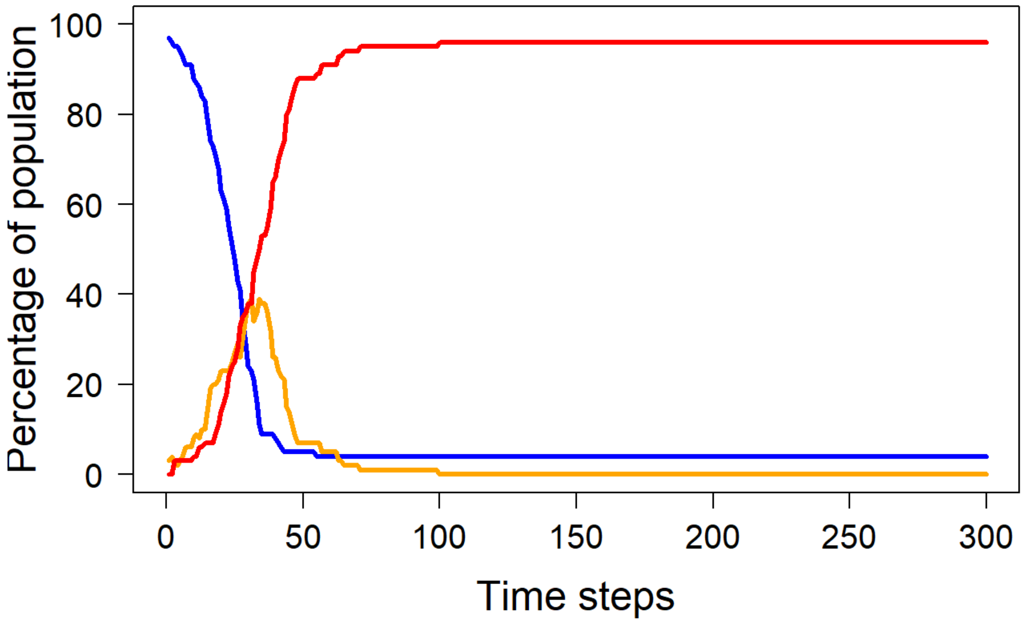
Percentage of population
0
20
40
60
80
100
0
50
100
150
200
250
300
Time steps

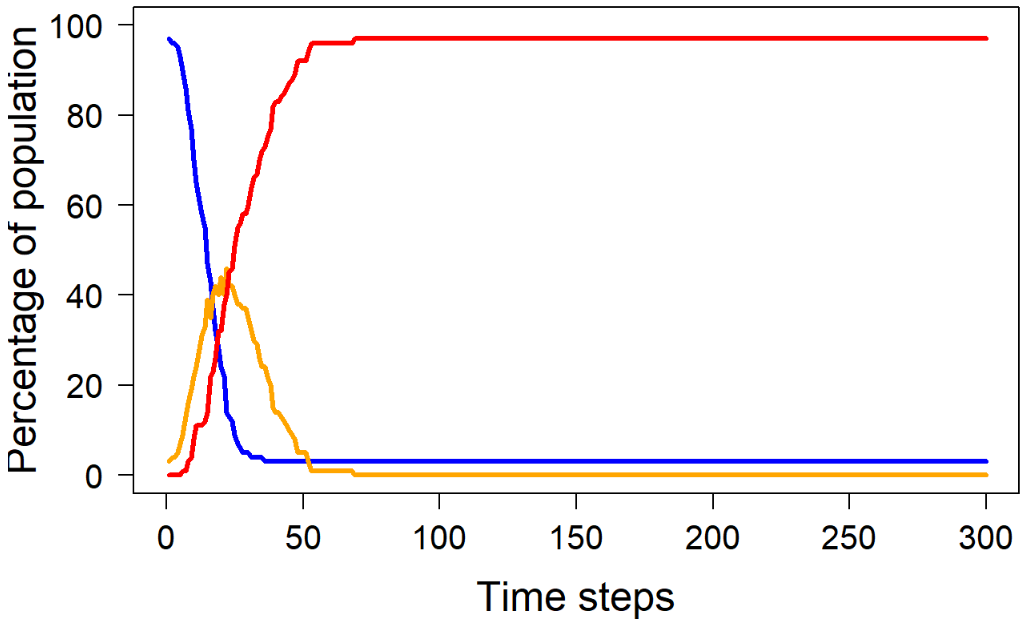
Percentage of population
0
20
40
60
80
100
0
50
100
150
200
250
300
Time steps

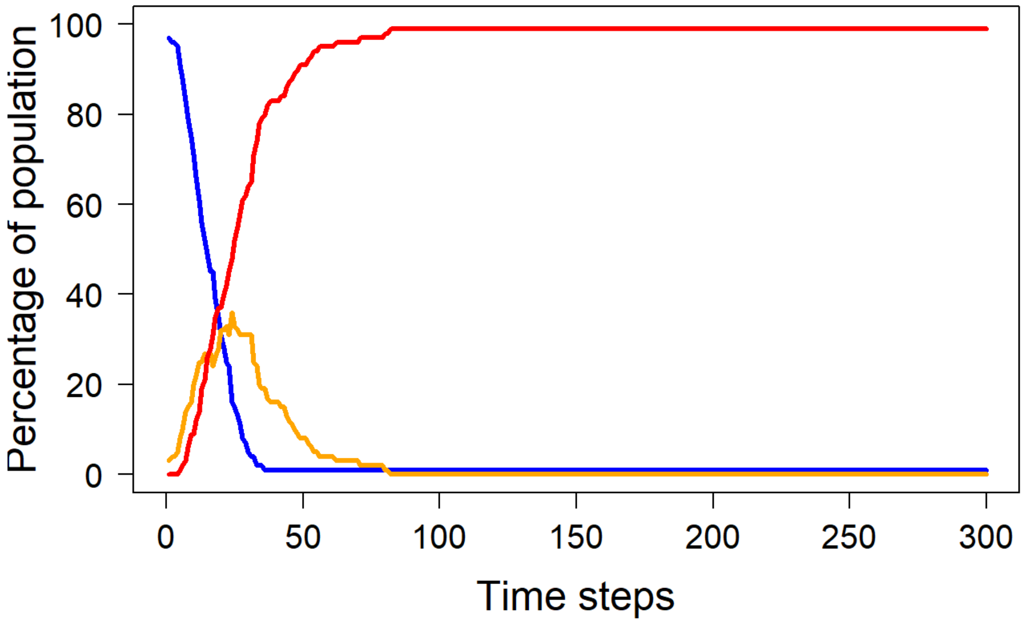
Percentage of population
100
80
60
40
20
0
0
50
100
150
200
250
300
Time steps

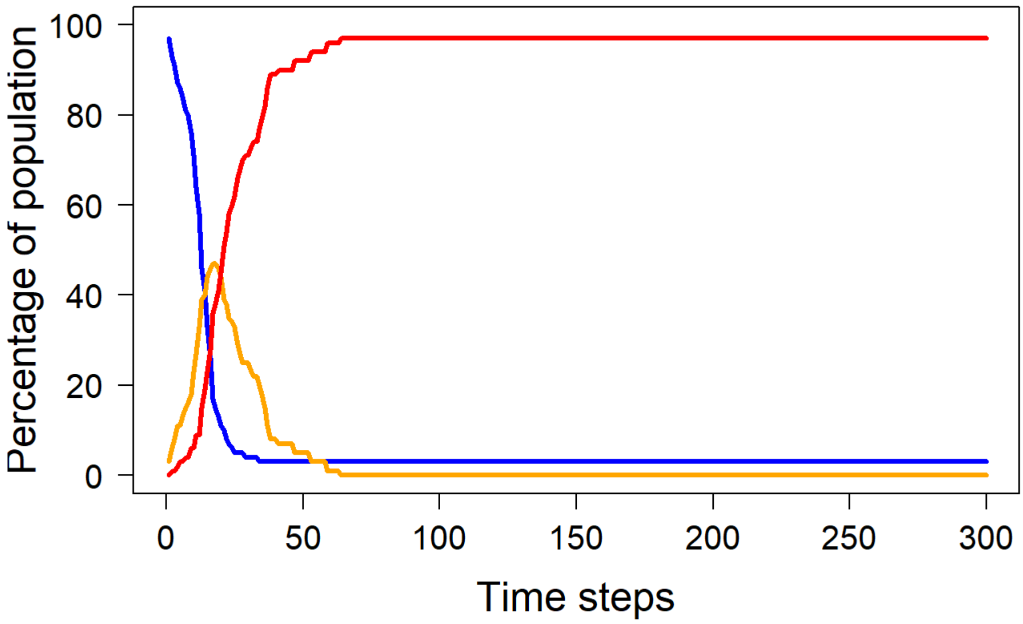
Percentage of population
100
80
60
40
20
0
0
50
100
150
200
250
300
Time steps

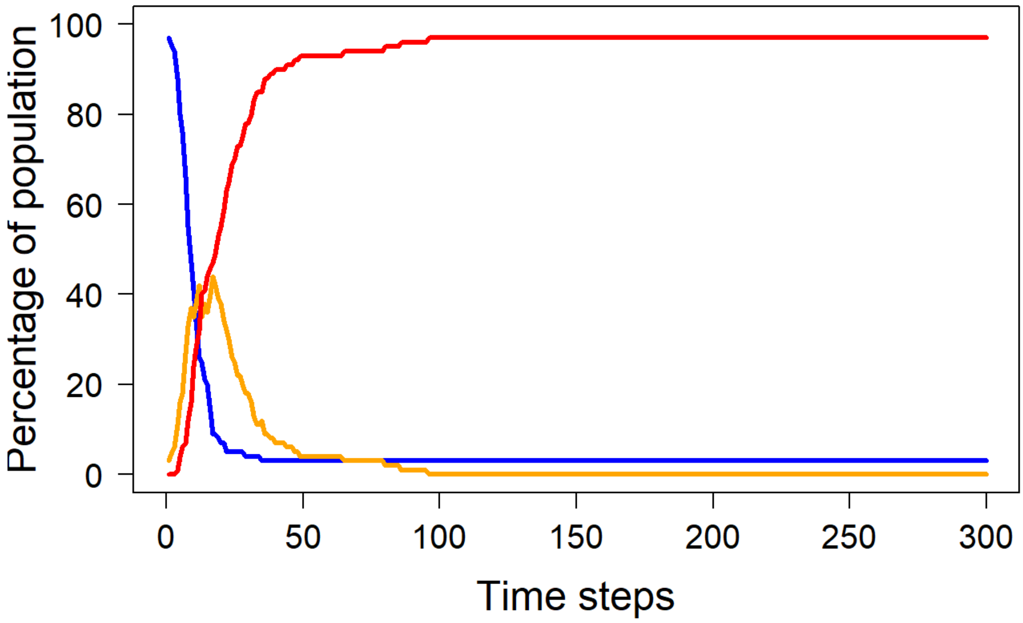

Percentage of population
0
20
40
60
80
100
0
50
100
150
200
250
300
Time steps

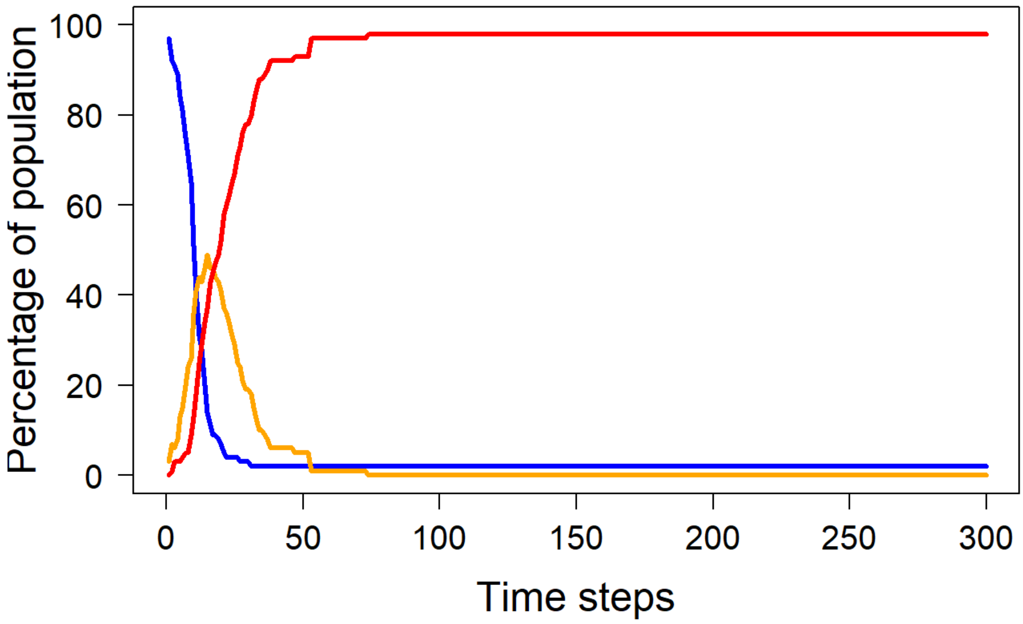

Percentage of population
0
20
40
60
80
100
0
50
100
150
200
250
300
Time steps

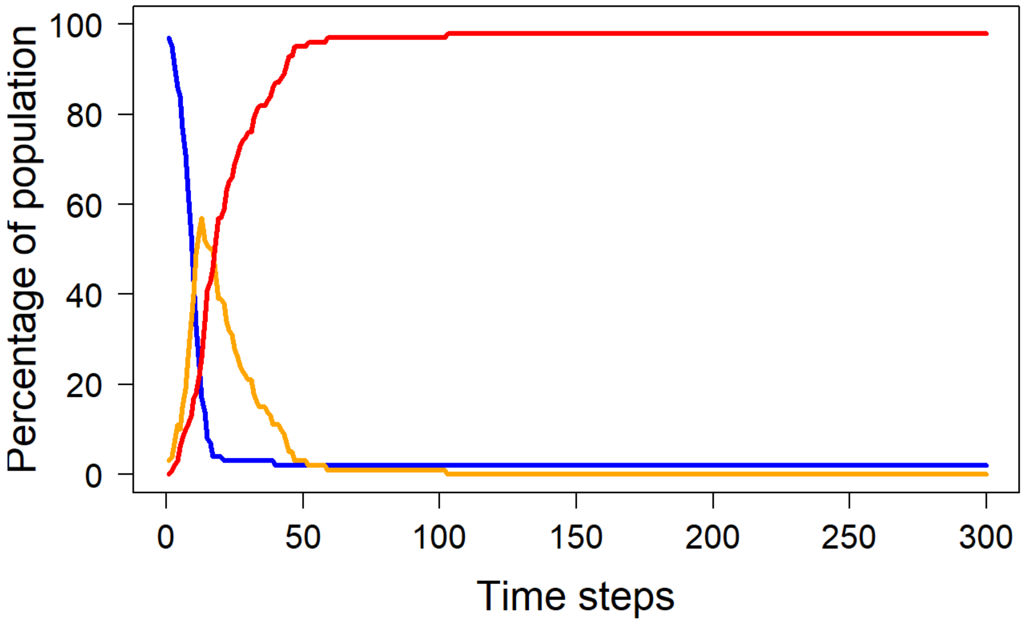
Percentage of population
100
80
60
40
20
0
0
50
100
150
200
250
300
Time steps

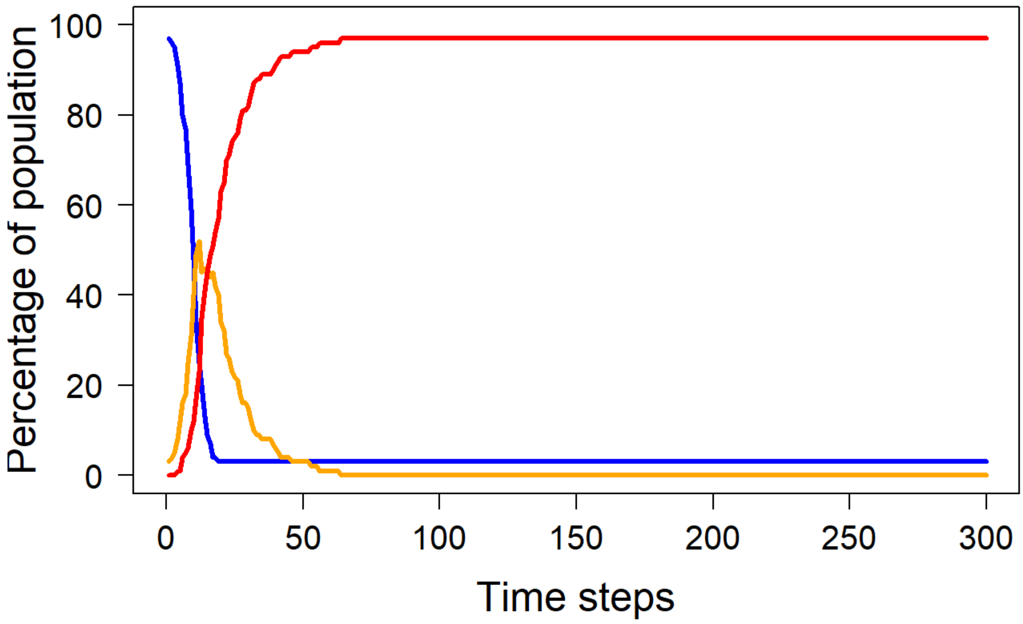
Percentage of population
100
80
60
40
20
0
0
50
100
150
200
250
300
Time steps

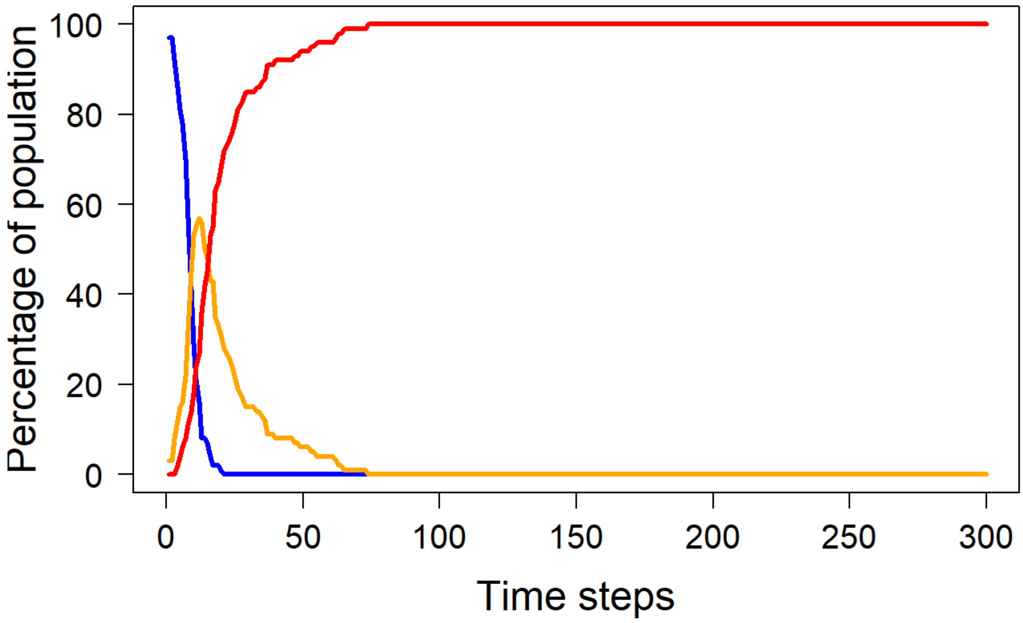
Percentage of population
0
20
40
60
80
100
0
50
100
150
200
250
300
Time steps

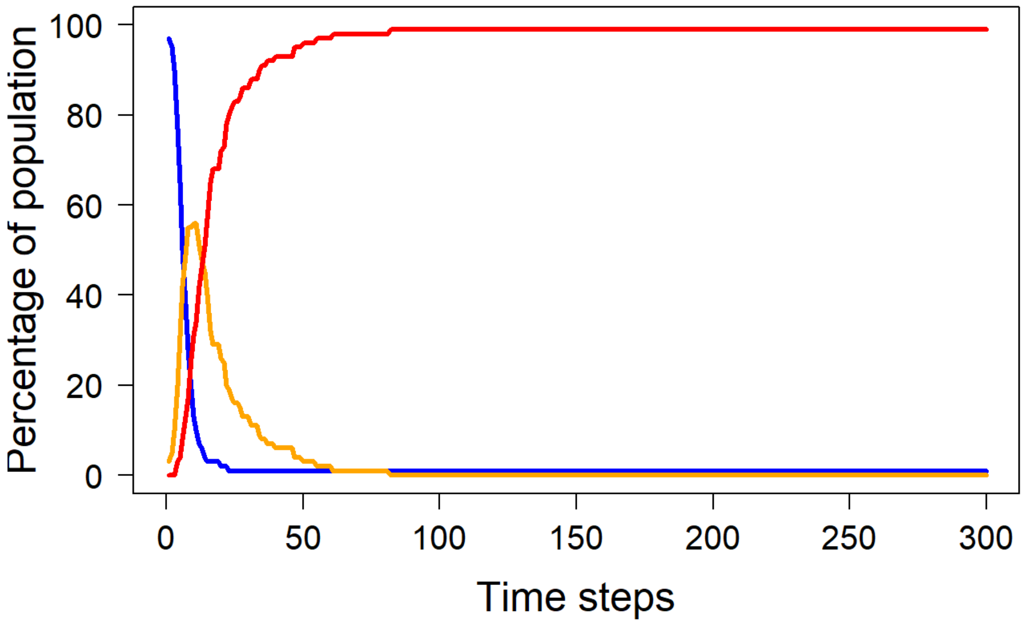
Percentage of population
0
20
40
60
80
100
0
50
100
150
200
250
300
Time steps

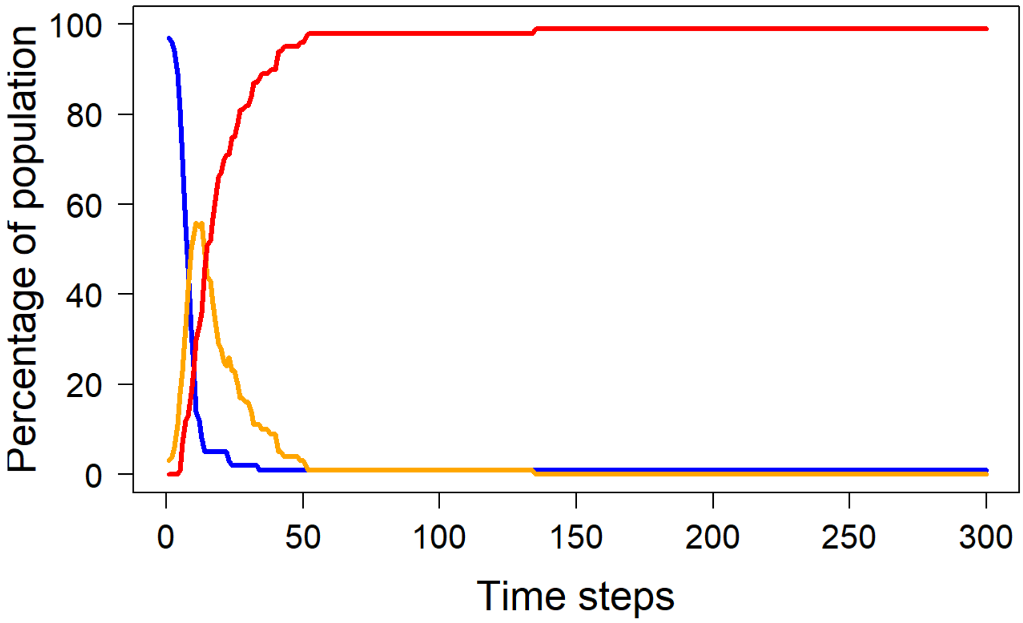
Percentage of population
0
20
40
60
80
100
0
50
100
150
200
250
300
Time steps

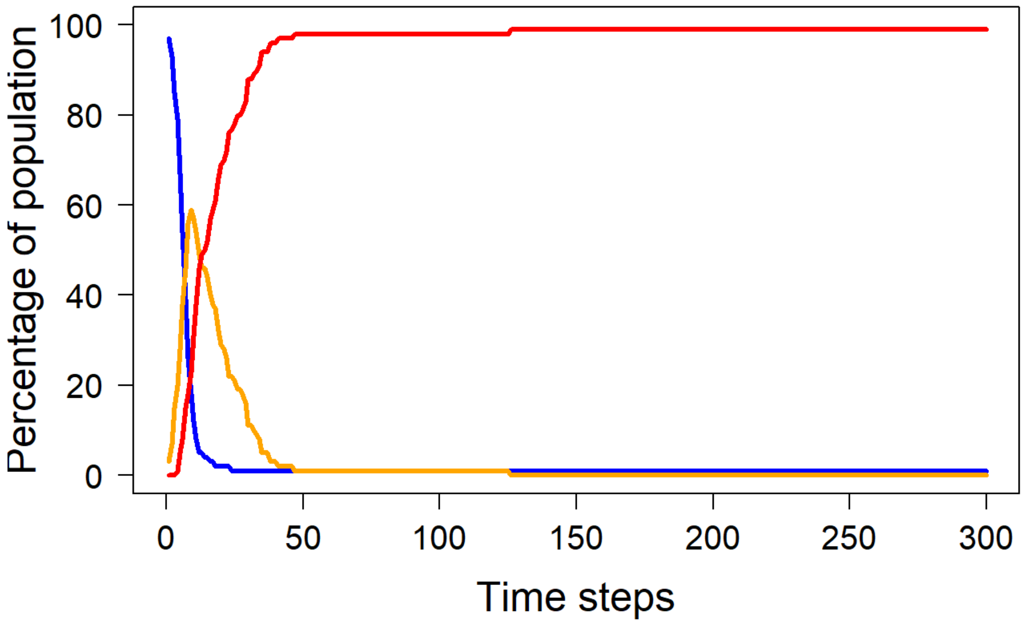
Percentage of population
0
20
40
60
80
100
0
50
100
150
200
250
300
Time steps

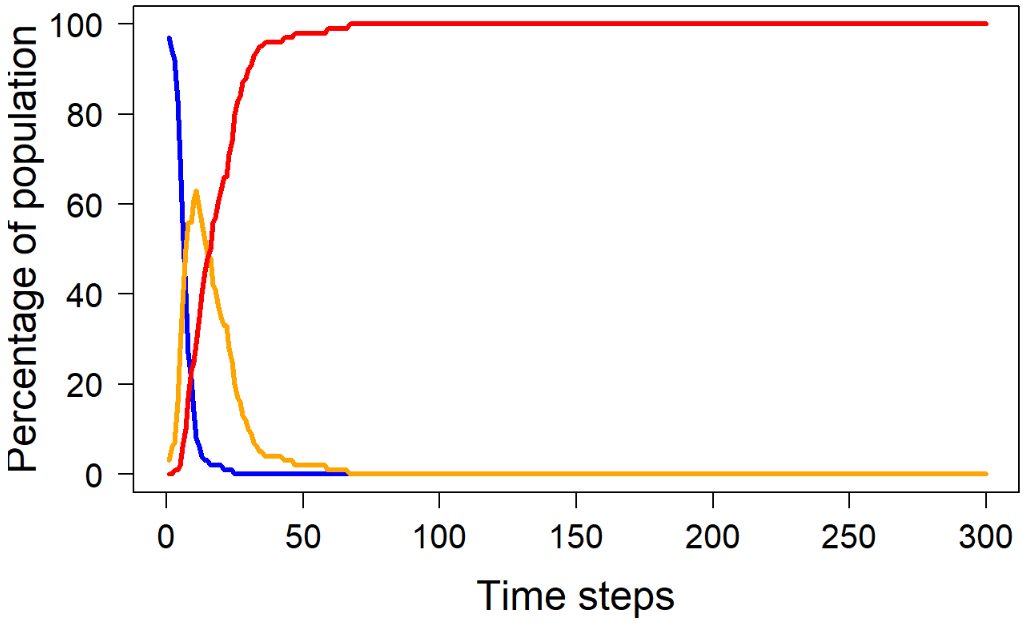


```
RES1<-unlist(lapply(ovr.dat,'[',300,1))
RES2<-unlist(lapply(ovr.dat2,'[',300,1))

peak.inf<-function(a){
  b<-min(which(a[,2]==max(a[,2])))
  return(b)
}

PEAK1<-unlist(lapply(ovr.dat,peak.inf))
PEAK2<-unlist(lapply(ovr.dat2,peak.inf))

par(mfrow=c(1,2))
plot(1-RES1/100~S_I,ylim=c(0,1),pch=16,ylab="Proportion of population dead",xlab="Transmission Probability")
plot(1-RES2/100~S_I,ylim=c(0,1),pch=16,ylab="Proportion of population dead",xlab="Transmission Probability")
```

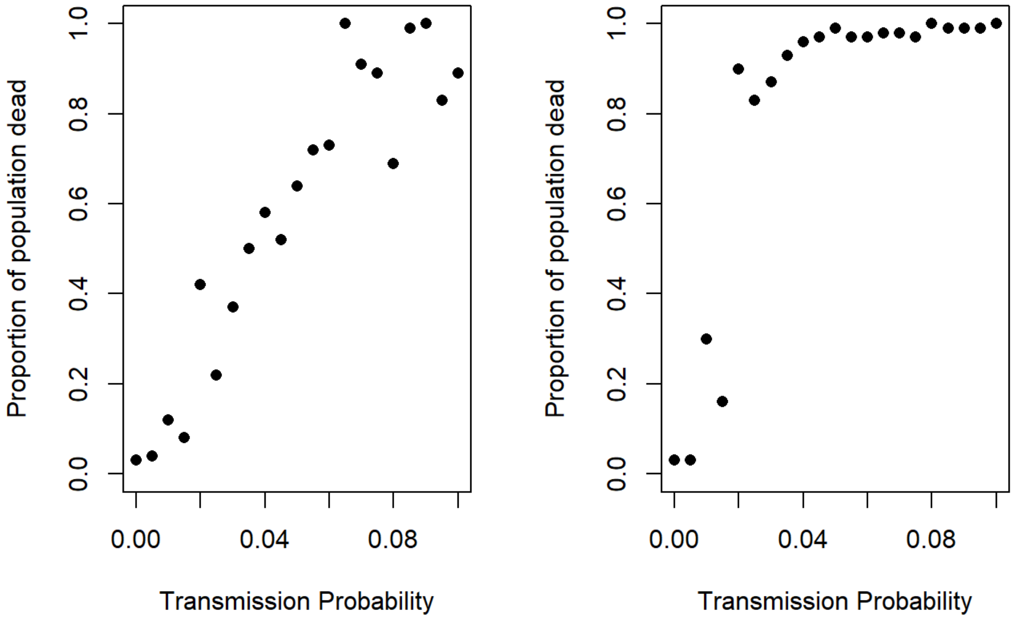

```
par(mfrow=c(1,2))
plot(1-RES1/100~S_I,ylim=c(0,1),pch=15,ylab="Proportion of population dead",xlab="Transmission Probability",
col="firebrick")
points(y=1-RES2/100, x=S_I,ylim=c(0,1),pch=19,ylab="Proportion of population dead",xlab="Transmission Probab
ility",col="dark blue")
plot(PEAK1~S_I,ylim=c(0,60),pch=15,ylab="Time-step of peak outbreak size",xlab="Transmission Probability",co
l="firebrick")
points(y=PEAK2, x=S_I,ylim=c(0,60),pch=19,ylab="Time-step of peak outbreak size",xlab="Transmission Probabil
ity",col="dark blue")
```

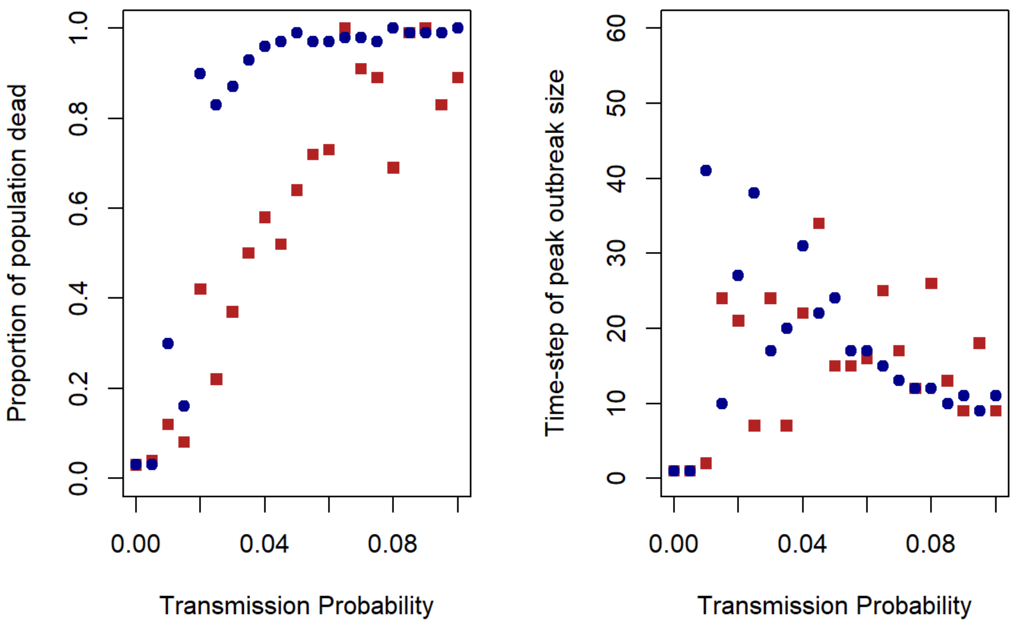


```
layout.n<-layout.auto(graph.adjacency(network1,mode="undirected"))

par(mfrow=c(2,2))
plot(graph.adjacency(network1,mode="undirected"),
     vertex.color="grey",
     layout=layout.n,vertex.label=NA,vertex.size=8)
plot(graph.adjacency(network,mode="undirected"),
     vertex.color="grey",
     layout=layout.n,vertex.label=NA,vertex.size=8)
plot(1-RES1/100~S_I,ylim=c(0,1),pch=15,ylab="Proportion of population dead",xlab="Transmission Probability",
col="firebrick")
points(y=1-RES2/100, x=S_I,ylim=c(0,1),pch=19,ylab="Proportion of population dead",xlab="Transmission Probab
ility",col="dark blue")
plot(PEAK1~S_I,ylim=c(0,60),pch=15,ylab="Time-step of peak outbreak size",xlab="Transmission Probability",co
l="firebrick")
points(y=PEAK2, x=S_I,ylim=c(0,60),pch=19,ylab="Time-step of peak outbreak size",xlab="Transmission Probabil
ity",col="dark blue")
```

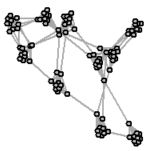

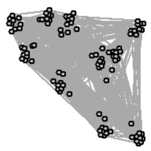

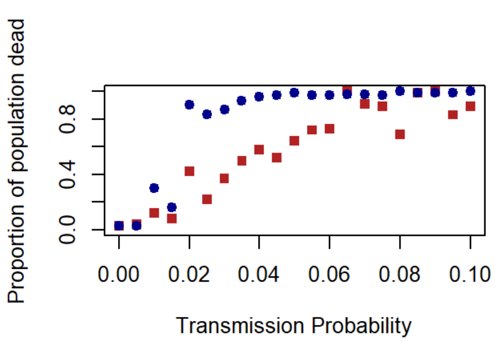

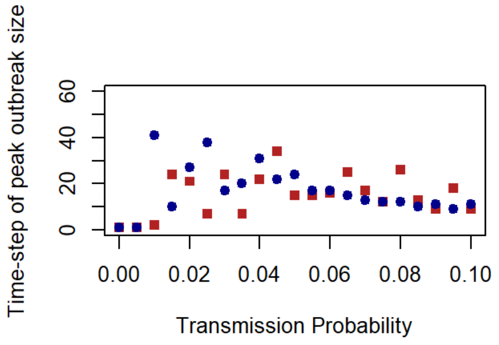


```
par(mfrow=c(1,1),mar=c(5, 6, 4, 2))
plot((RES1/100)*100~S_I,ylim=c(0,100),pch=15,ylab="Size of surviving population",xlab="Transmission Probabil
ity",col="firebrick",las=1,cex.lab=1.6,cex.axis=1.4)
points(y=(RES2/100)*100, x=S_I,ylim=c(0,1),pch=19,col="dark blue")
```

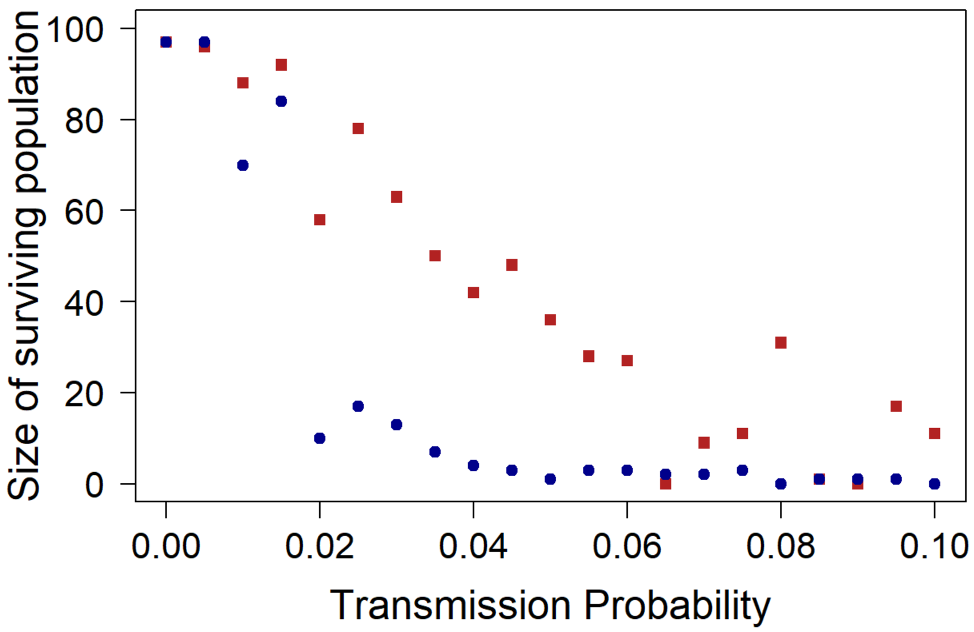


```
par(mfrow=c(1,1),mar=c(0, 0, 0, 0))
plot(graph.adjacency(network1,mode="undirected"),
     vertex.color="firebrick",
     layout=layout.n,vertex.label=NA,vertex.size=8,vertex.shape="square")
text(-0.75,-0.75,"density=0.0914",cex=1.5)
```

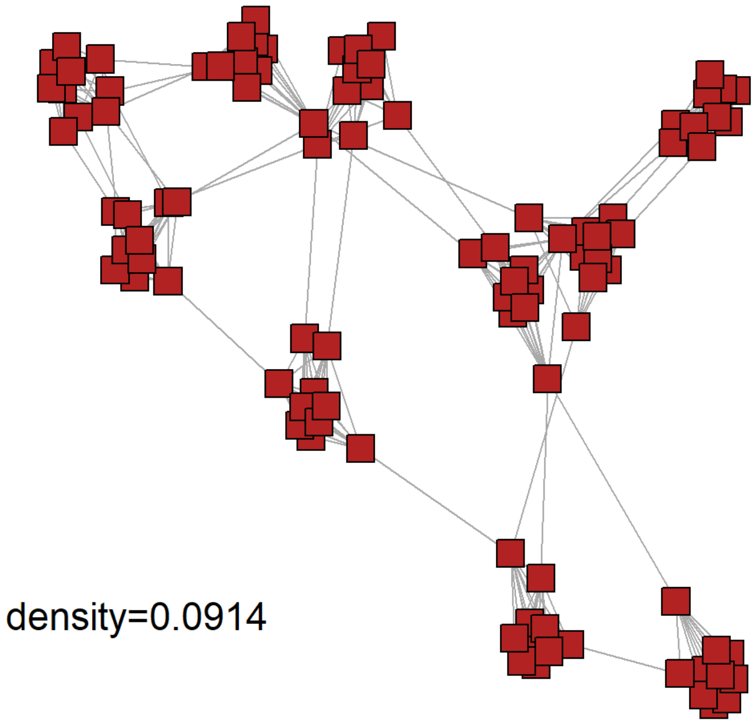


```r
plot(graph.adjacency(network,mode="undirected"),
     vertex.color="dark blue",
     layout=layout.n,vertex.label=NA,vertex.size=8)
text(-0.75,-0.75,"density=0.094",cex=1.5)
```

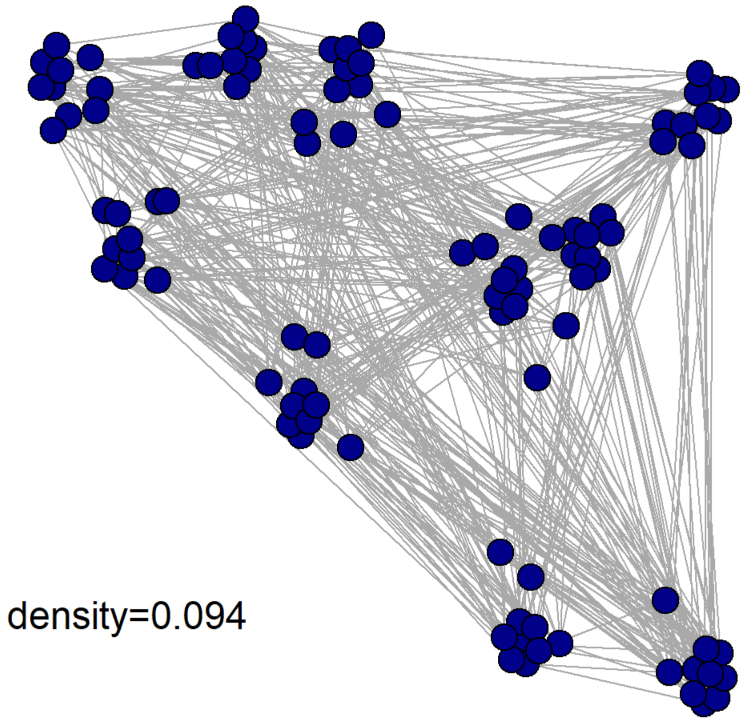

Supplement: Epidemiological Network Models for Conservation Supplementary Material 2 [file rstb20180211supp2.pdf]
