# Supplementary material for: Integrating social behaviour, demography and disease dynamics in network models: applications to disease management in declining wildlife populations
Source: Philos Trans R Soc Lond B Biol Sci. 2019 Jul 29;374(1781):20180211. doi: 10.1098/rstb.2018.0211 (PMC6710568; doi:10.1098/rstb.2018.0211)
Supplement: Epidemiological Network Models for Conservation Supplementary Material 3 [file rstb20180211supp3.pdf]

Epidemiological Network models for Conservation: Supplementary Material 3

Matthew Silk

26 February 2019

In this document we provide the code for a more complex network model of infection with a demographic component. We use this to set up an endemic infection within a small population and then demonstrate the potential effects of increasing of host social structure or pathogen virulence on the dynamics of the previously stable host-pathogen system

---

First load the required packages and we set.seed() for consistency

```
library(igraph)
library(boot)

set.seed(8)
```

We now define a function (pop.gen) that generates a population consisting of ***n.groups*** subpopulations of ***s.groups*** size. ***n.I*** individuals are initially infected as per our simple model

```r
#Function to set up a population

pop.gen<-function(n.groups=10,s.groups=10,n.I=1){

 # defines location of groups
 poss.x<-rep(seq(1,n.groups,1),each=n.groups)
 poss.y<-rep(seq(1,n.groups,1),n.groups)
 poss.locs<-cbind(poss.x,poss.y)
 locs<-sample(seq(1,n.groups^2,1),n.groups,replace=F)
 group.locs<-data.frame(seq(1,n.groups,1),poss.locs[locs,])

 #calculates population size
 pop<-n.groups*s.groups

 #creates individuals
 indiv.ID<-seq(1,pop,1)

 #assigns individuals to groups
 indiv.GR<-rep(1:n.groups,each=s.groups)

 #assigns individuals their correct group locations
 indiv.X<-rep(NA,pop)
 indiv.Y<-rep(NA,pop)
 for(i in 1:pop){
  indiv.X[i]<-group.locs[group.locs[,1]==indiv.GR[i],2]
  indiv.Y[i]<-group.locs[group.locs[,1]==indiv.GR[i],3]
 }

 #creates dataframe containing population info
 indiv.info<-data.frame(indiv.ID,indiv.GR,indiv.X,indiv.Y)
 names(indiv.info)<-c("ID","Group","X","Y")

 #create initially infected individuals and generate SIR + D info
 I.I<-sample(1:pop,n.I)
 I<-matrix(0,nr=pop,nc=1)
 I[I.I]<-1
 S<-1-I
 R<-matrix(0,nr=pop,nc=1)

 #combine into dataframe and return dataframe and group locations as a list
 indiv.info<-data.frame(indiv.info,S,I,R)

 p<-list(indiv.info,group.locs)

 return(p)

}
```

We then define a function that uses the population information (*pop* and *indiv.info*) and a set of edge probabilities (*p.ig*,*p.og*,*dist.eff*) to generate a contact network for the population. Setting plot=T will provide an image of the network generated

- **p.ig** is the probability of within subpopulation edges
- **p.og** is the probability of between subpopulation edges
- **dist.eff** is the effect of distance between subpopulations on the probability of between subpopulation edges

```r
net.gen<-function(pop,indiv.info,group.locs,p.ig,p.og,dist.eff,plot=T){

 network<-matrix(0,nr=pop,nc=pop)

 rownames(network)<-colnames(network)<-indiv.info$ID

 for(i in 1:(nrow(network)-1)){
  for(j in (i+1):nrow(network)){
   if(indiv.info$Group[indiv.info$ID==rownames(network)[i]]==indiv.info$Group[indiv.info$ID==colnames(networ
k)[j]]){
    tmp<-p.ig
    network[i,j]<-rbern(1,tmp)
   }
   if(indiv.info$Group[indiv.info$ID==rownames(network)[i]]!=indiv.info$Group[indiv.info$ID==colnames(networ
k)[j]]){
    tmp.d<-dist(group.locs[c(indiv.info$Group[indiv.info$ID==rownames(network)[i]],
                            indiv.info$Group[indiv.info$ID==colnames(network)[j]]),2:3])
    tmp<-p.og*exp(dist.eff*tmp.d)
    network[i,j]<-rbern(1,tmp)
   }

   network[j,i]<-network[i,j]

  }
 }

 diag(network)<-0

 if(plot==T){
 #dev.new()
  plot(graph.adjacency(network,mode="undirected"),vertex.color=pop.info[[1]]$I,vertex.label=NA,vertex.size=4
)
 }

 return(network)

}
```

We now write a function that governs the disease transmission process occurring at each time step. This function requires information on the population, the contact network, and the groups and their locations.

We now consider R to be removed (i.e. dead) so that both suscpetible and infected individuals can transition to the R state

It also uses two dsease parameters

- ***S_I*** governs the probability per time-step of a susceptible individual becoming infected if connected to an infected node in the contact network
- ***S_R*** governs probability per time-step of a susceptible individual transitioning to the removed state
- ***I_R*** governs the additional probability per time-step of an infected individual transitioning to the removed state (i.e. P(I->R)=S_R+I_R)

```
ts<-function(network,indiv.info,S_I,S_R,I_R,plot=T){

 S<-indiv.info$S
 I<-indiv.info$I
 R<-indiv.info$R

 t.mat<-array(0,dim=dim(network))
 for(i in 1:nrow(network)){
  for(j in 1:nrow(network)){
   t.mat[i,j]<-rbern(1,S_I)*network[i,j]
  }
 }

 diag(t.mat)<-0

 danger<-t.mat[which(I>0),]
 ifelse(is.vector(danger)==TRUE,infected<-which(danger>0),infected<-which(colSums(danger)>0))

 if(length(infected)>0){
  for(i in 1:length(infected)){
   if(R[infected[i]]==0){
    I[infected[i]]<-1
    S[infected[i]]<-0
   }
  }
 }

 for(i in 1:nrow(indiv.info)){
  if(I[i]==1){
   R[i]<-rbern(1,S_R+I_R)
   if(R[i]==1){
    I[i]<-0
   }
  }
  if(S[i]==1){
   R[i]<-rbern(1,S_R)
   if(R[i]==1){
    S[i]<-0
   }
  }
 }

 indiv.info2<-indiv.info
 indiv.info2$S<-S
 indiv.info2$I<-I
 indiv.info2$R<-R

 res<-list(indiv.info2)
 return(res)

} #end function
```

We now write a function that controls recruitment into the population.

- Recruitment is controlled by a birth rate (BR) which we will set below. We recommend setting BR as a function of S_R (and possibly I_R) to have closer control over population growth rates. It also helps to set recruitment to be density dependent. This has to be done by calculating population size at each time step and using that to calculate birth rate.
- New recruits are added to groups with probabilities equal to the inverse of current group size. This means that recruitment is to some extent density-dependent at a social group level
- New individuals are added to the social network with the same spatial rules used to construct the network in the first place

```
recruit<-function(network,indiv.info,BR,group.locs,p.ig,p.og,dist.eff,plot=T){
  indiv.info2<-indiv.info
  indiv.info2a<-indiv.info2[indiv.info2$R==0,]
  indiv.info2b<-indiv.info2[indiv.info2$R==1,]
  BR2<-inv.logit(BR)
  new.indivs<-sum(rbern(nrow(indiv.info2a),BR2))

  if(new.indivs>0){

    #update population info
    id.new<-seq(max(indiv.info2$ID)+1,max(indiv.info2$ID)+new.indivs,1)
    tmp.gs<-table(indiv.info2a$Group)
    group.new<-sample(unique(indiv.info2a$Group),new.indivs,replace=TRUE,prob=1/tmp.gs)
    X.new<-group.locs[group.new,2]
    Y.new<-group.locs[group.new,3]
    S.new<-rep(1,new.indivs)
    I.new<-rep(0,new.indivs)
    R.new<-rep(0,new.indivs)
    indiv.info2c<-data.frame(id.new,group.new,X.new,Y.new,S.new,I.new,R.new)
    names(indiv.info2c)<-names(indiv.info)
    indiv.info3<-rbind(indiv.info2a,indiv.info2c)

    if(nrow(indiv.info2b)>0){
     #update network
     remove<-which(indiv.info2$ID%in%indiv.info2b$ID==TRUE)
     network2<-network[-remove,-remove]
    }
    if(nrow(indiv.info2b)==0){
      network2<-network
    }

    network3<-matrix(0,nr=nrow(indiv.info3),nc=nrow(indiv.info3))
    network3[1:nrow(indiv.info2a),1:nrow(indiv.info2a)]<-network2
    rownames(network3)<-colnames(network3)<-indiv.info3$ID

    for(i in (nrow(indiv.info2a)+1):nrow(indiv.info3)){
      for(j in 1:nrow(indiv.info3)){
       if(indiv.info3$Group[indiv.info3$ID==rownames(network3)[i]]==indiv.info3$Group[indiv.info3$ID==colnam
es(network3)[j]]){
        tmp<-p.ig
        network3[i,j]<-rbern(1,tmp)
       }
       if(indiv.info3$Group[indiv.info3$ID==rownames(network3)[i]]!=indiv.info3$Group[indiv.info3$ID==colnam
es(network3)[j]]){
        tmp.d<-dist(group.locs[c(indiv.info3$Group[indiv.info3$ID==rownames(network3)[i]],
                             indiv.info3$Group[indiv.info3$ID==colnames(network3)[j]]),2:3])
        tmp<-p.og*exp(dist.eff*tmp.d)
        network3[i,j]<-rbern(1,tmp)
       }
       network3[j,i]<-network3[i,j]
      }
    }
    diag(network3)<-0

    if(plot==T){
     plot(graph.adjacency(network3,mode="undirected"),vertex.color=pop.info[[1]]$I,vertex.label=NA,vertex.si
ze=4)
    }

    networkF<-network3
    indiv.infoF<-indiv.info3
  }
  if(new.indivs==0){
    networkF<-network
    indiv.infoF<-indiv.info2
  }

  recr<-list(indiv.infoF,networkF)
  return(recr)
}
```

Define a Bernoulli draw function for convenience

```
rbern<-function(n,prob){
  return(rbinom(n,1,prob))
}
```

In this section of the code we define the key parameters - these are the ones to change to adjust network and disease characteristics

```
#PROBABILITY OF WITHIN SUBPOPULATION CONTACTS
p.ig<-0.65

#PROBABILITY OF BETWEEN SUBPOPULATION CONTACTS
p.og<-0.01

#DISTANCE EFFECT ON BETWEEN SUBPOPULATION CONTACTS
dist.eff<- -0.3 #plugged into exponential decay

#PROBABILITY OF SUCEPTIBLE INDIVIDUAL BEING INFECTED OVER AN EDGE PER TIME-STEP
S_I<-0.008

#MORTALITY OF SUSCEPTIBLE INDIVIDUAL
S_R<-0.01

#ADDITIONAL DISEASE MORTALITY PER TIME-STEP
I_R<-0.005

#BIRTH RATE - provide on logit scale as value desired at stable population size
BR<-logit(S_R*1.01)
```

We now set up the population and its contact network. The population is 100 individuals, consisting of 10 subpopulations of 10 individuals.3 individuals are initially infected

```
#create empty list to store outputs every time step
pop.info<-list()

n.groups<-10
s.groups<-10

#create initial population
tmp.p<-pop.gen(n.groups,s.groups,n.I=3)

#and store the output
pop.info[[1]]<-tmp.p[[1]]
group.locs<-tmp.p[[2]]

#create intitial network
network<-net.gen(pop=nrow(pop.info[[1]]),indiv.info=pop.info[[1]],group.locs=group.locs,p.ig,p.og,dist.eff,p
lot=T)
```

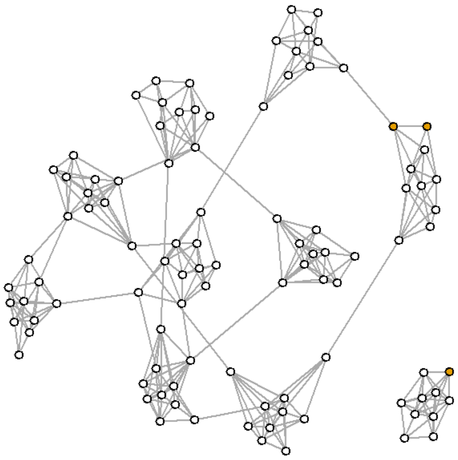

We now run transmission/transition and recruitment algorithms for 800 time-steps (running the first set of algorithms separately to check that they are working) until the disease is endemic in the population and prevalence is stable (to some extent)

```
tmp.up<-ts(network=network,indiv.info=pop.info[[1]],S_I=S_I,S_R=S_R,I_R=I_R,plot=F)
tmp.up2<-recruit(network=network,indiv.info=tmp.up[[1]],BR=BR+(100-sum(tmp.up[[1]]$R==0))/1000,group.locs=gr
oup.locs,p.ig=p.ig,p.og=p.og,dist.eff=dist.eff,plot=F)

pop.info[[2]]<-tmp.up2[[1]]
network<-tmp.up2[[2]]

for(t in 3:800){
 #if(sum(tmp.up2[[1]]$I)==0){break}
 tmp.up<-ts(network=network,indiv.info=tmp.up2[[1]],S_I=S_I,S_R=S_R,I_R=I_R,plot=F)
 tmp.up2<-recruit(network=network,indiv.info=tmp.up[[1]],BR=BR+(100-sum(tmp.up[[1]]$R==0))/1000,group.locs=g
roup.locs,p.ig=p.ig,p.og=p.og,
                  dist.eff=dist.eff,plot=F)

 pop.info[[t]]<-tmp.up2[[1]]
 network<-tmp.up2[[2]]
 if(t%%100==0){print(t)}
}

network2<-network
```

Look at plots of population and disease dynamics over time to examine the epidemiology of the infection

- Host population size over time
- Disease prevalence over time (of the population)
- Disease prevalence in each group over time

```
POPS<-matrix(unlist(lapply(pop.info,dim)),nr=length(pop.info),nc=2,byrow=T)
POPS<-POPS[,1]

plot(POPS,type="l",ylab="Population Size",xlab="Time")
```

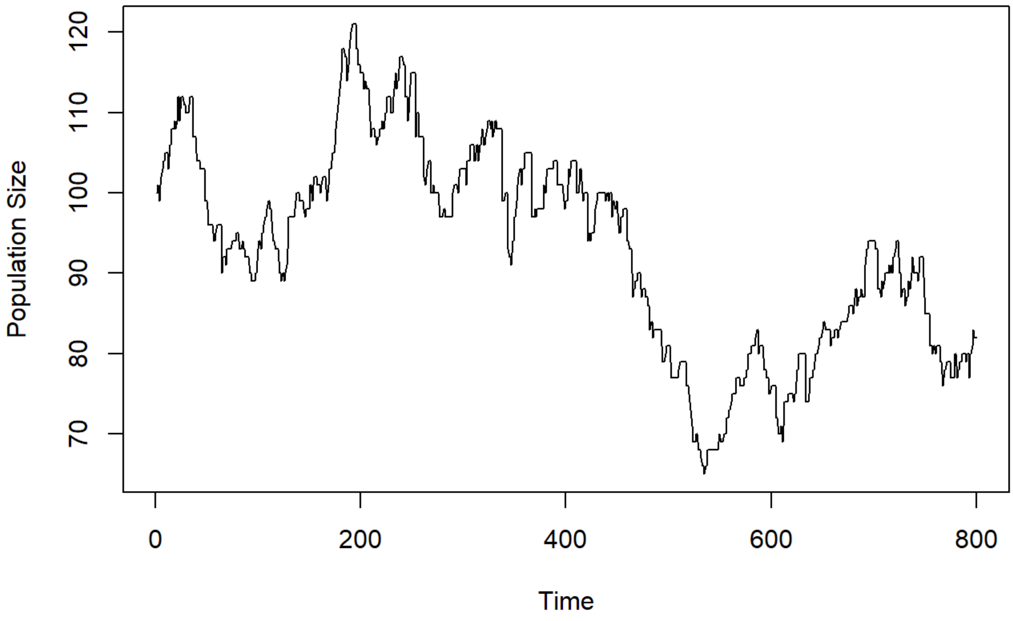


```
OUT<-matrix(unlist(lapply(pop.info,colSums)),nr=length(pop.info),nc=7,byrow = T)
plot(OUT[,6],type="l")
```

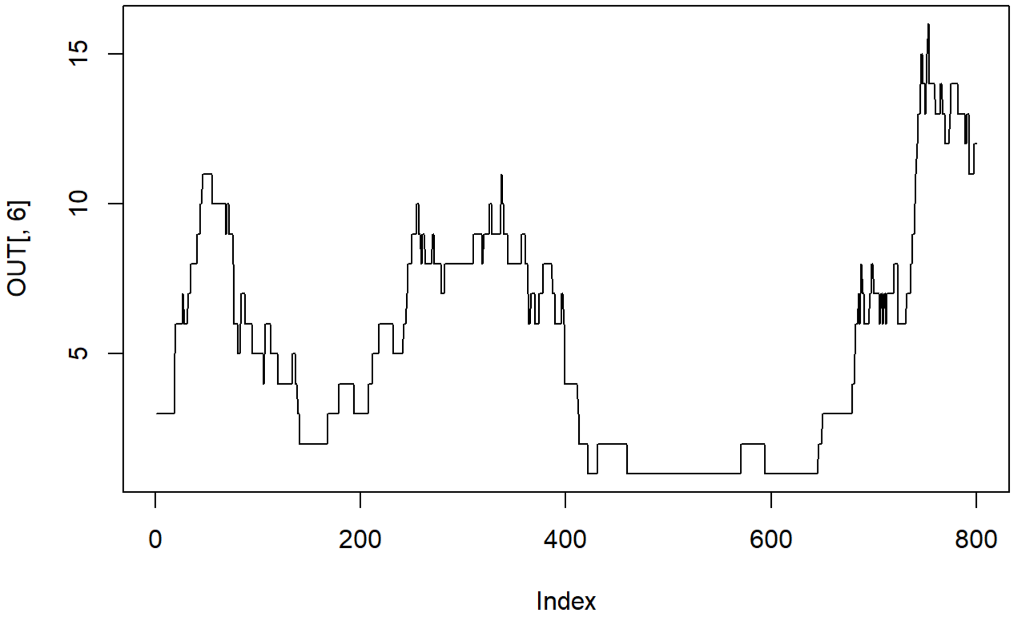


```
prev<-OUT[,6]/POPS
plot(prev,type="l",ylim=c(0,1),ylab="Disease Prevalence",xlab="Time")
```

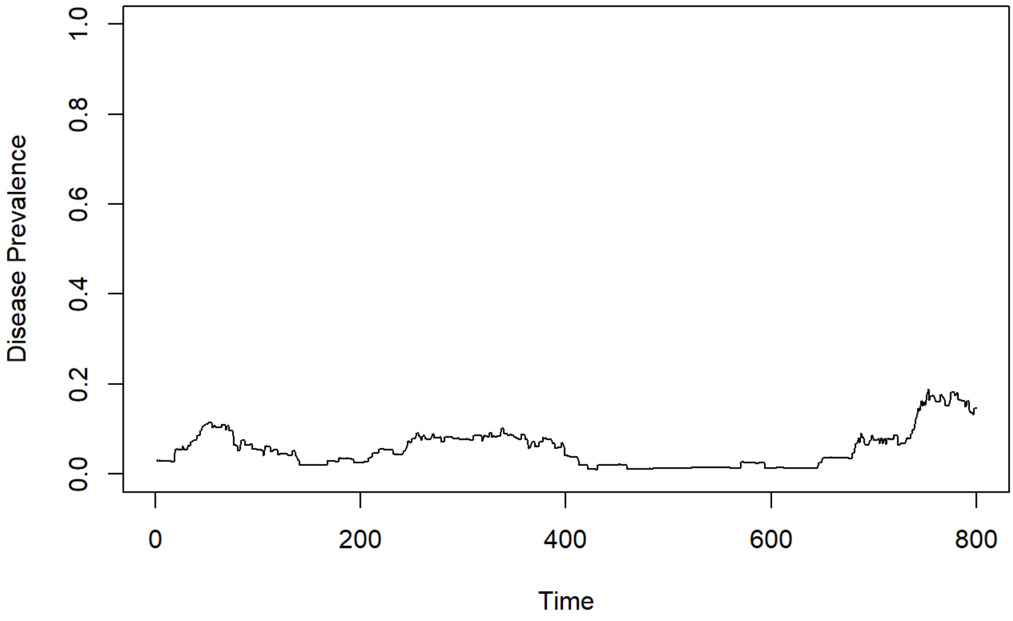


```
gprev<-function(dat){
  calc<-aggregate(dat$I,by=list(dat$Group),mean)
  op<-matrix(c(seq(1:10),rep(0,10)),nr=10,nc=2)
  for(ii in 1:dim(calc)[1]){
    op[calc[ii,1],2]<-calc[ii,2]
  }
  return(op)
}

GR.OUT<-matrix(unlist(lapply(pop.info,gprev)),nr=length(pop.info)*2,nc=10,byrow=TRUE)

GR.OUT<-GR.OUT[seq(2,length(pop.info)*2,2),]

par(mfrow=c(2,5),mar=c(3,3,1,1))
for(i in 1:10){
 plot(GR.OUT[,i],xlim=c(0,1200),ylim=c(0,1),xlab="Time",ylab="Prevalence",col="dark blue",main=paste("Group"
,i),type="l")
}
```

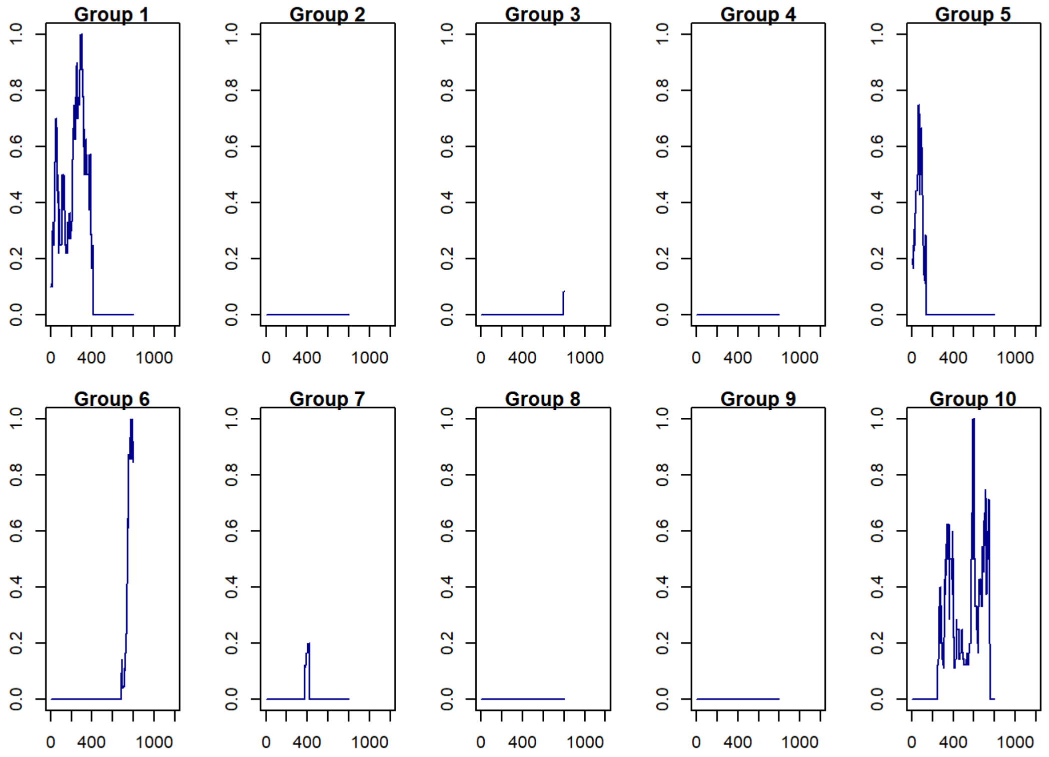


```
par(mfrow=c(1,1))
```

Post-environmental change code

Here we allow the model to forecast disease and host population dynamics after changes to pathogen virulence and/or host social connectivity

First we set up the four prospective sets of future conditions:

1. No change (control)
2. Increased pathogen virulence or reduced host tolerance. We increase the value of I_R.
3. Increased (between-group) social connectivity of the host population. We increase the value of p.og
4. Increased virulence and social connectivity. We increase both I_R and p.og.

```
#PROBABILITY OF BETWEEN SUBPOPULATION CONTACTS
p.og2<-c(0.01,0.01,0.03,0.03)

#S_I2<-c(0.008,0.016,0.008,0.016)

#ADDITIONAL DISEASE MORTALITY PER TIME-STEP
I_R2<-c(0.005,0.015,0.005,0.015)
```

Here we set up 50 replicates of our four sets of future conditions and and continue the simulation for 400 timesteps in each case with the new parameter values.

```
ENV.RES<-list()
pos<-1

for(par in 1:4){
 for(rep in 1:50){

  pop.info<-pop.info[1:800]

  network<-net.gen(pop=nrow(pop.info[[800]]),indiv.info=pop.info[[800]],group.locs=group.locs,p.ig,p.og2[par
],dist.eff,plot=F)

  tmp.up<-ts(network=network,indiv.info=pop.info[[800]],S_I=S_I,S_R=S_R,I_R=I_R2[par],plot=F)
  tmp.up2<-recruit(network=network,indiv.info=tmp.up[[1]],BR=BR+(100-sum(tmp.up[[1]]$R==0))/1000,group.locs=
group.locs,
                 p.ig=p.ig,p.og=p.og2[par],dist.eff=dist.eff,plot=F)

  pop.info[[801]]<-tmp.up2[[1]]
  network<-tmp.up2[[2]]

  for(t in 2:400){
   tmp.up<-ts(network=network,indiv.info=tmp.up2[[1]],S_I=S_I,S_R=S_R,I_R=I_R2[par],plot=F)
   tmp.up2<-recruit(network=network,indiv.info=tmp.up[[1]],BR=BR+(100-sum(tmp.up[[1]]$R==0))/1000,group.locs
=group.locs,
                  p.ig=p.ig,p.og=p.og2[par],dist.eff=dist.eff,plot=F)

   pop.info[[800+t]]<-tmp.up2[[1]]
   network<-tmp.up2[[2]]

  }

  ENV.RES[[pos]]<-pop.info[801:(t+800)]

  pos<-pos+1

 }
}
```

Here we collate the results from our different model forecasts

```
pars<-rep(rep(seq(1,4,1),each=50),400)
reps<-rep(seq(1,50,1),4*400)
tss<-rep(seq(1,400,1),each=4*50)
prevs<-rep(NA,length(pars))
pops<-rep(NA,length(pars))

env.out<-data.frame(pars,reps,tss,prevs,pops)

pa<-rep(seq(1,4,1),each=50)
rp<-rep(1:50,4)

for(rs in 1:200){

POPS<-matrix(unlist(lapply(ENV.RES[[rs]],dim)),nr=length(ENV.RES[[rs]]),nc=2,byrow=T)
POPS<-POPS[,1]

OUT<-matrix(unlist(lapply(ENV.RES[[rs]],colSums)),nr=length(ENV.RES[[rs]]),nc=7,byrow = T)

prev<-OUT[,6]/POPS

env.out$prevs[which(env.out$pars==pa[rs]&env.out$reps==rp[rs])]<-prev
env.out$pops[env.out$pars==pa[rs]&env.out$reps==rp[rs]]<-POPS

}
```

This section of code plots the equivalent of Fig. 3 in the main text (code may need altering if parameter values are changed by the user)

```
sum.res<-aggregate(env.out$prevs,by=list(env.out$tss,env.out$pars),mean)
dim(sum.res)

col3=c("#4477AA", "#DDCC77", "#CC6677")
colA<-col2rgb("grey")/255
colB<-col2rgb(col3[1])/255
colC<-col2rgb(col3[2])/255
colD<-col2rgb(col3[3])/255

par(mfrow=c(1,2),mar=c(5,5,1,1))

plot(sum.res[sum.res[,2]==1,3],xlim=c(0,400),ylim=c(0,0.7),type="l",col="grey",lwd=3,las=1,cex.axis=1.4,cex.
lab=1.7,ylab="Disease prevalence",xlab="Time Step")
points(env.out$tss[env.out$tss%in%c(100,200,300,400)&env.out$pars==1&env.out$reps<26]-3.5,
       env.out$prevs[env.out$tss%in%c(100,200,300,400)&env.out$pars==1&env.out$reps<26],col=rgb(colA[1],colA
[2],colA[3],0.5),pch=4,cex=0.7,lwd=2)
points(env.out$tss[env.out$tss%in%c(100,200,300,400)&env.out$pars==2&env.out$reps<26]-1.5,
       env.out$prevs[env.out$tss%in%c(100,200,300,400)&env.out$pars==2&env.out$reps<26],col=rgb(colB[1],colB
[2],colB[3],0.5),pch=4,cex=0.7,lwd=2)
points(env.out$tss[env.out$tss%in%c(100,200,300,400)&env.out$pars==3&env.out$reps<26]+1.5,
       env.out$prevs[env.out$tss%in%c(100,200,300,400)&env.out$pars==3&env.out$reps<26],col=rgb(colC[1],colC
[2],colC[3],0.5),pch=4,cex=0.7,lwd=2)
points(env.out$tss[env.out$tss%in%c(100,200,300,400)&env.out$pars==4&env.out$reps<26]+3.5,
       env.out$prevs[env.out$tss%in%c(100,200,300,400)&env.out$pars==4&env.out$reps<26],col=rgb(colD[1],colD
[2],colD[3],0.5),pch=4,cex=0.7,lwd=2)
lines(sum.res[sum.res[,2]==1,3],col="grey",lwd=3)
lines(sum.res[sum.res[,2]==2,3],col=col3[1],lwd=3)
lines(sum.res[sum.res[,2]==3,3],col=col3[2],lwd=3)
lines(sum.res[sum.res[,2]==4,3],col=col3[3],lwd=3)


sum.res2<-aggregate(env.out$pops,by=list(env.out$tss,env.out$pars),mean)

plot(sum.res2[sum.res2[,2]==1,3],xlim=c(0,400),ylim=c(0,120),type="l",col="grey",lwd=3,las=1,cex.axis=1.4,ce
x.lab=1.7,ylab="Host population size",xlab="Time Step")

points(env.out$tss[env.out$tss%in%c(100,200,300,400)&env.out$pars==1&env.out$reps<26]-3.5,
       env.out$pops[env.out$tss%in%c(100,200,300,400)&env.out$pars==1&env.out$reps<26],col=rgb(colA[1],colA[
2],colA[3],0.5),pch=4,cex=0.7,lwd=2)
points(env.out$tss[env.out$tss%in%c(100,200,300,400)&env.out$pars==2&env.out$reps<26]-1.5,
       env.out$pops[env.out$tss%in%c(100,200,300,400)&env.out$pars==2&env.out$reps<26],col=rgb(colB[1],colB[
2],colB[3],0.5),pch=4,cex=0.7,lwd=2)
points(env.out$tss[env.out$tss%in%c(100,200,300,400)&env.out$pars==3&env.out$reps<26]+1.5,
       env.out$pops[env.out$tss%in%c(100,200,300,400)&env.out$pars==3&env.out$reps<26],col=rgb(colC[1],colC[
2],colC[3],0.5),pch=4,cex=0.7,lwd=2)
points(env.out$tss[env.out$tss%in%c(100,200,300,400)&env.out$pars==4&env.out$reps<26]+3.5,
       env.out$pops[env.out$tss%in%c(100,200,300,400)&env.out$pars==4&env.out$reps<26],col=rgb(colD[1],colD[
2],colD[3],0.5),pch=4,cex=0.7,lwd=2)
lines(sum.res2[sum.res2[,2]==1,3],col="grey",lwd=3)
lines(sum.res2[sum.res2[,2]==2,3],col=col3[1],lwd=3)
lines(sum.res2[sum.res2[,2]==3,3],col=col3[2],lwd=3)
lines(sum.res2[sum.res2[,2]==4,3],col=col3[3],lwd=3)
```

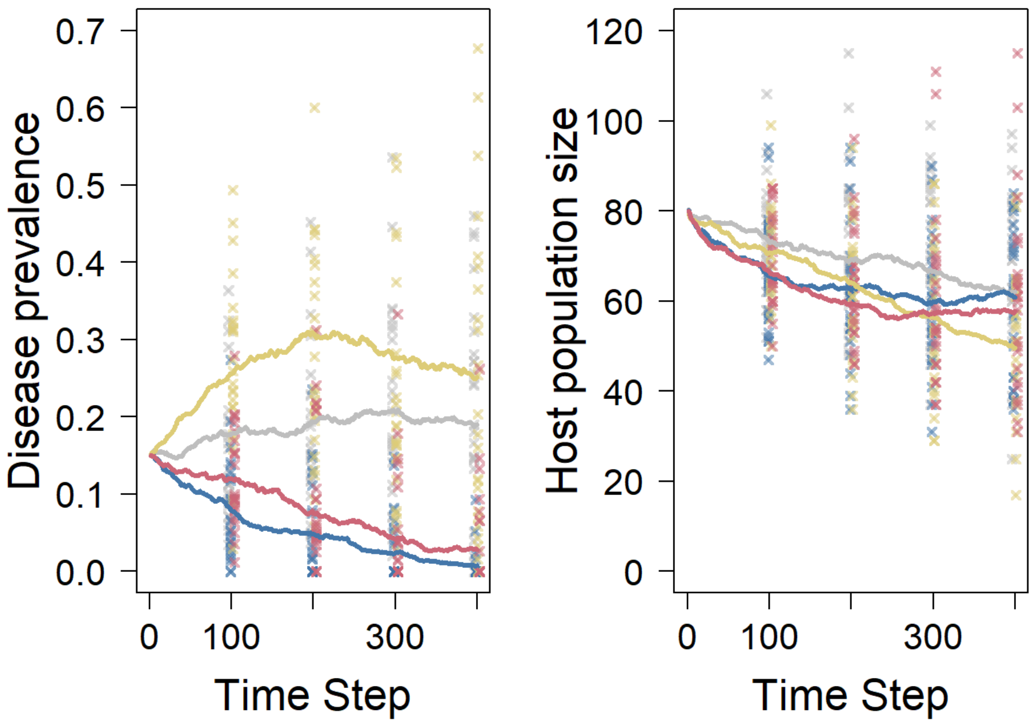

Disease prevalence
0.7
0.6
0.5
0.4
0.3
0.2
0.1
0.0
0
100
300
Time Step
Host population size
120
100
80
60
40
20
0
0
100
300
Time Step
