# Supplementary material for: Integrating social behaviour, demography and disease dynamics in network models: applications to disease management in declining wildlife populations
Source: Philos Trans R Soc Lond B Biol Sci. 2019 Jul 29;374(1781):20180211. doi: 10.1098/rstb.2018.0211 (PMC6710568; doi:10.1098/rstb.2018.0211)
Supplement: Epidemiological Network Models for Conservation Supplementary Material 3 [file rstb20180211supp4.pdf]

Epidemiological Network models for Conservation: Supplementary Material 4

Matthew Silk

26 February 2019

In this document we adapt the same more complex network model of infection with a demographic component to model how network-targeted vaccination can aid in the management of an epidemic of a new emerging pathogen.

First load the required packages and we set.seed() for consistency

```
library(igraph)
library(boot)

set.seed(3)
```

Functions for SIR network-demographic model

We now define a function (pop.gen) that generates a population consisting of ***n.groups*** subpopulations of ***s.groups*** size. ***n.I*** individuals are initially infected as per our simple model

```r
#Function to set up a population

pop.gen<-function(n.groups=10,s.groups=10,n.I=1){

 # defines location of groups
 poss.x<-rep(seq(1,n.groups,1),each=n.groups)
 poss.y<-rep(seq(1,n.groups,1),n.groups)
 poss.locs<-cbind(poss.x,poss.y)
 locs<-sample(seq(1,n.groups^2,1),n.groups,replace=F)
 group.locs<-data.frame(seq(1,n.groups,1),poss.locs[locs,])

 #calculates population size
 pop<-n.groups*s.groups

 #creates individuals
 indiv.ID<-seq(1,pop,1)

 #assigns individuals to groups
 indiv.GR<-rep(1:n.groups,each=s.groups)

 #assigns individuals their correct group locations
 indiv.X<-rep(NA,pop)
 indiv.Y<-rep(NA,pop)
 for(i in 1:pop){
  indiv.X[i]<-group.locs[group.locs[,1]==indiv.GR[i],2]
  indiv.Y[i]<-group.locs[group.locs[,1]==indiv.GR[i],3]
 }

 #creates dataframe containing population info
 indiv.info<-data.frame(indiv.ID,indiv.GR,indiv.X,indiv.Y)
 names(indiv.info)<-c("ID","Group","X","Y")

 #create initially infected individuals and generate SIR + D info
 I.I<-sample(1:pop,n.I)
 I<-matrix(0,nr=pop,nc=1)
 I[I.I]<-1
 S<-1-I
 R<-matrix(0,nr=pop,nc=1)

 #combine into dataframe and return dataframe and group locations as a list
 indiv.info<-data.frame(indiv.info,S,I,R)

 p<-list(indiv.info,group.locs)

 return(p)

}
```

We then define a function that uses the population information (*pop* and *indiv.info*) and a set of edge probabilities (*p.ig*,*p.og*,*dist.eff*) to generate a contact network for the population. Setting plot=T will provide an image of the network generated

- **p.ig** is the probability of within subpopulation edges
- **p.og** is the probability of between subpopulation edges
- **dist.eff** is the effect of distance between subpopulations on the probability of between subpopulation edges

```r
net.gen<-function(pop,indiv.info,group.locs,p.ig,p.og,dist.eff,plot=T){

 network<-matrix(0,nr=pop,nc=pop)

 rownames(network)<-colnames(network)<-indiv.info$ID

 for(i in 1:(nrow(network)-1)){
  for(j in (i+1):nrow(network)){
   if(indiv.info$Group[indiv.info$ID==rownames(network)[i]]==indiv.info$Group[indiv.info$ID==colnames(networ
k)[j]]){
    tmp<-p.ig
    network[i,j]<-rbern(1,tmp)
   }
   if(indiv.info$Group[indiv.info$ID==rownames(network)[i]]!=indiv.info$Group[indiv.info$ID==colnames(networ
k)[j]]){
    tmp.d<-dist(group.locs[c(indiv.info$Group[indiv.info$ID==rownames(network)[i]],
                             indiv.info$Group[indiv.info$ID==colnames(network)[j]]),2:3])
    tmp<-p.og*exp(dist.eff*tmp.d)
    network[i,j]<-rbern(1,tmp)
   }

   network[j,i]<-network[i,j]

  }
 }

 diag(network)<-0

 if(plot==T){
 #dev.new()
  plot(graph.adjacency(network,mode="undirected"),vertex.color=pop.info[[1]]$I,vertex.label=NA,vertex.size=4
)
 }

 return(network)

}
```

We now write a function that governs the disease transmission process occurring at each time step. This function requires information on the population, the contact network, and the groups and their locations.

We now consider R to be removed (i.e. dead) so that both suscpetible and infected individuals can transition to the R state

It also uses two dsease parameters

- ***S_I*** governs the probability per time-step of a susceptible individual becoming infected if connected to an infected node in the contact network
- ***S_R*** governs probability per time-step of a susceptible individual transitioning to the removed state
- ***I_R*** governs the additional probability per time-step of an infected individual transitioning to the removed state (i.e. P(I->R)=S_R+I_R)

```
ts<-function(network,indiv.info,S_I,S_R,I_R,plot=T){

 S<-indiv.info$S
 I<-indiv.info$I
 R<-indiv.info$R

 t.mat<-array(0,dim=dim(network))
 for(i in 1:nrow(network)){
  for(j in 1:nrow(network)){
   t.mat[i,j]<-rbern(1,S_I)*network[i,j]
  }
 }

 diag(t.mat)<-0

 danger<-t.mat[which(I>0),]
 ifelse(is.vector(danger)==TRUE,infected<-which(danger>0),infected<-which(colSums(danger)>0))

 if(length(infected)>0){
  for(i in 1:length(infected)){
   if(R[infected[i]]==0){
    I[infected[i]]<-1
    S[infected[i]]<-0
   }
  }
 }

 for(i in 1:nrow(indiv.info)){
  if(I[i]==1){
   R[i]<-rbern(1,S_R+I_R)
   if(R[i]==1){
    I[i]<-0
   }
  }
  if(S[i]==1){
   R[i]<-rbern(1,S_R)
   if(R[i]==1){
    S[i]<-0
   }
  }
 }

 indiv.info2<-indiv.info
 indiv.info2$S<-S
 indiv.info2$I<-I
 indiv.info2$R<-R

 res<-list(indiv.info2)
 return(res)

} #end function
```

We now write a function that controls recruitment into the population.

- Recruitment is controlled by a birth rate (BR) which we will set below. We recommend setting BR as a function of S_R (and possibly I_R) to have closer control over population growth rates. It also helps to set recruitment to be density dependent. This has to be done by calculating population size at each time step and using that to calculate birth rate.
- New recruits are added to groups with probabilities equal to the inverse of current group size. This means that recruitment is to some extent density-dependent at a social group level
- New individuals are added to the social network with the same spatial rules used to construct the network in the first place

```
recruit<-function(network,indiv.info,BR,group.locs,p.ig,p.og,dist.eff,plot=T){
  indiv.info2<-indiv.info
  indiv.info2a<-indiv.info2[indiv.info2$R==0,]
  indiv.info2b<-indiv.info2[indiv.info2$R==1,]
  BR2<-inv.logit(BR)
  new.indivs<-sum(rbern(nrow(indiv.info2a),BR2))

  if(new.indivs>0){

    #update population info
```

```
    #update population info
    id.new<-seq(max(indiv.info2$ID)+1,max(indiv.info2$ID)+new.indivs,1)
    tmp.gs<-table(indiv.info2a$Group)
    group.new<-sample(unique(indiv.info2a$Group),new.indivs,replace=TRUE,prob=1/tmp.gs)
    X.new<-group.locs[group.new,2]
    Y.new<-group.locs[group.new,3]
    S.new<-rep(1,new.indivs)
    I.new<-rep(0,new.indivs)
    R.new<-rep(0,new.indivs)
    indiv.info2c<-data.frame(id.new,group.new,X.new,Y.new,S.new,I.new,R.new)
    names(indiv.info2c)<-names(indiv.info)
    indiv.info3<-rbind(indiv.info2a,indiv.info2c)

    if(nrow(indiv.info2b)>0){
     #update network
     remove<-which(indiv.info2$ID%in%indiv.info2b$ID==TRUE)
     network2<-network[-remove,-remove]
    }
    if(nrow(indiv.info2b)==0){
      network2<-network
    }

    ##need to change transmission function to account for the fact that the network will shortly be getting muddled - need to use row names/col names##
    ##or actually I think it is still sorted if we add them on the end each time :-)

    network3<-matrix(0,nr=nrow(indiv.info3),nc=nrow(indiv.info3))
    network3[1:nrow(indiv.info2a),1:nrow(indiv.info2a)]<-network2
    rownames(network3)<-colnames(network3)<-indiv.info3$ID

    for(i in (nrow(indiv.info2a)+1):nrow(indiv.info3)){
      for(j in 1:nrow(indiv.info3)){
       if(indiv.info3$Group[indiv.info3$ID==rownames(network3)[i]]==indiv.info3$Group[indiv.info3$ID==colnames(network3)[j]]){
        tmp<-p.ig
        network3[i,j]<-rbern(1,tmp)
       }
       if(indiv.info3$Group[indiv.info3$ID==rownames(network3)[i]]!=indiv.info3$Group[indiv.info3$ID==colnames(network3)[j]]){
        tmp.d<-dist(group.locs[c(indiv.info3$Group[indiv.info3$ID==rownames(network3)[i]],
                             indiv.info3$Group[indiv.info3$ID==colnames(network3)[j]]),2:3])
        tmp<-p.og*exp(dist.eff*tmp.d)
        network3[i,j]<-rbern(1,tmp)
       }
       network3[j,i]<-network3[i,j]
      }
    }
    diag(network3)<-0

    if(plot==T){
     #dev.new()
     plot(graph.adjacency(network3,mode="undirected"),vertex.color=pop.info[[1]]$I,vertex.label=NA,vertex.size=4)
    }

    networkF<-network3
    indiv.infoF<-indiv.info3
  }
  if(new.indivs==0){
    networkF<-network
    indiv.infoF<-indiv.info2
  }

  recr<-list(indiv.infoF,networkF)
  return(recr)
}
```

Define a Bernoulli draw function for convenience

```
rbern<-function(n,prob){
  return(rbinom(n,1,prob))
}
```

Functions for adjusted SIRV network-demographic model

We now write a function that calculates the degree or betweenness of all individuals in the population and then ranks individuals by their values (this is then used as an input to the vaccination function)

- Input is simply the network and a choice of metric to be calculated (degree or betweenness only)

```
met.calc<-function(network,mets=c("D","B")){

  net<-graph.adjacency(network)
  Deg<-degree(net)
  Bet<-betweenness(net)
  Deg2<-data.frame(rownames(network),Deg)
  Bet2<-data.frame(rownames(network),Bet)
  Deg3<-Deg2[rev(order(Deg2$Deg)),]
  Bet3<-Bet2[rev(order(Bet2$Bet)),]

  if(mets=="D"){
    return(Deg3)
  }
  if(mets=="B"){
    return(Bet3)
  }
  else{
    ret.met<list(Deg3,Bet3)
    return(ret.met)
  }
}
```

We now write the function that conducts a vaccination programme

- indiv.info is the current population data frame
- type can take the values “R” for random or “N” for network-targeted
- metric can take the values “D” for degree or “B” for betweenness and is only applied if type==“N”
- v.prop is the proportion of the population to be vaccinated (values in the range 0-1)
- v.eff is the efficacy of the vaccine (values in the range 0-1)

```
vacc<-function(indiv.info,type=c("R","N"),metric=c("D","B"),metric.info,v.prop,v.eff){

  if(v.prop>0){

  vac.size<-floor(nrow(indiv.info)*v.prop)

  if(type=="R"){
    vac.samp<-sort(sample(1:nrow(indiv.info),vac.size,replace=FALSE))
    for(i in 1:length(vac.samp)){
      if(indiv.info$S[vac.samp[i]]==1){
        indiv.info$V[vac.samp[i]]<-rbern(1,v.eff)
      }
      if(indiv.info$V[vac.samp[i]]==1){
        indiv.info$S[vac.samp[i]]<-0
      }
    }
  }

  if(type=="N"){
    if(metric=="D"){
      vac.samp<-which(indiv.info$ID%in%metric.info[1:vac.size,1]==TRUE)
      for(i in 1:length(vac.samp)){
       if(indiv.info$S[vac.samp[i]]==1){
        indiv.info$V[vac.samp[i]]<-rbern(1,v.eff)
       }
       if(indiv.info$V[vac.samp[i]]==1){
        indiv.info$S[vac.samp[i]]<-0
       }
      }
    }

    if(metric=="B"){
      vac.samp<-which(indiv.info$ID%in%metric.info[1:vac.size,1]==TRUE)
      for(i in 1:length(vac.samp)){
       if(indiv.info$S[vac.samp[i]]==1){
        indiv.info$V[vac.samp[i]]<-rbern(1,v.eff)
       }
       if(indiv.info$V[vac.samp[i]]==1){
        indiv.info$S[vac.samp[i]]<-0
       }
      }
    }
  }

  }

  return(indiv.info)

}
```

We now update the original SIR algorithm to iclude a V state (i.e. an SIRV model)

```
ts2<-function(network,indiv.info,S_I,S_R,I_R,plot=T){

 S<-indiv.info$S
 I<-indiv.info$I
 R<-indiv.info$R
 V<-indiv.info$V

 t.mat<-array(0,dim=dim(network))
 for(i in 1:nrow(network)){
  for(j in 1:nrow(network)){
   t.mat[i,j]<-rbern(1,S_I)*network[i,j]
  }
 }

 diag(t.mat)<-0

 danger<-t.mat[which(I>0),]
 ifelse(is.vector(danger)==TRUE,infected<-which(danger>0),infected<-which(colSums(danger)>0))

 if(length(infected)>0){
  for(i in 1:length(infected)){
   if(S[infected[i]]==1){
    I[infected[i]]<-1
   }
  }
 }

 for(i in 1:nrow(indiv.info)){
  if(I[i]==1){
   S[i]<-0
   R[i]<-rbern(1,S_R+I_R)
   if(R[i]==1){
    I[i]<-0
   }
  }
  if(S[i]==1){
   R[i]<-rbern(1,S_R)
   if(R[i]==1){
    S[i]<-0
   }
  }
  if(V[i]==1){
   R[i]<-rbern(1,S_R)
   if(R[i]==1){
    V[i]<-0
   }
  }
 }

 indiv.info2<-indiv.info
 indiv.info2$S<-S
 indiv.info2$I<-I
 indiv.info2$R<-R
 indiv.info2$V<-V

 res<-list(indiv.info2)
 return(res)

} #end function
```

We now update the recruitment function to include a V state (i.e. for the SIRV model)

```
recruit2<-function(network,indiv.info,BR,group.locs,p.ig,p.og,dist.eff,plot=T){
  indiv.info2<-indiv.info
  indiv.info2a<-indiv.info2[indiv.info2$R==0,]
  indiv.info2b<-indiv.info2[indiv.info2$R==1,]
  BR2<-inv.logit(BR)
  new.indivs<-sum(rbern(nrow(indiv.info2a),BR2))

  if(new.indivs>0){

    #update population info
```

```
    id.new<-seq(max(indiv.info2$ID)+1,max(indiv.info2$ID)+new.indivs,1)
    tmp.gs<-table(indiv.info2a$Group)
    group.new<-sample(unique(indiv.info2a$Group),new.indivs,replace=TRUE,prob=1/tmp.gs)
    X.new<-group.locs[group.new,2]
    Y.new<-group.locs[group.new,3]
    S.new<-rep(1,new.indivs)
    I.new<-rep(0,new.indivs)
    R.new<-rep(0,new.indivs)
    V.new<-rep(0,new.indivs)
    indiv.info2c<-data.frame(id.new,group.new,X.new,Y.new,S.new,I.new,R.new,V.new)
    names(indiv.info2c)<-names(indiv.info)
    indiv.info3<-rbind(indiv.info2a,indiv.info2c)

    if(nrow(indiv.info2b)>0){
     #update network
     remove<-which(indiv.info2$ID%in%indiv.info2b$ID==TRUE)
     network2<-network[-remove,-remove]
    }
    if(nrow(indiv.info2b)==0){
      network2<-network
    }

    ##need to change transmission function to account for the fact that the network will shortly be getting
muddled - need to use row names/col names##
    ##or actually I think it is still sorted if we add them on the end each time :-)

    network3<-matrix(0,nr=nrow(indiv.info3),nc=nrow(indiv.info3))
    network3[1:nrow(indiv.info2a),1:nrow(indiv.info2a)]<-network2
    rownames(network3)<-colnames(network3)<-indiv.info3$ID

    for(i in (nrow(indiv.info2a)+1):nrow(indiv.info3)){
      for(j in 1:nrow(indiv.info3)){
       if(indiv.info3$Group[indiv.info3$ID==rownames(network3)[i]]==indiv.info3$Group[indiv.info3$ID==colnam
es(network3)[j]]){
        tmp<-p.ig
        network3[i,j]<-rbern(1,tmp)
       }
       if(indiv.info3$Group[indiv.info3$ID==rownames(network3)[i]]!=indiv.info3$Group[indiv.info3$ID==colnam
es(network3)[j]]){
        tmp.d<-dist(group.locs[c(indiv.info3$Group[indiv.info3$ID==rownames(network3)[i]],
                             indiv.info3$Group[indiv.info3$ID==colnames(network3)[j]]),2:3])
        tmp<-p.og*exp(dist.eff*tmp.d)
        network3[i,j]<-rbern(1,tmp)
       }
       network3[j,i]<-network3[i,j]
      }
    }
    diag(network3)<-0

    if(plot==T){
     #dev.new()
     plot(graph.adjacency(network3,mode="undirected"),vertex.color=pop.info[[1]]$I,vertex.label=NA,vertex.si
ze=4)
    }

    networkF<-network3
    indiv.infoF<-indiv.info3
  }
  if(new.indivs==0){
    networkF<-network
    indiv.infoF<-indiv.info2
  }

  recr<-list(indiv.infoF,networkF)
  return(recr)
}
```

In this section of the code we define the key parameters - these are the ones to change to adjust network and disease characteristics

```
#PROBABILITY OF WITHIN SUBPOPULATION CONTACTS
p.ig<-0.5

#PROBABILITY OF BETWEEN SUBPOPULATION CONTACTS
p.og<-0.03

#DISTANCE EFFECT ON BETWEEN SUBPOPULATION CONTACTS
dist.eff<- -0.4 #plugged into exponential decay

#PROBABILITY OF SUCEPTIBLE INDIVIDUAL BEING INFECTED OVER AN EDGE PER TIME-STEP
S_I<-0.06

#MORTALITY OF SUSCEPTIBLE INDIVIDUAL
S_R<-0.01

#ADDITIONAL DISEASE MORTALITY PER TIME-STEP
I_R<-0.07

#BIRTH RATE - provide on logit scale as value desired at stable population size
#BR<-logit(0.5*S_R+0.5*(S_R+I_R))
BR<-logit(S_R*1.01)
```

We now set up the population and its contact network. The population is 100 individuals, consisting of 10 subpopulations of 10 individuals.

Three individuals are infected initially

```
#create empty list to store outputs every time step
pop.info<-list()

n.groups<-10
s.groups<-10

#create initial population
tmp.p<-pop.gen(n.groups,s.groups,n.I=3)

#and store the output
pop.info[[1]]<-tmp.p[[1]]
group.locs<-tmp.p[[2]]

#create intitial network
network<-net.gen(pop=nrow(pop.info[[1]]),indiv.info=pop.info[[1]],group.locs=group.locs,p.ig,p.og,dist.eff,p
lot=T)
```

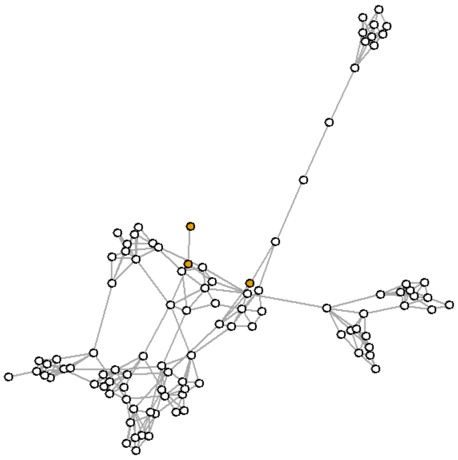

Pre-vaccination phase

We now run transmission (and tranisition to recovered state) for the initial 5 timesteps (see SM1.3)

```
tmp.up<-ts(network=network,indiv.info=pop.info[[1]],S_I=S_I,S_R=S_R,I_R=I_R,plot=F)
tmp.up2<-recruit(network=network,indiv.info=tmp.up[[1]],BR=BR+(100-sum(tmp.up[[1]]$R==0))/1000,group.locs=gr
oup.locs,p.ig=p.ig,p.og=p.og,dist.eff=dist.eff,plot=F)

pop.info[[2]]<-tmp.up2[[1]]
network<-tmp.up2[[2]]

for(t in 3:5){
 #if(sum(tmp.up2[[1]]$I)==0){break}
 tmp.up<-ts(network=network,indiv.info=tmp.up2[[1]],S_I=S_I,S_R=S_R,I_R=I_R,plot=F)
 tmp.up2<-recruit(network=network,indiv.info=tmp.up[[1]],BR=BR+(100-sum(tmp.up[[1]]$R==0))/1000,group.locs=g
roup.locs,p.ig=p.ig,p.og=p.og,
                  dist.eff=dist.eff,plot=F)

 pop.info[[t]]<-tmp.up2[[1]]
 network<-tmp.up2[[2]]
 if(t%%100==0){print(t)}
}

network2<-network
```

We now define our vaccination programme * types = whether vaccination is network-targeted or not * met.set = the metric to use if vaccination is network-targeted * props = the porportion of individuals to vaccinate

```
types<-c("R","R","N","N")
met.set<-c("D","D","D","B")
props<-c(0,0.2,0.2,0.2)
```

Vaccination phase and post-vaccination phase

This code chunk conducts the vaccination and post-vaccination phases 10 times for each of the four vaccination programmes defined above

```
VAC.RES<-list()
pos<-1

for(va in 1:4){
 for(re in 1:50){

  pop.info<-pop.info[1:5]
  network<-network2

  pop.info[[5]]$V<-rep(0,nrow(pop.info[[5]]))

  tmp.up<-ts2(network=network,indiv.info=pop.info[[5]],S_I=S_I,S_R=S_R,I_R=I_R,plot=F)
  tmp.up2<-recruit2(network=network,indiv.info=tmp.up[[1]],BR=BR+(100-sum(tmp.up[[1]]$R==0))/1000,group.locs
=group.locs,
                 p.ig=p.ig,p.og=p.og,dist.eff=dist.eff,plot=F)

  network<-tmp.up2[[2]]

  mets<-met.calc(network,met.set[va])
  tmp.up2[[1]]<-vacc(indiv.info=tmp.up2[[1]],type=types[va],metric=met.set[va],metric.info=mets,v.prop=props
[va],v.eff=1)
  pop.info[[6]]<-tmp.up2[[1]]


  for(t in 2:80){
   tmp.up<-ts2(network=network,indiv.info=tmp.up2[[1]],S_I=S_I,S_R=S_R,I_R=I_R,plot=F)
   tmp.up2<-recruit2(network=network,indiv.info=tmp.up[[1]],BR=BR+(100-sum(tmp.up[[1]]$R==0))/1000,group.loc
s=group.locs,
                  p.ig=p.ig,p.og=p.og,dist.eff=dist.eff,plot=F)

   pop.info[[5+t]]<-tmp.up2[[1]]
   network<-tmp.up2[[2]]

  }

  VAC.RES[[pos]]<-pop.info[6:(t+5)]

  pos<-pos+1
 }
}
```

Data summary and visualisation

This code chunk now summaries the results (may need adjusting if input parameters/simulations are changed)

```
pars<-rep(rep(seq(1,4,1),each=50),80)
reps<-rep(seq(1,50,1),4*80)
tss<-rep(seq(1,80,1),each=4*50)
prevs<-rep(NA,length(pars))
pops<-rep(NA,length(pars))

vac.out<-data.frame(pars,reps,tss,prevs,pops)

pa<-rep(seq(1,4,1),each=50)
rp<-rep(1:50,4)

for(rs in 1:200){

 POPS<-matrix(unlist(lapply(VAC.RES[[rs]],dim)),nr=length(VAC.RES[[rs]]),nc=2,byrow=T)
 POPS<-POPS[,1]

 OUT<-matrix(unlist(lapply(VAC.RES[[rs]],colSums)),nr=length(VAC.RES[[rs]]),nc=8,byrow = T)

 prev<-OUT[,6]/POPS

 vac.out$prevs[which(vac.out$pars==pa[rs]&vac.out$reps==rp[rs])]<-prev
 vac.out$pops[vac.out$pars==pa[rs]&vac.out$reps==rp[rs]]<-POPS

}
```

This code now plots the results (may need adjusting if input parameters/simulations are changed)

```r
sum.vac<-aggregate(vac.out$prevs,by=list(vac.out$tss,vac.out$pars),mean)

col3=c("#4477AA", "#DDCC77", "#CC6677")

colA<-col2rgb("grey")/255
colB<-col2rgb(col3[1])/255
colC<-col2rgb(col3[2])/255
colD<-col2rgb(col3[3])/255

par(mfrow=c(1,2),mar=c(5,5,1,1))

plot(sum.vac[sum.vac[,2]==1,3],xlim=c(0,80),ylim=c(0,0.4),type="l",col="grey",lwd=3,las=1,cex.axis=1.4,cex.lab=1.7,ylab="Disease prevalence",xlab="Time Step")
points(vac.out$tss[vac.out$tss%in%c(20,40,60,80)&vac.out$pars==1&vac.out$reps<26]-1.5,
       vac.out$prevs[vac.out$tss%in%c(20,40,60,80)&vac.out$pars==1&vac.out$reps<26],col=rgb(colA[1],colA[2],colA[3],0.5),pch=4,cex=0.5,lwd=2)
points(vac.out$tss[vac.out$tss%in%c(20,40,60,80)&vac.out$pars==2&vac.out$reps<26]-0.5,
       vac.out$prevs[vac.out$tss%in%c(20,40,60,80)&vac.out$pars==2&vac.out$reps<26],col=rgb(colB[1],colB[2],colB[3],0.5),pch=4,cex=0.5,lwd=2)
points(vac.out$tss[vac.out$tss%in%c(20,40,60,80)&vac.out$pars==3&vac.out$reps<26]+0.5,
       vac.out$prevs[vac.out$tss%in%c(20,40,60,80)&vac.out$pars==3&vac.out$reps<26],col=rgb(colC[1],colC[2],colC[3],0.5),pch=4,cex=0.5,lwd=2)
points(vac.out$tss[vac.out$tss%in%c(20,40,60,80)&vac.out$pars==4&vac.out$reps<26]+1.5,
       vac.out$prevs[vac.out$tss%in%c(20,40,60,80)&vac.out$pars==4&vac.out$reps<26],col=rgb(colD[1],colD[2],colD[3],0.5),pch=4,cex=0.5,lwd=2)
lines(sum.vac[sum.vac[,2]==1,3],col="grey",lwd=3)
lines(sum.vac[sum.vac[,2]==2,3],col=col3[1],lwd=3)
lines(sum.vac[sum.vac[,2]==3,3],col=col3[2],lwd=3)
lines(sum.vac[sum.vac[,2]==4,3],col=col3[3],lwd=3)

sum.vac2<-aggregate(vac.out$pops,by=list(vac.out$tss,vac.out$pars),mean)

plot(sum.vac2[sum.vac2[,2]==1,3],xlim=c(0,80),ylim=c(20,140),type="l",col="grey",lwd=3,las=1,cex.axis=1.4,cex.lab=1.7,ylab="Host population size",xlab="Time Step")

points(vac.out$tss[vac.out$tss%in%c(20,40,60,80)&vac.out$pars==1&vac.out$reps<26]-1.5,
       vac.out$pops[vac.out$tss%in%c(20,40,60,80)&vac.out$pars==1&vac.out$reps<26],col=rgb(colA[1],colA[2],colA[3],0.5),pch=4,cex=0.5,lwd=2)
points(vac.out$tss[vac.out$tss%in%c(20,40,60,80)&vac.out$pars==2&vac.out$reps<26]-0.5,
       vac.out$pops[vac.out$tss%in%c(20,40,60,80)&vac.out$pars==2&vac.out$reps<26],col=rgb(colB[1],colB[2],colB[3],0.5),pch=4,cex=0.5,lwd=2)
points(vac.out$tss[vac.out$tss%in%c(20,40,60,80)&vac.out$pars==3&vac.out$reps<26]+0.5,
       vac.out$pops[vac.out$tss%in%c(20,40,60,80)&vac.out$pars==3&vac.out$reps<26],col=rgb(colC[1],colC[2],colC[3],0.5),pch=4,cex=0.5,lwd=2)
points(vac.out$tss[vac.out$tss%in%c(20,40,60,80)&vac.out$pars==4&vac.out$reps<26]+1.5,
       vac.out$pops[vac.out$tss%in%c(20,40,60,80)&vac.out$pars==4&vac.out$reps<26],col=rgb(colD[1],colD[2],colD[3],0.5),pch=4,cex=0.5,lwd=2)
lines(sum.vac2[sum.vac2[,2]==1,3],col="grey",lwd=3)
lines(sum.vac2[sum.vac2[,2]==2,3],col=col3[1],lwd=3)
lines(sum.vac2[sum.vac2[,2]==3,3],col=col3[2],lwd=3)
lines(sum.vac2[sum.vac2[,2]==4,3],col=col3[3],lwd=3)
```

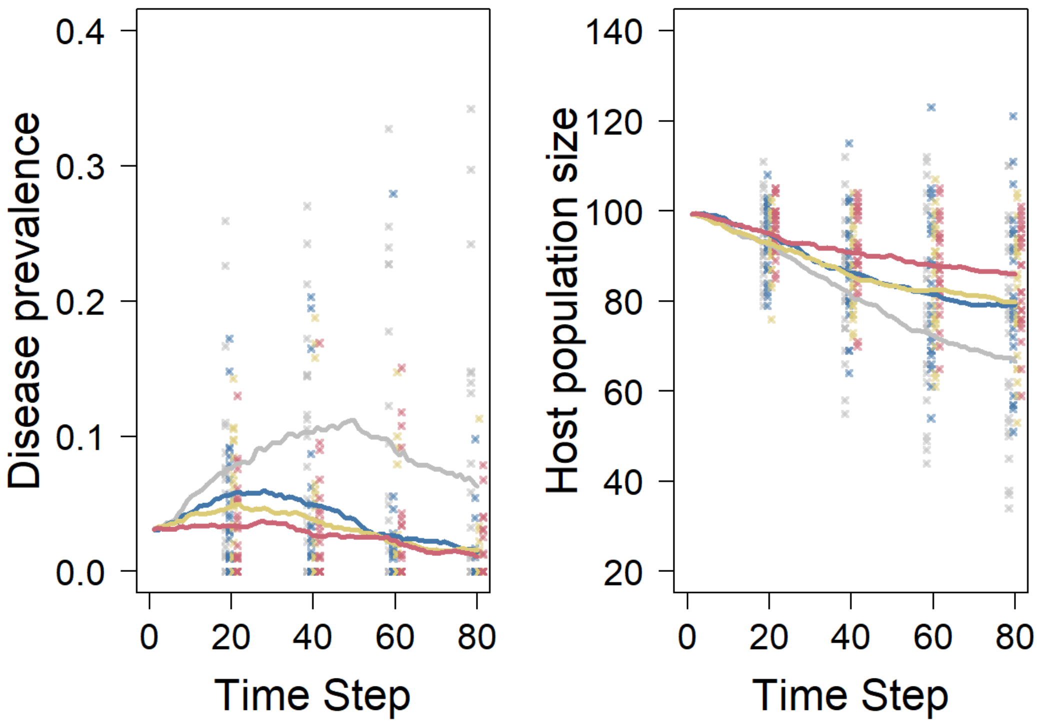

Disease prevalence
0.0
0.1
0.2
0.3
0.4
Time Step
0
20
40
60
80
Host population size
20
40
60
80
100
120
140
Time Step
0
20
40
60
80
